# Two coinfecting archaeal viruses provide insights into virus-virus interactions

**DOI:** 10.1038/s42003-026-11061-7

**Published:** 2026-09-24

**Authors:** Lauren Queiss, Coraline Mercier, Tomas Alarcón-Schumacher, Grace D’Angelo, Federico Agustin Vignale, Judith M. Klatt, Manuel Liebeke, Manuel Contreras, María Eugenia Farías, Susanne Erdmann

**Affiliations:** 1 Max-Planck Institute for Marine Microbiology, Bremen, Germany; 2 European Molecular Biology Laboratory – Hamburg Unit, Hamburg, Germany; 3 Microcosm Earth Center, Max Planck Institute for Terrestrial Microbiology, Philipps-Universität Marburg, Marburg, Germany; 4 Center for Synthetic Microbiology, SYNMIKRO, Marburg, Germany; 5 Biogeochemistry Group, Department of Chemistry, Marburg, Germany; 6 Department for Metabolomics, Kiel University, Kiel, Germany; 7 Centro de Ecología aplicada (CEA) Av. Príncipe de Gales 6465, Santiago, Región Metropolitana Chile; 8 PUNABIO S.A., Campus USP-T Av., Solano Vera y Camino a Villa Nougués, Tucumán, Argentina; 9 Institute for Microbiology, University of Innsbruck, Innsbruck, Austria

**Keywords:** Virus-host interactions, Archaeal biology, Water microbiology, Phage biology

## Abstract

Virus–virus interactions can profoundly shape viral ecology and evolution, yet they remain poorly understood, particularly in haloarchaea. Upon characterization of a spindle-shaped virus, Tebenquiche spindle-shaped virus 1 (Tebi-SV1), isolated from *Halorubrum* strain TLS-6, we detected the presence of a second virus, Tebenquiche pleomorphic virus 1 (Tebi-PV1), that coinfects host cells. Gene sharing network analysis revealed several genomes related to Tebi-SV1 in public databases, most of which also co-occur with pleolipoviruses in their host genomes. Attempts to separate the two viruses were unsuccessful, suggesting a symbiotic relationship between Tebi-SV1 and Tebi-PV1. We discovered that virus abundances may be regulated through a shared operator sequence allowing Tebi-PV1 to keep Tebi-SV1 copy numbers low without excluding Tebi-SV1 from the host genome. Additionally, Tebi-PV1 encodes and expresses a type-4-pilin, that likely plays a role in regulating the interactions between the host and the viruses. Our findings highlight the complexity of virus-virus interactions and that resident viruses are important regulators of virus-host interactions.

## Introduction

Virus coinfections occur in various environments and play significant roles in viral evolution and ecology across all three domains of life. Up to 35% of infected bacterial cells in a single-cell genomic dataset and nearly 50% of microbial genomes from public archaeal and bacterial datasets contain more than one virus^1,2^. Virus-virus interactions have been shown to influence viral infectivity, alter the host range and lifecycle, alter host physiology, and facilitate genetic exchange^2–8^.

The majority of studies on virus-virus interactions in prokaryotes describe antagonistic relationships between coinfecting viruses. Viruses can exhibit mechanisms driving superinfection exclusion, where one virus prevents infection or replication of a superinfecting virus to maintain control over the host organism’s resources. This phenomenon is well studied in bacteriophages^9,10^ and has also been described more recently in archaeal viruses^11–14^. Archaeal viruses and other mobile genetic elements (MGEs) were shown to carry mini-CRISPR arrays as a distinct superinfection exclusion mechanism^13,15^. Induction of CRISPR spacer acquisition against coinfecting viruses and a plasmid was described for SMV1^16,17^. Coinfection experiments with bicaudaviruses SMV1 and STSV2 triggered the CRISPR adaptation of the host against STSV2, which resulted in exclusion of STSV2 while SMV1 persisted in the host^17^.

Viral coinfections in archaeal hosts are not limited to these antagonistic dynamics. Two proviruses in *Aeropyrum pernix*, unclassified spindle-shaped virus, APSV1, and guttavirus, APOV1, were simultaneously induced with no apparent cell lysis under suboptimal oxic conditions^18–20^. In another example, rudivirus SIRV3 and bicaudavirus SMV1 were stably maintained in *Sulfolobus islandicus* REY15A for nearly two weeks^21^. Interestingly, in neither case did the infection have detrimental effects on the host.

Studies on commensal coinfections—where the presence of one entity ensures the success of another—are limited but those that explore this phenomenon typically describe subviral agents such as satellite viruses and helper viruses^22–25^. Increased infection rates have been described for bacteriophage Ф6 upon infection of cells that were already infected by the same virus^26^. Coinfection of the same host cell with different genomic variants of the same virus was shown to lead to recombinant viral genomes with altered virus-host relationships^27,28^. The majority of commensal interactions have been observed between viruses and plasmids, with viruses supporting the distribution of plasmids^29–32^ and plasmids supporting viral infection^33^. Coinfections with different types of MGEs have rarely been described in Archaea, with most studies involving spindle-shaped viruses. In the hyperthermophilic archaeon *Thermococcus prieurii*, spindle-shaped virus TPV1 coexists with two plasmids pTP1 and pTP2, without antagonistic effects within the same host^34^. A commensal relationship has been described in a *Sulfolobus islandicus* strain where a small plasmid-virus hybrid, pSSVx, is packaged in a spindle-shaped virion and requires co-transfection with a helper spindle-shaped virus, SSV1 or SSV2, to proliferate successfully^29^.

While coinfection dynamics are well-studied in temperate and hyperthermophilic environments, the dynamics of virus-virus interactions in hypersaline environments remain largely unexplored. In halophilic archaea, only one example of coinfection has been investigated: SNJ1 (sphaerolipovirus) and SNJ2 (pleolipovirus), coinfecting *Natrinema* sp. J7^12,35^. Most recently it was shown that SNJ2 provides the receptor for SNJ1, while SNJ1 supports the replication of SNJ2 providing evidence for a mutualistic interaction between the two viruses^36^.

Here we report the co-isolation and characterization of two viruses from a halophilic archaeal host. We isolated a spindle-shaped virus, Tebi-SV1, and a pleomorphic virus, Tebi-PV1, from the same strain, *Halorubrum* sp. TLS-6 (*Hrr*. TLS-6)^37^. *Hrr*. TLS-6 was isolated from Laguna Tebenquiche, a hypersaline lagoon at 2000 m above sea level, exposed to extreme solar radiation and enriched in volcanic-derived arsenic (up to 5 mg/L), lithium (~145–445 mg/L), and boron (~158–543 mg/L)^37–39^. Spindle-shaped virus Tebi-SV1 belongs to an undescribed family of spindle-shaped viruses. Despite their abundance in hypersaline systems, only two halophilic spindle-shaped viruses, His1 and LSV-48N, have been described to date^40,41^. His1 is currently the sole member of the virus family *Halspiviridae*, and shares little to no sequence homology to other spindle-shaped virus families^42^, but the structure of the major capsid protein (MCP) is similar to that of other spindle-shaped viruses^43^. Our study reveals that Tebi-SV1 seems to be mutually regulated by the coinfecting virus, Tebi-PV1.

## Results

### Discovery of virus-like particles in the supernatant of an isolated Halorubrum strain

**Fig. 1:**
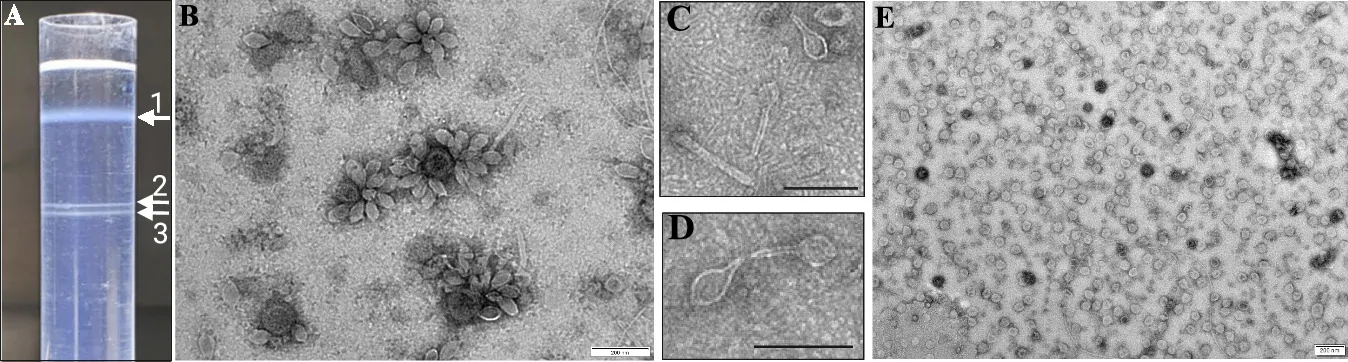
Electron micrographs of virus-like particles isolated from *Hrr*. TLS-6 culture supernatants. **A** 40-25% Iodixanol density gradient of the concentrated culture supernatant from growth retarded *Hrr*. TLS-6 (first initial virus preparation). Three resulting bands are highlighted by arrows and numbered. **B** Electron micrograph of spindle-shaped VLPs. The sample derived from the second band (2) of the Iodixanol gradient in (**A**) that was highly enriched with spindle-shaped VLPs (size bar 200 nm). **C**, **D** Spindle-shaped VLPs exhibiting pleomorphism with elongated tails and in a tubular form from band 1 of the Iodixanol gradient in (**A**) (size bars are 100 nm in (**C**, **D**). **E** Electron micrographs of concentrated supernatant enriched with Tebi-PV1 particles from *Hrr*. TLS-6 grown in nutrient rich media at 28 °C.

While attempting to grow a colony of strain *Halorubrum* sp. TLS-6 (*Hrr*. TLS-6)^37^ in liquid media, we observed a deviation from its typical growth behavior (Supplementary Fig. 1). The culture failed to reach exponential phase and did not exceed an OD_600 nm_ of 0.08, indicative of severe growth inhibition. Analysis of the concentrated supernatant of the culture by transmission electron microscopy (TEM) revealed the presence of spindle-shaped virus-like particles (VLPs) (Fig. 1). The particles appear pleomorphic with a short tail at one end, similar to His1^40,44^. Particles with a regular spindle-shape were approximately 110 nm in length and 50 nm in diameter (Fig. 1B), slightly larger than what has been described for His1^40^. We also observed VLPs with an elongated tubular morphology of approximately 200 nm in length (Fig. 1C, D), which we suggest could represent particles of the same virus with the genetic material ejected, as described for the spindle-shaped virus His1^44,45^. We purified the concentrated supernatant on an Iodixanol density gradient and examined the three resulting bands by TEM (Fig. 1). The first band contained mostly elongated spindle-shaped VLPs, while the third band contained mainly cellular debris and membranous vesicles. The second band (Fig. 1A) contained mostly intact spindle-shaped VLPs (Fig. 1B). We extracted total DNA from this band for high-throughput sequencing. It is important to note that such a high abundance of the spindle-shaped VLP was not observed in any of the following virus preparations.

### Sequencing of total DNA from purified virus particles reveals two viral genomes

**Fig. 2:**
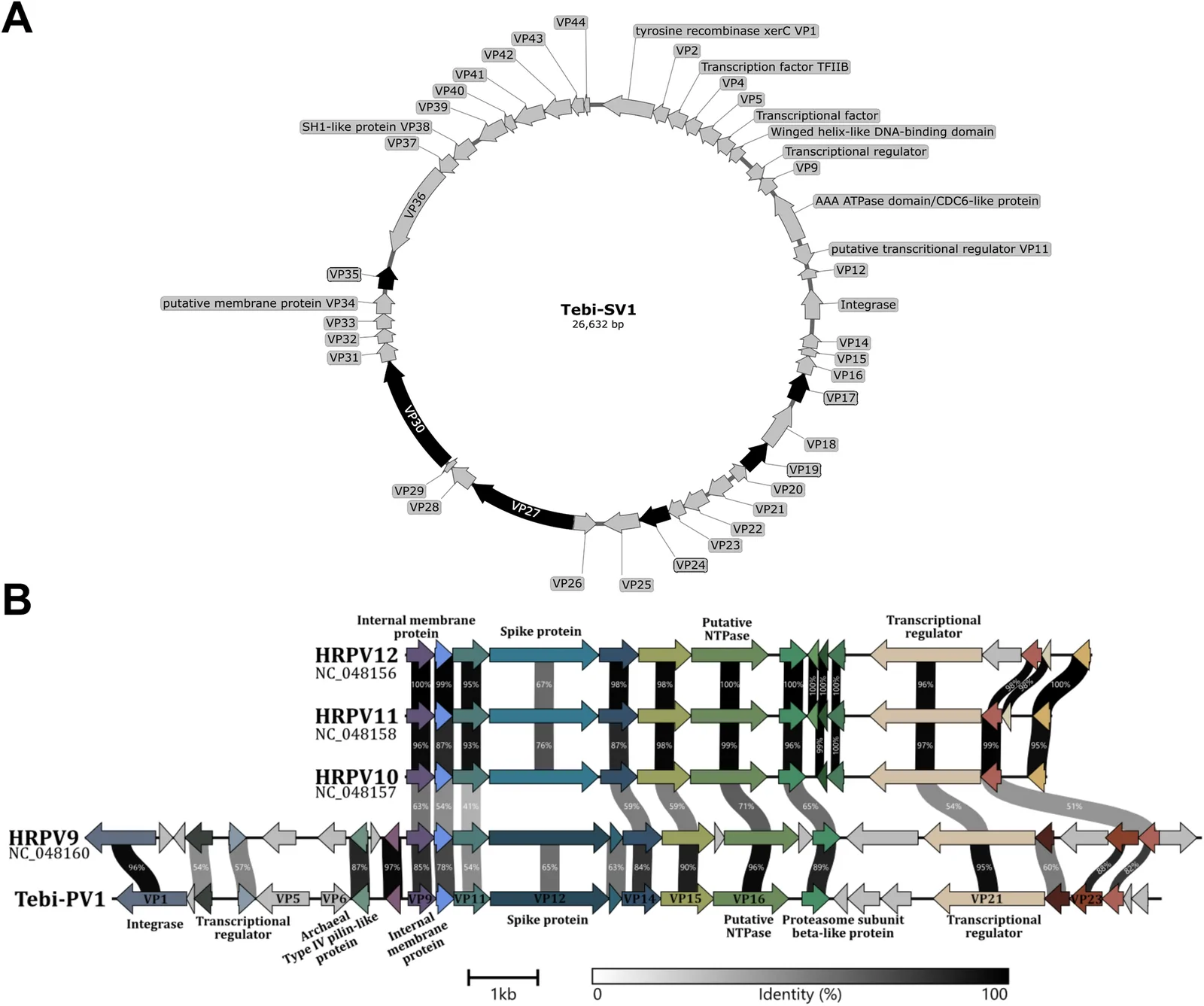
Genomic characteristics of Tebi-SV1 and Tebi-PV1. **A** Genome map (SnapGene software (www.snapgene.com)) of Tebi-SV1 with annotations. Proteins detected in purified virus particles are shown in black. **B** Tebi-PV1 genome comparison with closest pleolipovirus relatives. Homologous genes are shown in the same colors, with percent amino acid identity represented vertically between genes^97^.

Following Illumina sequencing, we assembled two distinct circular contigs of 26.6 kbp and 15.4 kbp. The larger contig (26.6 kb) encodes for 44 open reading frames (ORFs) (Fig. 2A), with an average GC content of 62.8% (GC content of host chromosome being 69.8%) and a median coverage depth of 12,000. Based on the observation of spindle-shaped particles in this band (Fig. 1B), we infer that this contig corresponds to the spindle-shaped virus. We named the virus Tebi-SV1, Tebi for Laguna Tebenquiche (place of isolation), and SV for spindle-shaped virus.

The smaller contig (15.4 kb) encodes for 26 ORFs, with a GC content of 62.4% and a median coverage of 100. The contig displays the highest similarity (52% nucleotide sequence pairwise identity) to Halorubrum pleomorphic virus 9 (HRPV9)^46^. It encodes for the conserved set of proteins characteristic of members of the *Pleolipoviridae* family, including the spike protein (VP12 [viral protein 12]) and an internal membrane protein (VP9) (Fig. 2B). It also encodes for an integrase (VP1), which is consistent with our observation of an integration site on the host genome (Supplementary text) (Fig. 4A). VP4 and VP21 are transcriptional regulators and VP16 is an NTPase. VP4 contains an ArsR-like helix-turn-helix domain (IPR011991), which is found in metal (including arsenic) regulated repressors^47^, and the predicted structure shows significant similarity to a PhiH-like repressor protein (3B73^48^, z-score 13.9, rmsd 1.9). The contig also encodes for an archaeal type-IV pilin-like protein (VP6). Gene sharing network analyses reveal that the virus genome belongs to the *Betapleolipovirus* genus^49^ (Fig. 2, Suppl. Fig. 2). We concluded that this genome represents a pleolipovirus, and named it Tebi-PV1.

### The spindle-shaped virus Tebi-SV1 belongs to the proposed family Almyrocitroviridae

**Fig. 3:**
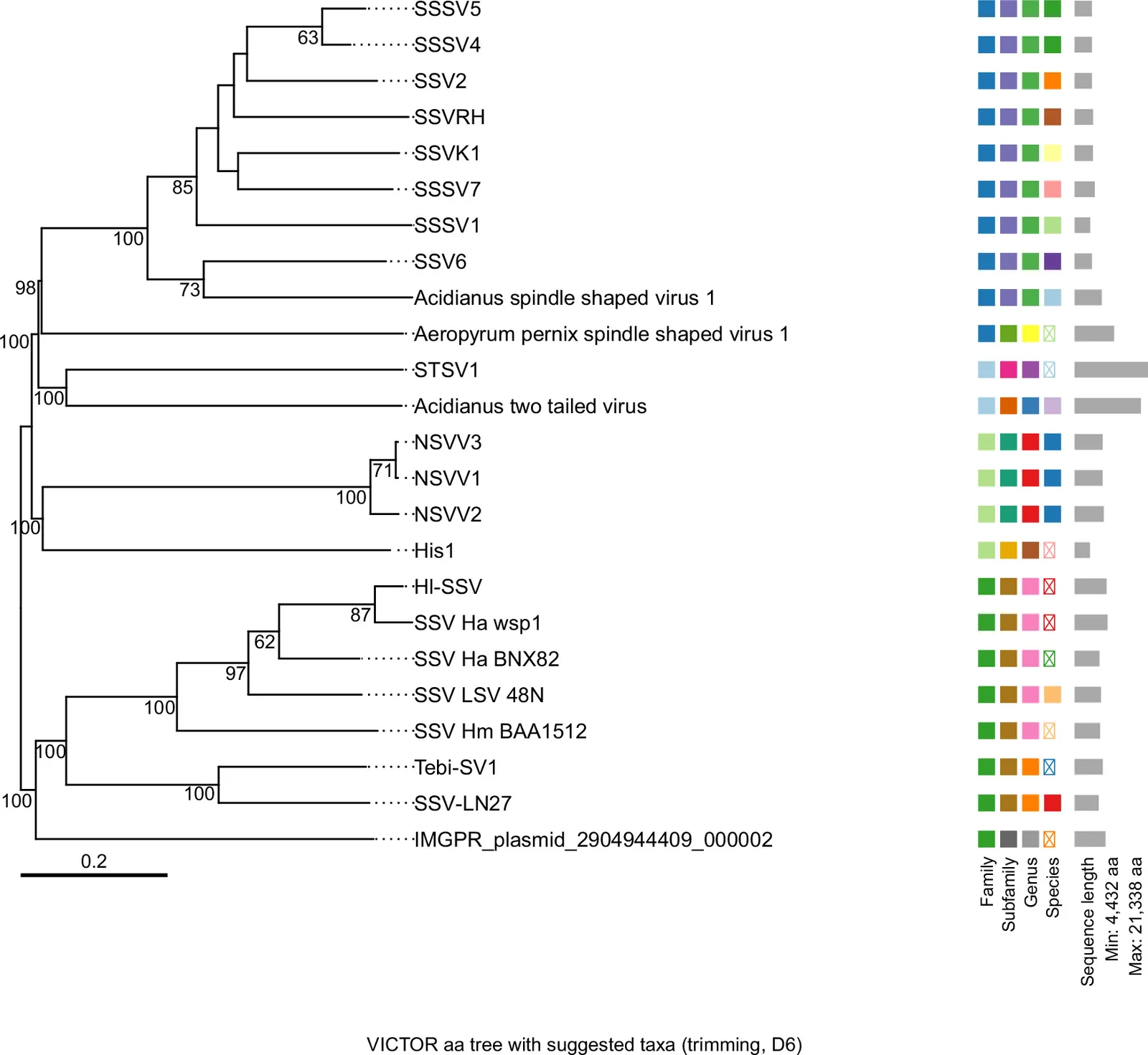
Genome-based phylogeny classification analyses. VICTOR tree using the predicted protein sequences of Tebi-SV1, SSV-LN27, IMGPR_plasmid_2904944409_000002, together with the spindle-shaped viruses described by Turgeman-Grott et al.^61^, and other previously described spindle-shaped viruses (of families *Halspiviridae, Thaspiviridae*, *Fuselloviridae*, and *Bicausaviridae*) using the D6 formula. The scale bar represents distances inferred from whole genome blast comparisons. Colored squares represent taxonomic classification estimated by VICTOR algorithm (from left to right: Family, Subfamily, Genus and Species). Accession numbers of all genomes included in the analysis are presented in Suppl. Table 1 (Suppl. Data file 1).

The genome of Tebi-SV1 exhibited no significant similarity at the nucleotide or amino acid level to any characterized virus isolate or genome from the IMG/VR database^50^. However, multiple hypothetical proteins of Tebi-SV1 were homologous to sequences from *Halorubrum* sp. LN27, a strain isolated from Dingyuan Salt Mine in China^51^, suggesting a similar virus may be integrated into this genome. Tebi-SV1 and the putative provirus in LN27 (referred to as SSV-LN27) have an average nucleotide identity (ANI) of 82%, which is well below the typical threshold used to delineate species in viral genomics^52^. Genome-based phylogeny classification of spindle-shaped viruses (Fig. 3) places Tebi-SV1, SSV-LN27, a proposed plasmid from the IMPGR database (IMGPR_plasmid_2904944409_000002), and the spindle-shaped viruses described by Turgeman-Grott et al.^41^ into a virus family, for which we propose the name *Almyrocitroviridae* (Almyro- Greek for salty, and -citro for lemon).

Additionally, gene-sharing network analysis (Suppl. Fig. 2, Suppl. Data file 1: Suppl. Table 2) and genome comparison (Suppl. Fig. 3) supports that the members of this family correspond to at least two different genera, with one comprised by the LSV-48N virus and its relatives and a second genus including Tebi-SV1 and SSV-LN27.

The majority of the 44 predicted gene products on the Tebi-SV1 genome are hypothetical proteins, and even when using predicted tertiary structures, only a few could be annotated. VP1 is a predicted tyrosine recombinase, and VP3 is a putative transcription initiation factor (TFB). VP6 and VP8 are predicted to be transcriptional regulators (helix-turn-helix), and VP7 contains a winged helix-like DNA-binding domain. The predicted tertiary structure of VP8 shows significant similarity to a PhiH-like repressor protein (3B73^48^, z-score 11.1, rmsd 1.5), similar to VP4 of Tebi-PV1. Alignment of the predicted structures of VP8 and Tebi-PV1 VP4 reveals that they share a high structural similarity (z-score 13.9, rmsd 1.2). VP10 is a CDC6-like protein, VP13 is a second tyrosine recombinase, and VP29 is a large IG-like domain-containing protein with a transmembrane and a sugar-binding domain (Fig. 2A).

**Fig. 4:**
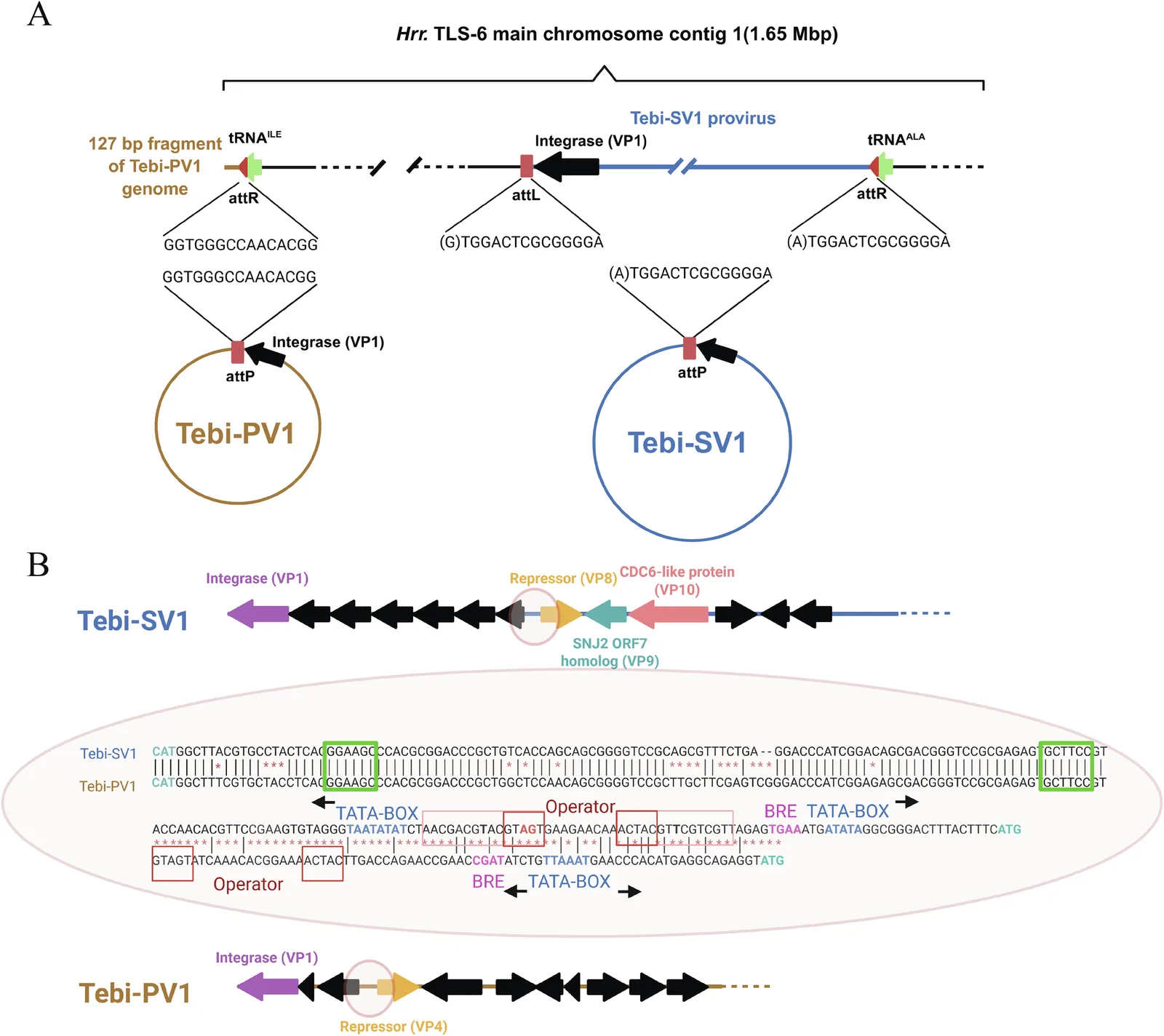
Schematic presentation of integration sites and promotor regions of Tebi-SV1 and Tebi-PV1. **A** Tebi-SV1 was found integrated into a contig of the main chromosome of *Hrr*.TLS-6. Attachment sites (attR, attL, and attP) are illustrated with the respective sequence. Tebi-PV1 was assembled as an extrachromosomal element. However, a 127 bp fragment upstream of the integrase was identical to the upstream end of the same contig and represent the integration site of Tebi-PV1. **B** Alignment of the regulatory region between the integrase operon and the repressor of Tebi-SV1 and Tebi-PV1. Proposed start codons are highlighted in turquoise. The inverted repeats of each operator are conserved between Tebi-SV1 and Tebi-PV1 (highlighted with a dark red box). The operator of Tebi-SV1 may exceed the 5 bp repeat shared between Tebi-SV1 and Tebi-PV1 (light red box). Tebi-SV1 exhibits proposed promotors (TATA-box and B recognition element, BRE) for each the integrase operon and the repressor protein. For Tebi-PV1, we could only identify one potential TATA-box, that likely serves as promotor in both directions. Proposed directions of transcription are indicated with arrows. The Tebi-SV1 genome retrieved from the first isolation, with Tebi-SV1 dominating, exhibits two SNPs within the operator sequence that are highlighted in red. Both viruses exhibit a conserved 5’UTR upstream of the integrase operon that is indicated by sequence similarity in the alignment. The 5’UTR has a high self-binding tendency, and the binding sites are highlighted with green boxes. Figure created with BioRender.com.

Tebi-SV1 is integrated as a provirus in the host chromosome upstream of an alanine tRNA gene, with the integrase (VP1) at the upstream boundary of the provirus and VP44 next to the tRNA (Supplementary text). A 14 bp stretch at the upstream insertion site of the integrated provirus is identical to the 3’ end of the tRNA flanking the provirus downstream, representing the attachment sites attL and attR. This repeat is present only once on the circularized virus genome, representing the virus attachment site (attP) (Fig. 4A). The genome isolated from purified Tebi-SV1 particles shares only 99% identity with the provirus. We detected two significant single nucleotide polymorphisms (SNP), at positions 3248–3249, on the virus genome from this preparation when compared to the integrated virus in the assembled host genome (Supplementary text). Only 21.2% of the reads mapped to the wild-type Tebi-SV1 at these positions in the host chromosome; the remaining reads either had a deletion of A in position 3248 or a deletion of G in position 3249. Both SNPs alter the operator that may regulate the expression of the downstream gene (VP8) and also potentially the expression of the upstream transcript (Fig. 4B). To determine whether these changes occurred on the host chromosome during continuous cultivation of the host strain, we re-sequenced the host strain. While a few SNPs (46 SNPs, frequency between 25% and 48.5%, mean coverage 319) were detected when comparing the *Hrr*. TLS-6 genome from 2021 and 2023, none of them were detected within the provirus region. Therefore, we conclude that these changes only occurred in the host strain colony that was used to grow the growth-retarded culture from which we isolated Tebi-SV1.

### Tebi-PV1 dominates viral preparations and cannot be separated from Tebi-SV1

The first virus preparation from growth retarded *Hrr*. TLS-6 cultures had a high Tebi-SV1 to Tebi-PV1 ratio of 1200:1 (based on sequencing coverage) in the gradient band subjected to sequencing (band 2 in Fig. 1A). However, such a strong growth inhibition was not reproducible in any subsequent cultivations of *Hrr*. TLS-6; neither when grown from colonies from the same plate, when grown from the original glycerol stock, nor upon reinfection with viral particles isolated from the original isolate. Genome copy number per mL ratios (assessed by qPCR in at least 8 biological replicates) between Tebi-PV1 and Tebi-SV1 averaged at about 1760:1. Attempts to induce Tebi-SV1 production revealed that Tebi-PV1 particles are particularly abundant in the supernatant of cultures grown in DBCM2+ media (high nutrient medium), and Tebi-SV1 particles are enriched by over 50-fold in CDM media (low nutrient medium) supplemented with 1 mM PO_4_, in comparison with DBCM2+ (*p*-value of 4.89E-11) (Suppl. Fig. 4, Suppl. Table 3). However, none of the tested conditions resulted in a virus preparation with only one of the two viruses. Physical separation with various density gradients also failed to separate the viruses (Suppl. Fig. 5, Suppl. Data file 1: Suppl. Table 4).

**Fig. 5:**
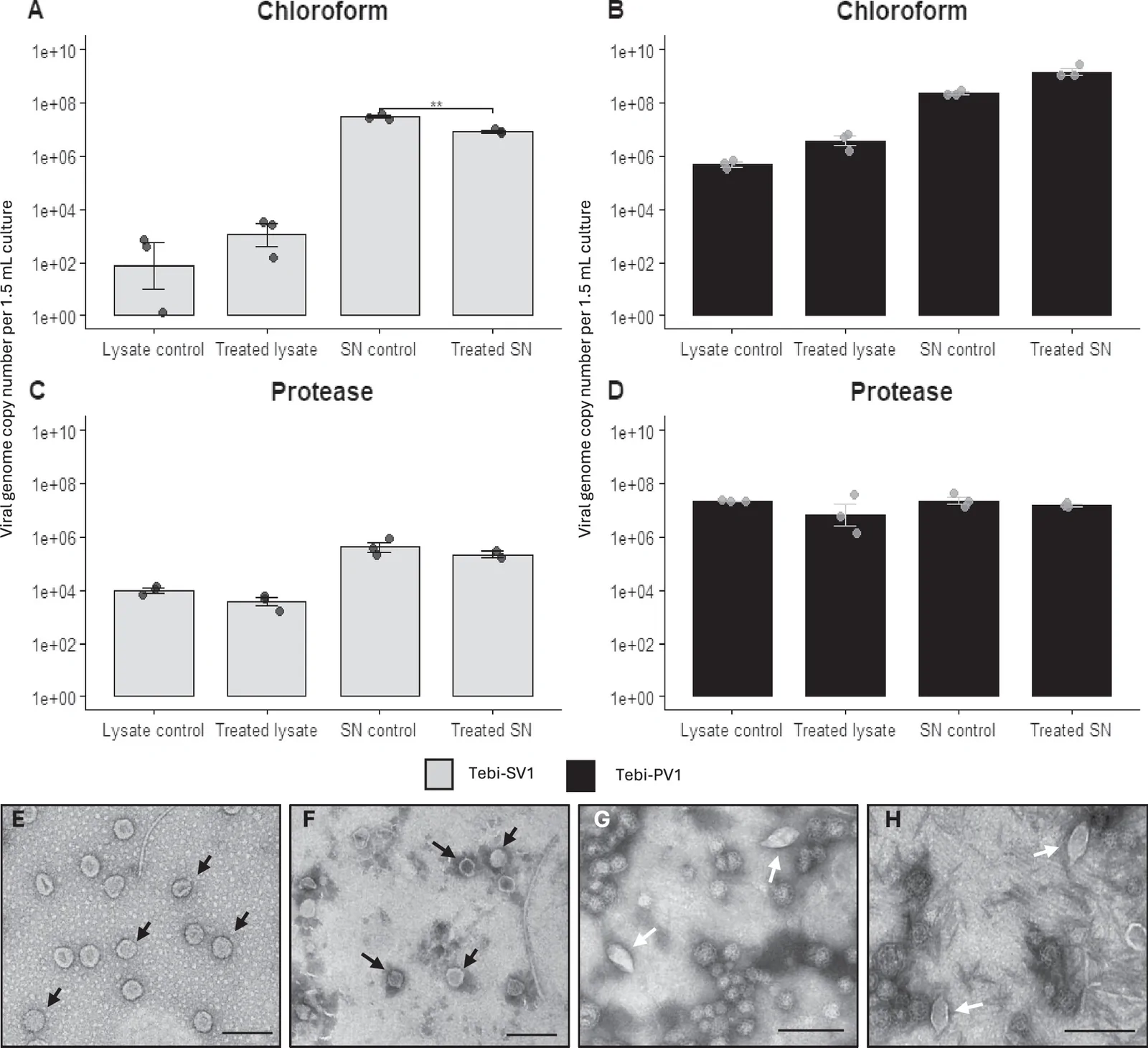
Proteinase K and chloroform treatment of virus preparations. Proteinase K (7.5 mg/ml) and chloroform (1:4 chloroform to sample ratio) treated virus preparations from TLS-6 supernatant (enriched in Tebi-PV1) and TLS-6 artificial cell lysate (enriched in Tebi-SV1) were used to infect *Hrr. lacusprofundi*. Virus activity was compared by determining cell-associated viral genome copy numbers by qPCR. Cell-associated genome copy number of Tebi-SV1 (**A**) and Tebi-PV1 (**B**) per 1.5 mL culture infected with chloroform-treated and untreated (control) viral preparations. Cell-associated genome copy number of Tebi-SV1 (**C**) and Tebi-PV1 (**D**) per 1.5 mL culture infected with proteinase K-treated and untreated (control) viral preparations. **A**–**D** The means of *n* = 3 biologically independent experiments are shown as bars with individual data points plotted and the error bars represent the standard deviation of n = 3 biologically independent experiments. Statistical significance was determined using a two-sided t-test and is indicated with the following significance codes: ‘*’ for *p*-value ≤ 0.05, ‘**’ for *p*-value < 0.01, and ‘***’ for *p*-value < 0.001. For (**A**) ‘**’ *p*-value = 0.02376165. Lysate – virus particles isolated from manually disrupted cells (enriched in Tebi-SV1). SN – virus particles isolated from culture supernatants (enriched in Tebi-PV1). **E** Electron micrograph of untreated virus preparations from TLS-6 supernatant (enriched in Tebi-PV1) and (**G**) Electron micrograph of virus preparations from TLS-6 artificial cell lysate (enriched in Tebi-SV1). **F** Electron micrograph of the chloroform-treated viral preparation with black arrows denoting Tebi-PV1 virions. **H** Electron micrograph of the protease-treated viral preparation with white arrows denoting Tebi-SV1 virions. Scale bars are 200 nm.

Finally, we tested the chloroform and protease sensitivity of virus particles. Since no virus-free *Hrr*. TLS-6 strain was available, we used *Halorubrum lacusprofundi* (*Hrr. lacusprofundi*) as a “virus-free” control strain for infectivity assays. Neither protease nor chloroform treatment inactivated Tebi-SV1 or Tebi-PV1. Protease treatment did not lead to an obvious deformation of Tebi-SV1 particles (Fig. 5H), but reduced infectivity in *Hrr. lacusprofundi* significantly by ~1.8 fold (Fig. 5C). Tebi-PV1 was less sensitive to protease treatment, with a ~ 1-fold reduction of cell-associated genome copy numbers (Fig. 5D). Stability of virus particles despite proteinase K treatment is not only common for viruses with a lipid envelope^53^. Following chloroform treatment, Tebi-SV1 virions had a > 2-fold reduction (Fig. 5A), and Tebi-PV1 had a 0.1-fold reduction of infectivity (Fig. 5B). Despite their lipid membrane, it was shown previously that virions of some pleolipoviruses remain stable after chloroform treatment^54,55^.

### Tebi-PV1 and Tebi-SV1 share the same regulatory elements

Since an increase in Tebi-SV1 abundance leads to a decrease in Tebi-PV1 abundance, we searched for regulatory elements that may allow a co-regulation of the two viruses. We identified four proteins with high sequence similarity between Tebi-PV1 and Tebi-SV1: the predicted repressor proteins (Tebi-SV1 VP8 and Tebi-PV1 VP4), a predicted endonuclease (Tebi-SV1 VP38, Tebi-PV1 VP23), two small proteins with unknown function (Tebi-SV1 VP38, VP41; Tebi-PV1 VP22 and VP26, respectively) and the integrases (VP1 in both genomes) (Suppl. Fig. 6). Additionally, both viruses share a conserved region (88% nucleotide identity) between the integrase operon and Tebi-SV1 VP8 and Tebi-PV1 VP4 (Fig. 4B). The modelled tertiary structure is highly conserved between Tebi-PV1 VP4 and Tebi-SV1 VP8 (z-score 13.9, rmsd 1.2) and with ORF4 (z-score 12.1, rmsd 2.1), the experimentally confirmed repressor protein of SNJ2^56^, suggesting that all three proteins work similarly. We identified the promotors for VP8, VP4, the integrase operons (Fig. 4B), and the repressor binding sites (operator). Intriguingly, the first 5 bp of each of the inverted repeats of the Tebi-SV1 operator is identical to the inverted repeat of the Tebi-PV1 operator, suggesting that the repressor protein of Tebi-PV1 could also bind the operator of Tebi-SV1. However, neither the promotors nor the operators are located in the conserved region upstream of the integrase operon. This conserved region is located between the start codon of the first ORF in the integrase operon and the putative promotor (Fig. 4B). We could not identify a conserved ORF, suggesting that this region may represent a 5’ untranslated region (5’UTR). Indeed, secondary structure prediction^57^ and target prediction^58^, suggest a strong self-binding tendency, with the 5’ end being complementary to the 3’ end (Fig. 4B, Supplementary Fig 7). This putative 5’UTR is also conserved across SNJ2 and LN27-SSV upstream of their respective integrase operons, further supporting an important conserved function (Suppl. Fig. 7).

### Host range determination indicates attachment and initial virus replication in a broad range of host organisms

**Fig. 6:**
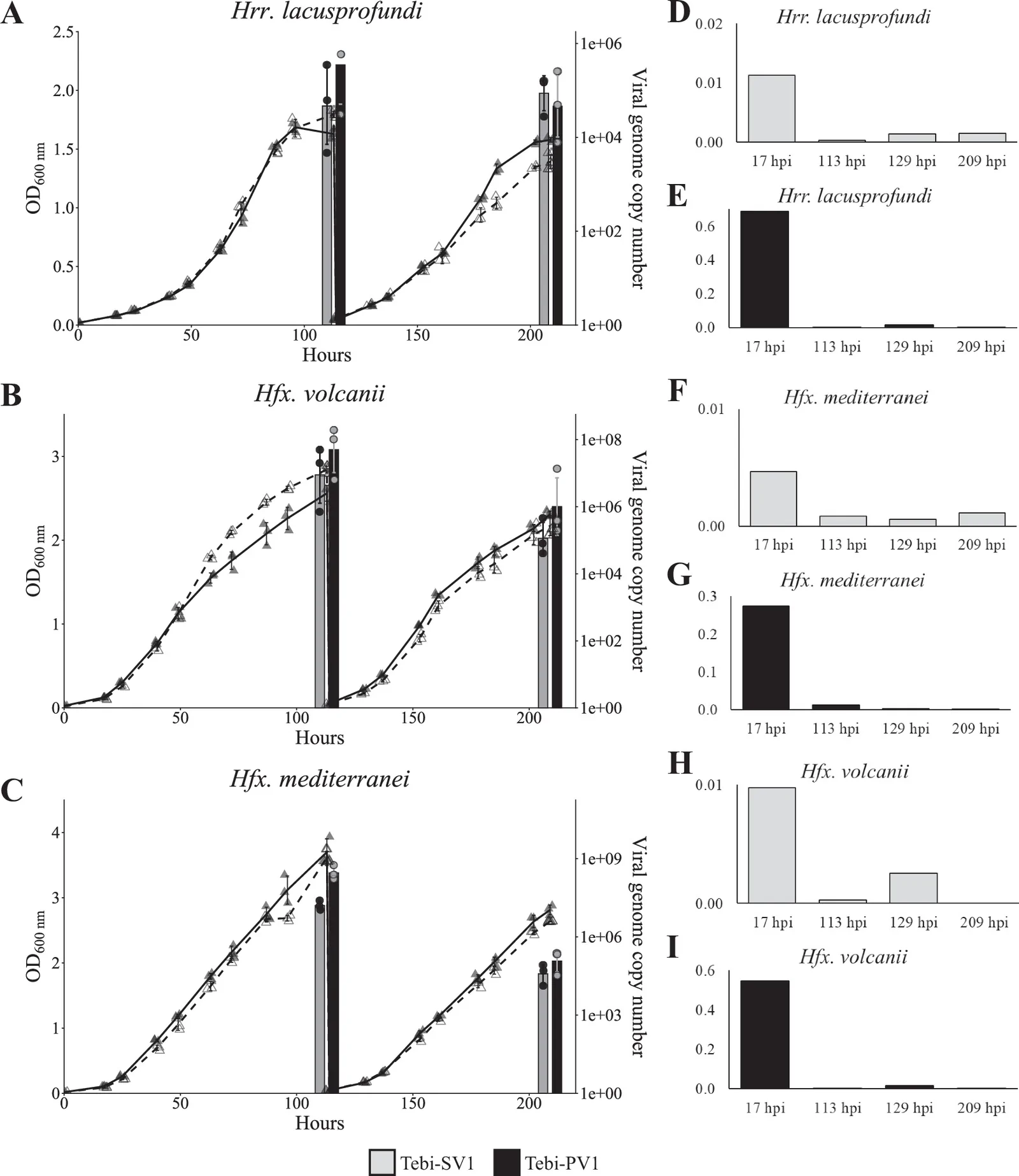
Determination of the host range. **A**–**C** Growth curves of infected (broken line) and uninfected (solid line) of three tested host organisms. After reaching stationary phase, cultures were diluted to a starting OD_600nm_ of 0.05 and allowed to regrow without additional virus added. **A**–**C** The means of *n* = 3 biologically independent experiments are shown as bars (viral genomes per 1.5 mL culture) and lines (for OD_600nm_) with individual data points plotted and the error bars represent the standard deviation of *n* = 3 biologically independent experiments. Grey bars represent the number of Tebi-SV1 genomes per 1.5 mL culture, and black bars represent Tebi-PV1 genomes per 1.5 mL culture detected in the supernatant at 117 and 209 hpi. Error bars represent the standard deviation of *n* = 3 biologically independent experiments. **D**–**I** Cell-associated virus-to-host genome copy number was detected at different time points during the growth curves presented in (**A**–**C**). The number of Tebi-SV1 genomes, Tebi-PV1 genomes, and host chromosome copy numbers were determined by qPCR in cells of 1.5 mL culture. The ratio was determined by dividing the average of Tebi-SV1/Tebi-PV1 genome copy numbers by the average of the host chromosome numbers. Any value > 1, indicates that the number of Tebi-SV1 or Tebi-PV1 genome copy numbers are higher than the host chromosome copy numbers. Grey bars represent Tebi-SV1 and black bars represent Tebi-PV1.

To find a virus free host and further attempt to separate Tebi-SV1 and Tebi-PV1, we infected seven different haloarchaeal strains with a total virus genome number to host cell ratio of approximately 2.5 (Tebi-PV1:Tebi-SV1 ratio ~28:1). After allowing initial adsorption (see methods), we detected the genomes of both Tebi-SV1 and Tebi-PV1 associated with cells (Supplementary text) by qPCR in all strains (*Hrr. lacusprofundi, Haloferax mediterranei* and *Haloferax volcanii* at 17 hpi; *Halobacterium salinarum* and *Haloarcula japonica*, at 16 hpi; *Haloquadratum walsbyi* and *Haloterrigena turkmenica* at 8 hpi) (Fig. 6D–I, Suppl. Fig. 8). Only minor or no effects of the infection on growth were detected for all tested strains (Fig. 6A–C, Suppl. Fig. 8). While most strains exhibited higher cell-associated genome copy numbers of Tebi-PV1 (Suppl. Fig. 9), both viruses were detected in all strains following the initial adsorption period. The ratios of Tebi-PV1 to Tebi-SV1 genomes varied across the different strains, from ~5 in *Hbt. salinarium* to ~210 in *Hqt. walsbyi* (Suppl. Fig. 9), indicating different efficiencies of association with cells for the two viruses across the different strains. Comparison of total virus genome copy numbers in infected cultures with the total number of virus genomes added for infection indicates replication of Tebi-SV1 in *H. japonica* and *Hfx. volcanii* and replication of Tebi-PV1 in *Hfx. mediteranei* and *Haa. japonica* (Suppl. Data file 1: Suppl. Table 5, Suppl. text).

We transferred five of the cultured strains into fresh media, diluted to an OD_600nm_ of ~0.05, and continued monitoring growth (Fig. 6A–C, Suppl. Fig. 8). In all strains, the cell-associated virus number decreased over the monitoring timeframe, becoming undetectable in *Hfx. volcanii* (209 hpi). Extracellular virus numbers increased for Tebi-SV1 in *Hrr. lacusprofundi* and for Tebi-PV1 in *Hrr. lacusprofundi*, *Hbt. salinarium* and *Hfx. volcanii* (Suppl. Table 5, Suppl. text), indicative of virus replication and virus particle production. We determined the ratios of cell-associated virus genomes to host genomes for three of the strains (Fig. 6D–I). In all three, the viral genome copy number remained below the host chromosome copy number and decreased over time, indicating that not all cells were infected, though haloarchaea can contain several copies of their chromosome per cell^59,60^.

### Infectious virus particles can be isolated from lysed cells

**Fig. 7:**
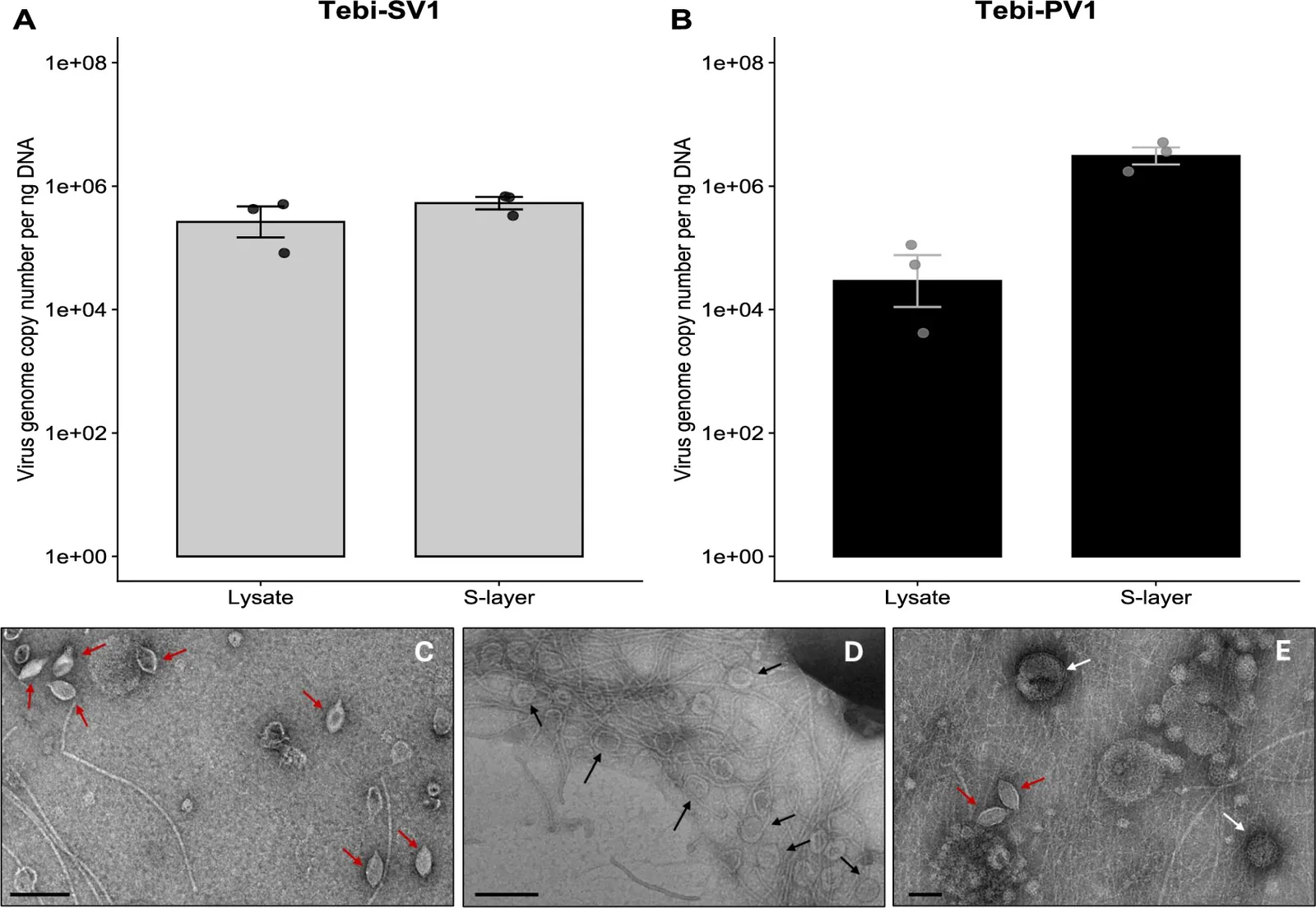
Isolation of virus particles from artificial cellular lysates. Viral genome copy number per ng of DNA is shown from the S-layer fraction and spheroplast lysate fraction for Tebi-SV1 (**A**) and Tebi-PV1 (**B**). Bars represent the mean across biological triplicates, error bars represent the standard deviation of *n* = 3 biological replicates (single data points). Negatively stained electron micrographs of the S-layer fraction (**C**, **D**) with spindle-shaped virus particles indicated with red arrows and pleomorphic virus particles indicated with black arrows (scale bar 200 nm). **E** Electron micrograph of spheroplast lysate preparation with spindle-shaped virus particles indicated with red arrows EVs indicated by white arrows (scale bar 100 nm).

Despite various induction efforts, we routinely detected Tebi-SV1 only in low amounts in culture supernatants. We hypothesized that virus particles accumulate within the cell, or at the cell surface^61,62^, without being released. Therefore, we manually lysed the host cells and indeed retrieved Tebi-SV1 particles from the artificial cellular lysate (Suppl. Fig. 12). Analysis of the genome copy numbers by qPCR revealed that both Tebi-SV1 and Tebi-PV1 genomes are present in these preparations, even after DNase treatment, indicating that both genomes are enclosed in particles or associated with structures protecting them from degradation. Additionally, virus particles obtained from the artificial lysate preparations resulted in infections of *Hrr. lacusprofundi* (Fig. 7). Notably, the Tebi-SV1:Tebi-PV1 ratio was significantly different in these preparations, with an average of 1:10 (in contrast to an average of 1: 1760 in culture supernatants), and we used these ‘Tebi-SV1 enriched’ preparations in downstream analyses.

To test whether infectious particles are bound to the S-layer on the cell surface of *Hrr*. TLS-6 cells, we first removed the S-layer from the cells by depleting the cells of MgCl_2_^63^, before lysing the spheroplasts by sonication. Approximately 50% of Tebi-SV1 protected genomes were detected in the S-layer preparations, and nearly equivalent amounts were also detected in the spheroplast lysate preparations. Higher titers of Tebi-PV1 genomes were detected in the isolated S-layer preparation than in the spheroplast lysate preparation (Fig. 7). With TEM, we observed numerous free spindle-shaped virus particles (Tebi-SV1) in the S-layer and spheroplast lysate preparations, while Tebi-PV1 virions were continuously observed attached to cellular pili-like structures (Fig. 7). The manually lysed preparations treated with protease showed no significant change in infectivity for Tebi-PV1 particles, but showed a significant reduction in infectivity of Tebi-SV1 particles, as observed for particles isolated from the supernatant (Fig. 7). Thus, we successfully isolated, infectious Tebi-SV1 and Tebi-PV1 particles from cells by removing the S-layer and manually lysing remaining spheroplasts.

## Discussion

Here we describe the discovery of two active viruses hidden in a haloarchaeal strain, *Halorubrum* TLS-6, isolated from a hypersaline lagoon in the Chilean central Andes^37^. When growing this strain from a single colony, we observed strong growth retardation and detected a spindle-shaped virus in the culture supernatant. Sequencing analyses revealed a second virus, a pleolipovirus. We propose the names Tebi-SV1 (Tebenquiche spindle-shaped virus 1) and Tebi-PV1 (Tebenquiche pleolipovirus 1).

Tebi-SV1 lacks sequence similarity with the previously isolated spindle-shaped virus His1 from a hypersaline environment and with any other characterized viruses or published virus genomes, highlighting that only its isolation allowed identification of this genetic element as a virus. We propose herein the virus family, *Almyrocitroviridae*, encompassing Tebi-SV1, a predicted provirus in *Halorubrum* strain LN27 (SSV-LN27), a proposed plasmid (IMGPR_plasmid_2904944409_000002)^50^, which likely represents a virus, and a group of recently discovered spindle-shaped viruses^61^. Tebi-SV1 and SSV-LN27 belong to the same genus based on sequence similarity (82% ANI).

Tebi-SV1 exists as a provirus within the genome of *Hrr*. TLS-6 and contains two integrases, VP1 and VP13, both belonging to the superfamily of tyrosine recombinases. Integrases of this family have been described for both pleolipoviruses^64^ and fuselloviruses (a family of spindle-shaped viruses)^65,66^. We identified the viral attachment site (14 bp) within a host tRNA (Fig. 4A). Tebi-SV1 encodes for a Cdc6-like protein (VP10). A recent study determined that an Orc1/Cdc6 protein (ORF8), along with a hypothetical protein (ORF7) and a winged helix-turn-helix DNA binding protein (ORF4), control the lysogeny-induction switch of pleolipovirus, SNJ2^56^. Structural alignments of the predicted structures of Tebi-SV1 VP9 with SNJ2 ORF7 (z-score 6.8, rmsd 3.1) and Tebi-SV1 VP8 with SNJ2 ORF4 (z-score 12.1, rmsd 2.1), suggest that VP8, VP9 and VP10 could represent the SNJ2 ORF4/ORF8/ORF7 system. We also identified a repressor binding site located between the promotor of the repressor and the promotor of the integrase operon, similar to the SNJ2 regulatory region. SNJ2 also exhibits a 14 bp attP and integrates into a host tRNA^35^. We, therefore, suggest that both the integration mechanisms and the mechanism controlling the lysis-lysogeny decision of Tebi-SV1 are similar to those of SNJ2 (Fig. 4). Whether the second integrase VP13 represents a paralog, has been derived through recombination with another MGE,^67,68^ or exhibits an alternative function such as Int1 of PhiCh1^69^ remains to be elucidated.

We detected six Tebi-SV1 proteins in virus particle preparations using proteomics (Suppl. text). Based on the predicted structural characteristics of the single subunit of protein VP35, which was detected in viral preparations, and the transmembrane helices profile similarities it shares with the major capsid proteins (MCPs) of other spindle-shaped viruses, we suggest that this could be the MCP of Tebi-SV1. We detected VP35 in size fractions larger than its predicted size (~17.5 kDa), suggesting post-translational modification (lipid modification) or the formation of strong multimers. Post-translational modifications may hinder detection by proteomics^43^, which could explain why it was not the most abundant protein in virus preparations.

The complete life cycle of Tebi-SV1 remains obscure (see Suppl. text). With the current data, we cannot infer whether Tebi-SV1 exhibits a chronic life cycle, as the majority of spindle-shaped archaeal viruses^34,62,70–73^, or whether Tebi-SV1 particles are released by cell lysis.

Tebi-PV1 exhibits the characteristic core genes shared by all members of the *Pleolipoviridae* family, as well as the commonly shared genes among the *Betapleolipovirus* genus^46^. Using proteomics (Suppl. text), we detected the spike protein and other structural proteins previously reported in pleolipovirus particles^46,74^. Interestingly, we also detected VP6, an archaeal type-IV pilin-like (T4P-like) protein, in virus preparations. The same preparation contained host pilin and archaellum proteins, suggesting that cell appendages co-purify with the viruses and that VP6 could be involved in forming pilin structures at the host cell surface, as recently described for the pleolipovirus SNJ2^36^. This result was complemented by TEM images of Tebi-PV1 virions closely associated with host cell appendages (Suppl. Fig. 13). These results together indicate that Tebi-PV1 may use pili for host cell attachment, which has been reported in several other archaeal viruses^14,75–77^. Influence of proviruses on host cell appendages was also shown for proviruses in *Hfx. volcanii*. Deletion of proviruses (including two pleolipoviruses) led to an increased expression of motility-related genes, including pili genes, and a significant increase in motility^6^. We conclude that some pleolipoviruses can target host pilin formation, both by changing the composition of the pili and by downregulating pili formation. These mechanisms likely evolved to prevent infection with the same virus or with other viruses; however, T4P modification by a pleolipovirus has also been shown to promote infection with a co-infecting virus^36^.

Virus preparations from TLS-6 were usually highly dominated by Tebi-PV1 (average genome copy number ratio 1760 Tebi-PV1:1 Tebi-SV1). Changing of growth conditions and attempts to induce Tebi-SV1, caused only minor changes to this ratio, except in CDM media supplemented with phosphate, where the difference is significant (average ratio ~80:1) (Suppl. Table 3). Therefore, we assume that Tebi-PV1 is constantly replicating, though Tebi-PV1 can integrate into a host isoleucine-tRNA, while the excision of Tebi-SV1 is tightly regulated, leading to an overrepresentation of Tebi-PV1 in virus preparations.

With the aim of finding a host strain that allows the propagation of only one of the two viruses, and a virus free host for both viruses, we tested seven different species of haloarchaea (representing six different genera) for their susceptibility to Tebi-SV1 and Tebi-PV1 infection. While none of the infected strains showed any significant growth defects, we could detect both viruses associated with cells and in culture supernatants across all tested haloarchaea by qPCR. Varying Tebi-PV1:Tebi-SV1 genome ratios in the different strains indicated different efficiencies of each virus to associate with the different species. Tebi-SV1 showed the highest efficiency with *Hbt. salinarium*, while Tebi-PV1 was more successful on all other tested strains. Therefore, we conclude that both viruses can attach to a wide range of haloarchaea (across genus level). Virus-to-host genome ratios were often far below 1 for the strains tested, which is likely due to the low virus-to-host genome ratios used for infection, and long-term cultures revealed the gradual decrease of both Tebi-SV1 and Tebi-PV1 genomes. Viral genomes detected in culture supernatants indicate replication and release of Tebi-SV1 in *Hrr. lacusprofundi* and *Hfx. volcanii* and Tebi-PV1 in *Hrr. lacusprofundi*, *Hbt. salinarium* and *Hfx. volcanii*. However, Tebi-SV1 was excluded from *Hfx. volcanii* during the second growth cycle (Fig. 6), and both viruses were excluded during subsequent transfers from the other strains (data not shown). It is typical for pleolipoviruses of the family *Pleolipoviridae* and spindle-shaped viruses of families *Thaspiviridae, Fuselloviridae*, and *Halspiviridae* to exhibit a narrow host range, with a few exceptions^40,62,78^. We show that Tebi-SV1 and Tebi-PV1 can associate with cells across six haloarchaeal genera, suggesting that both viruses use a receptor that is highly conserved across genera. Whether both viruses use the same receptor needs to be determined.

While virus preparations from *Hrr*. TLS-6 always contained both Tebi-SV1 and Tebi-PV1 particles; there was a clear tendency that the abundance of one virus changed with the abundance of the other virus. The first virus preparation from *Hrr*. TLS-6 had a ratio of 1200 Tebi-SV1 genomes per Tebi-PV1 genome, while in any following preparations this ratio was inverted, with 1760 Tebi-PV1 per Tebi-SV1 genome. When attempting to induce a higher production of Tebi-SV1 particles, an increase in Tebi-SV1 abundance also correlated with a decrease in Tebi-PV1 abundance (e.g., CDM medium versus CDM + PO4 and DBCM2 media versus CDM media, Suppl. Fig. 4). We concluded that the activity of the viruses is at least partially regulated by virus-virus interactions.

We could not identify growth conditions that would reproduce the high titers of Tebi-SV1 particles obtained in the first virus preparation of *Hrr*. TLS-6, however, we were able to identify a genomic variation of Tebi-SV1 particles produced from this culture. Sequencing of the virus genome from the respective culture revealed two SNPs in the Tebi-SV1 genome that are located in the repressor binding site between VP8 and the integrase operon (Fig. 4B). The repressor binding site of the SNJ2 virus was shown to regulate expression of the integrase operon^64^. Therefore, we conclude that the mutation of the operator led to the increased activity of Tebi-SV1 in this particular culture. Interestingly, the sequence of the operator of Tebi-PV1 is identical to the operator of Tebi-SV1 (Fig. 4B). We propose that the Tebi-PV1 repressor can bind the Tebi-SV1 operator. While the operator identified in Tebi-SV1 might be longer, the Tebi-SV1 repressor could potentially also bind the Tebi-PV1 operator; however, this remains to be experimentally verified. We also identified a 5’ UTR upstream of the integrase operon that is conserved across Tebi-SV1, Tebi-PV1, LN27-SSV and SNJ2. We propose that the 5’ UTR has a regulatory function for the integrase operon, which would represent an additional conserved regulatory element controlling the lytic-lysogeny decision. Tebi-SV1 is integrated into the host genome and is usually represented in significantly lower numbers than Tebi-PV1, with the exception of the viral preparation that exhibited mutations in the operator, suggesting that Tebi-PV1 VP4 may reduce Tebi-SV1 extrachromosomal genome copy numbers. The exact target and mechanism of this regulation needs experimental validation. Similar virus-virus regulatory mechanisms have been observed in other archaeal systems. In *Hfx. volcanii*, for example, the provirus Halfvol3 and virus HFPV-1 were both shown to influence each other’s transcription and replication. HFPV-1 massively downregulated the integrase and a transcriptional regulator (similar to PhiH-like repressors) in the Halfvol3 provirus region upon infection, and in a Halfvol3-free mutant, HFPV-1 reached significantly higher genome copy numbers^11^. In *Halobacterium halobium* cells, a homolog to PhiH1 repressor protein gives the cells immunity to PhiH1 virus^79,80^.

The proposed regulation between Tebi-SV1 and Tebi-PV1 does not seem to represent a strict superinfection exclusion mechanism as previously described for virus-virus coinfections of archaea, where one virus completely prevents infection of the other^17,35,81^. Instead, our data suggests a more subtle form of mutual regulation, allowing both viruses to co-exist while modulating each other’s activity. We found that four out of six host genomes containing Tebi-SV1-like viruses also harboured pleolipovirus-related sequences (Suppl. text). While this could be coincidental, it still raises the question whether maintenance and replication of certain halophilic spindle-shaped viruses may depend on coinfection with a compatible pleolipovirus. Similar to the recent discovery of the mutual interaction between SNJ1 and SNJ2^36^, with SNJ2 providing the receptor for SNJ1 while SNJ1 supports the replication of SNJ2, Tebi-SV1 and Tebi-PV1 may be dependent on each other. Infection of other haloarchaea with the mixture of the two viruses always led to a coinfection with both viruses, suggesting that a coinfection could be crucial for both viruses to enter new host cells.

## Conclusion

In this study, we isolated and characterized a spindle-shaped virus (Tebi-SV1) that co-exists with a pleolipovirus in the same host strain. We were only able to identify the spindle-shaped virus (Tebi-SV1) because we visualized the culture supernatant with TEM. Relying solely on genome analysis would not have allowed for the identification of this MGE as an actual virus, due to the structure and sequence of the virus genome. This clearly demonstrates that isolation is often still a crucial method for identifying viruses, especially when working with archaeal viruses. We uncovered that the coinfecting pleolipovirus, Tebi-PV1, might regulate the activity of Tebi-SV1 through a repressor protein, without excluding Tebi-SV1 from the host. Additionally, Tebi-PV1 encodes for a T4P, which we detected by proteomics, that may also play an important role in both virus-host and virus-virus interactions. Finally, coinfection with both viruses seems to be crucial to allow for the infection of other haloarchaea. Considering other recent findings on the interactions of pleolipoviruses with coinfecting viruses and their prevalence in haloarchaea genomes, we propose that pleolipoviruses play a particularly important role in virus-host and virus-virus interactions in haloarchaea. This mutual regulation portrays the importance of understanding such complex virus-virus interactions that may occur across all domains of life.

## Experimental Procedures

### Strains and cultivation conditions

All haloarchaeal strains used in this study are summarized in Suppl. Table 9. *Halorubrum sp*. strain TLS-6 (TLS-6) was isolated from a microbial mat from Laguna Tebenquiche, Chile^37^. TLS-6 was cultivated either in nutrient rich media, modified DBCM2^82^, or alternatively, nutrient deficient media, modified CDM^83^ supplemented with 1 mM phosphate (K_2_HPO_4_). Additional media that was used (Suppl. Fig. 1) included DBCM2+ Mod (DBCM2 supplemented with 7.5 g/L Peptone and 1.5 g/L Yeast Extract) and WJK media as described previously^84^. All *Halorubrum* strains were cultured aerobically with constant agitation of 120 rpm at 28 °C unless otherwise stated. All strains were inoculated from glycerol stocks (in media with 15% glycerol stored at −80 °C) and grown in approximately 5 mL of the corresponding media and temperatures (Suppl. Table 10) until an optical density at 600 nm (OD_600_) of ~0.5. The cultures were diluted to OD_600_ = 0.05 in fresh media and regrown to an OD_600_ of approximately 1. Dilution to OD_600_ = 0.05 was repeated once more in fresh media and cultures were grown to exponential growth phase before used for all experimental procedures described. Conditions tested to enrich either Tebi-PV1 or Tebi-SV1 included UV treatment, antibiotics, and PO_4_ supplementation (1 mM K_2_HPO_4_). UV treatment was performed in early exponential growth phase using a UV crosslinker from Biometra™ at 0.05 J of UV radiation for 30 s. Antibiotics Mitomycin C (15 µg/mL) and Pravastatin (1 µg/mL) were added in early exponential phase.

### Isolation and purification of viruses

*Hrr*. TLS-6 was initially cultivated from a single colony (CDM agar) in 50 mL CDM^83^ supplemented with 1 mM K_2_HPO_4_ and with and without 11 µM arsenic (As (V)). The cultures did not reach exponential phase, but were, regardless, diluted into 500 mL and grown for ~30 days. Cells were removed by filtration with 0.45 µm pore size followed by 0.22 µm pore size filters. Subsequently, the flow through was ultra-centrifuged (41,000 rpm for 2 h, 4 °C, fixed angle 70.1 Ti rotor, Beckman & Coulter). The resulting pellets were re-suspended in DBCM2 salt solution (180 g/L NaCl, 25 g/L MgCl_2_, 29 g/L MgSO_4_·7 H_2_O, 5.8 g/L KCl). For genomic DNA extraction, the preparation was syringe filtered (0.22 µm pore size) and treated with 50 µg/mL RNase A (16 units, ThermoFisher™) and 50 µg/mL DNase I (16 units, NEB) at 37 °C for 2 h. The virus preparation was then loaded onto an Iodixanol (25–40% OptiPrep) gradient that was pre-layered and allowed to linearize overnight (38,000 rpm for 20 h, 4 °C, SW41 Ti Swinging-Bucket rotor, Beckman & Coulter). Three resulting bands were extracted with a syringe, and virus-like particles (VLPs) were observed under the TEM. DNA was extracted from all bands (see Preparation and analysis of viral genomes), and DNA from the second band was used for genome sequencing.

For Tebi-SV1 enriched preparations, TLS-6 was grown in 1 L of modified CDM supplemented with 1 mM phosphate. Cells were collected *via* centrifugation (4500 x g, 45 min) and resuspended in 6 mL of DBCM2 salt solution. Cells were harvested *via* centrifugation (10,000 x g, 10 min) and washed once more with fresh buffer to remove any free virus particles. After centrifugation (10,000 x g, 10 min), the cells were resuspended and incubated in spheroplasting solution (1 M NaCl, 27 mM KCl, 50 mM Tris-HCl, 15% sucrose) and 0.5 M EDTA, pH 8, at room temperature (RT) for 10 min. Samples were sonicated in an ice water bath (Bandelin Sonorex TK52) for 25 min. Cell debris was removed by centrifugation (15,800 x g, 30 min at 4 °C) and filtration with 0.45 µm pore size followed by 0.22 µm pore size filters. The artificial lysate was collected by ultracentrifugation (41,000 rpm for 2 h, 4 °C in Beckman 70.1 Ti rotor), the pellet was dissolved in 1 mL DBCM2 salt solution, and then sterile filtered once more with 0.22 µm pore size. For Tebi-PV enriched preparations, TLS-6 was grown in nutrient rich media (DBCM2). Cultures were harvested in stationary phase by centrifugation (4500 x g, 45 min, 4 °C) to pellet the cells. The supernatant was filtered (0.22 µm pore size), and virus particles were collected by ultracentrifugation (as above). The re-suspended (1 ml DBCM2 salt solution) pellets were filtered once more (0.22 µm pore size) to a final volume of 1 mL. DNA extraction was performed on 20 µL of DNase I treated concentrated supernatant and screened for both viruses by PCR or qPCR (see *Virus quantification by qPCR*). Virus preparations were tested for host contamination both by PCR and by incubation in medium for several days before further use.

### Genome sequencing and genome analysis

DNA was extracted using a genomic DNA extraction kit (ISOLATE II PCR & Gel Kit, Bioline®). Library preparation (FS DNA Library, NEBNext® Ultra™) and sequencing Illumina HiSeq3000, 2 × 150 bp, 1 Gigabase were performed at the Max Planck-Genome-Centre (Cologne, Germany). The reads were quality trimmed using Cutadapt^85^ with a minimum quality of 30, and a minimum length of 50 (-q 30, -m 50). Assembler metaplasmidSPAdes^86^ was used to perform assembly of high-quality trimmed reads. Sequence similarity was assessed using BLASTn^87^, e-value < 10^−^^5^, word size 28, and compared to public databases NCBI, Nucleotide Selection, and RefSeqProteins^88^. Open reading frames were predicted using Prodigal^89^. InterProScan^90^ was used to detect putative conserved domains and other protein signatures. Manual annotations of the two resulting contigs were compared and refined by IMG/VR^91^ NCBI BLASTx, InterProScan, HHblits^87,90,92^, Uniclust^93^, and Glimmer^94^ results. Additionally, tertiary structures were predicted with Alphafold 3^95^ and searched against PDB with Dali^96^. The Mauve alignment algorithm and EasyFig BLAST tool were used to identify similar viral and host genomes for whole-genome alignments^97^. Accession numbers for the genomes of Tebi-SV1 and Tebi-PV1 are PX315771 and PX315772, respectively. The *Halorubrum* TLS-6 genome sequencing reads (SRR12122876, SRA) were assembled with SPAdes v.3.11.0 (default parameters) into 48 contigs (~1.6 Mbp, ~1.2 Mbp, ~101 kbp, 15.2 kbp, 2.2 kbp, 1.8 kbp,1.5 kbp, and 41 contigs below 1 kbp)^98^. Resequencing of the TLS-6 genome was performed as described above. To identify mutations in the Tebi-SV1 and Tebi-PV1 genomes, the reads (available under BioProject accession number PRJNA1322139) were trimmed and mapped to the assembled genome of TLS-6 using Geneious mapper (Geneious Prime® 2025.0.3, low sensitivity, default parameters), and the ‘find variations’ feature (default settings).

### Phylogenetic analysis and identification of relatives

Network analyses were performed using vConTACT2^99^ with the Viral RefSeq-archaea database (version 211) and the IMGPR database^100^. The analysis utilized BLASTP as the relationship mode (—rel-mode BLASTP) with an e-value threshold of 10⁻³ to determine significant sequence similarities. The analysis revealed the close relationship of proposed plasmid IMGPR_plasmid_2904944409_000002 with Tebi-SV1. The resulting network structures were visualized using the R packages ggplot2 and Network^101,102^, allowing for the graphical representation of gene-sharing patterns. Phylogenetic analysis and whole-genome comparisons were conducted using VICTOR^103^, considering D0, D4, and D6 formulas of predicted gene sequences for clustering and taxonomic assignment. Clustering results were reconciled with Victor analysis to indicate the acceptable thresholds of gene-sharing at the approximate genus level. The average nucleotide identity (ANI) threshold (95%) used to delineate species in viral genomics was taken from ref. ^52^.

### Protein content analyses of virus particles

Purified virus preparations were supplemented with 1X sample buffer (0.2 g SDS, 1 mg Bromphenol Blue, 0.78 mL glycerol, 0.2 mL 0.5 M Tris pH 6.8, and 0.155 g DTT per 10 mL) and Urea (3.3 M). The protein mixture was heat denatured for ~10 min at 95 °C. Viral proteins were separated by Tris-Glycine sodium dodecyl sulfate polyacrylamide gel electrophoresis (SDS PAGE) gel electrophoresis (12% acrylamide in resolving gel). Gels were stained with fast Coomassie staining (50% ethanol, 10% acetic acid, 0.5% wt/vol Coomassie Blue) (Suppl. Fig. 10). In-gel digestion of excised bands or lanes (as stated in Suppl. Fig. 10 and Suppl. Data file 1: Suppl. Table 7) was performed as described in ref. ^104^. Protein content analyses of gel separated samples was performed using mass spectrometry (MS) as described in ref. ^105^. For protein identification, we generated a custom database by predicting open reading frames from the host genome (Prodigal^89^) and manually curating the viral genome as described in *Viral genome sequencing and genome analysis*. Additionally, we included protein sequences predicted by the cRAP protein sequence database (http://www.thegpm.org/crap) of common laboratory contaminants. Searches of the MS/MS spectra against this database were performed with the Sequest HT node in Proteome Discoverer vs. 2.5 (Thermo Fisher Scientific). False discovery rates (FDRs) for peptide spectral matches (PSMs) were calculated and filtered using the Percolator Node in Proteome Discoverer. The percolator was run with a maximum delta Cn 0.05, a strict target FDR of 0.01, and a relaxed target FDR of 0.05, and validation based on q-value. The Protein FDR Validator Node in Proteome Discoverer was used to calculate q-values for inferred proteins based on the results from a search against a target-decoy database. Proteins with a *q*-value of < 0.01 were categorized as high-confidence identifications and proteins with a *q*-value of < 0.01 were categorized as medium-confidence identifications.

### Chloroform and Protease treatment of viral preparations

Triplicates of cell lysate preparation or supernatant virus preparation from TLS-6 cultured in CDM + 1 mM K_2_HPO_4_ (see *Isolation and purification of viruses*) were diluted with 2 parts of water to preserve the activity of Proteinase K. A final concentration of 7.5 µg/ml of Proteinase K, or the equivalent volume of H_2_O were added and samples were incubated for 4 h at 37 °C with occasional inversion to mix. The proteinase K was inactivated with PMSF (2 mM final concentration). Unprotected DNA was digested with DNase I after adding 1x final concentration DNase buffer for 2 h at 37 °C (New England Biolabs). The salinity was adjusted with DBCM2 salt solution, and the entire sample was used to infect *Hrr. lacusprofundi* cells (~5E + 05 cells). Fresh media was added to reach a final volume of 1.5 mL, and samples were left to adsorb for ~16 h at room temperature. Cells were then harvested at 4500 x g for 45 min and the cell pellets were washed twice with DBCM2 salt solution (10,000 x g for 10 min). DNA was extracted from cell pellets, and virus genomes associated with cells after washing were detected *via* qPCR.

Triplicates of cell lysate preparation or supernatant virus preparation from TLS-6 cultured in CDM + 1 mM K_2_HPO_4_ (see *Isolation and purification of viruses*) were exposed to chloroform (manufacturer) in a 1:4 (solvent to virus) volume ratio and incubated with constant agitation for 20 min at RT. The samples were centrifuged (13,500 x g, 3 min) and the watery upper layer was collected in a fresh tube. For untreated controls, the same volume of the same virus preparation was used; however, no chloroform was added. Unprotected DNA was digested with DNase I after adding 1x final concentration DNase buffer (2 h at 37 °C). An equal volume of fresh DBCM2+ media was added to the sample (final volume ~1.5 mL). *Hrr. lacusprofundi* cells were infected (as above) with chloroform treated and control virus preparations. Infected cells were harvested, washed, and screened for internalized virus genomes with qPCR (as above).

### Infectivity and host range assessment

Seven different haloarchaeal hosts were tested in biological triplicates (Fig. 6 & Suppl. Fig 8). All strains were grown in 2 sequential cultures as described above, and infected in the exponential phase with a total of approximately 2.5 virus genome copy number (0.09 for Tebi-SV1 and 2.5 for Tebi-PV1; total virus genome copy number added per culture: 1.16E + 08 Tebi-SV1 and 3.34E + 09 Tebi-PV1) per host cell. The viral preparation derived from concentrated cell lysate that was purified from remaining host cells and cell debris by centrifugation (15,800 x g, 30 min at 4 °C) and filtration with 0.45 µm pore size followed by 0.22 µm pore size filters. Host cell number was calculated with 1.65E + 9 in 7 mL at OD600 0.05, using cell numbers determined for *Hrr. lacusprofundi*^27^. Notably, these numbers can differ slightly for the other organisms. After incubation overnight at RT (17 hours for *Hrr. lacusprofundi, Haloferax mediterranei* and *Haloferax volcanii*; 16 h for *Halobacterium salinarum* and *Haloarcula japonica*; 8 hours for *Haloquadratum walsbyi* and *Haloterrigena turkmenica*) for adsorption, cells were pelleted and washed twice to remove any unbound virus remaining in the supernatant (10,000 x g, 10 min and re-suspended in 500 µL medium). Cells were transferred into 7 mL fresh medium and incubated as described. Optical density at 600 nm (OD_600nm_) was used to monitor growth kinetics (Fig. 6 and Suppl. Fig. 8). Samples (1.5 mL) were taken at different time points post infection (indicated in the figures), where hours post-infection (hpi) specify the time after the virus was mixed with host cells for adsorption. Cells from 1.5 mL of culture were harvested by centrifugation (10,000 x g, 10 min), washed twice with fresh media (to remove any free viruses), and screened for infection with Tebi-SV1 and Tebi-PV1 with specific qPCR probes (Suppl. Table 10). The supernatant was filtered twice with 0.22 µm syringe filters, and virus particles were harvested by ultracentrifugation (41,000 rpm for 2 h, 4 °C, fixed angle 70.1 Ti rotor, Beckman & Coulter). The pellets were resuspended in 100 µL of DBCM2 salt solution and treated with DNase I before DNA extraction and quantification by qPCR. Total numbers of virus at time point 2 (Supplementary Data file 1: Suppl. Table 5) were determined by multiplying the genome copy number/ng DNA in 1.5 mL culture with the total DNA extracted from 1.5 mL; and the culture volume of 7 mL was included by multiplication of results from 1.5 mL with 4.6.

### Virus quantification by qPCR

DNA extractions were performed with an Isolate II Genomic DNA Kit (Bioline, London. UK) according to the manufacturer’s instructions. DNA concentrations were measured using a Spectrophotometer (DS-11 FX + , DeNovix®). In brief, the qPCR program consisted of the following: 5 min at 95 °C and 30 s at 95 °C, 30 s at X °C for 35–40 cycles (X, corresponding to the annealing temperature of the respective primer sets, is indicated in Suppl. Table 10) using a CFX96 Touch Real-Time PCR (Bio-Rad Laboratories, Inc., Hercules, CA, United States) and the software CFX Manager™ Software. Each reaction (10 μL) contained 1X SsoAdvanced Universal SYBR™ Green Supermix (Bio-Rad) and primer concentrations as stated in Suppl. Table 10. Dimer formation was checked using OligoAnalyser (Inegrated DNA Technologies). All primer pairs were mapped to the relevant genome with a 5 bp mismatch allowance to ensure no unspecific binding would occur. All qPCR data had an efficiency between 90-110% with an R² value of > 0.95. Host and virus genome copy numbers were determined in technical triplicate for each biological replicate as described previously^27,106^. The primer sequences, annealing temperatures, and concentrations of all primers used are listed in Suppl. Table 10. Statistical analyses were performed using two-sided t-tests. Equality of variances was assessed using an F-test. Student’s t-tests assuming equal variances were used when the F-test was not significant (*p* ≥ 0.05), whereas Welch’s t-tests were used when the F-test indicated unequal variances (*p* < 0.05).

### Negative-stain Transmission electron microscopy

Five µL of a viral preparation was adsorbed onto a carbon-coated copper grid for 3 minutes. The sample was dipped into a 5 µL droplet of ddH_2_O before negative staining with 2% uranyl acetate for 45 s. Imaging was performed at 200 kV using a JEOL JEM-2100 Plus electron microscope.

## Statistics and Reproducibility

Data for all experiments, with two exceptions, were collected from at least three biological replicates. One exception was the culture from which Tebi-SV1 was initially isolated in high copy numbers, which could not be reproduced. The culture was grown from a single colony that exhibited a spontaneous mutation. The second exception is a growth curve presented in supplementary material (Suppl. Fig. 1), which only represents data from a single replicate for each condition. Data are expressed as means ± standard deviation in box plots. Statistical analyses were performed using two-sided t-tests. Equality of variances was assessed using an F-test. Student’s t-tests assuming equal variances were used when the F-test was not significant (*p* ≥ 0.05), whereas Welch’s t-tests were used when the F-test indicated unequal variances (*p* < 0.05).

### Reporting summary

Further information on research design is available in the Nature Portfolio Reporting Summary linked to this article.

## Supplementary information

Transparent Peer Review file

Supplementary Information

Description of Additional Supplementary Files

Supplementary Data 1

Supplementary Data 2

Reporting Summary

## Data Availability

The genomes of Tebi-SV1 and Tebi-PV1 are available under accession numbers PX315771 and PX315772, respectively. Raw reads for the re-sequencing of the TLS-6 genome are available under BioProject accession number PRJNA1322139. The mass spectrometry proteomics data have been deposited to the ProteomeXchange Consortium via the PRIDE partner repository^107^ with the dataset identifier PXD068819. The source data for Figs. 5, 6, and 7 and Suppl. Figs. 1, 4, 8, 9 and 12 are in Suppl. Data 2.
