# Supplementary material for: Two coinfecting archaeal viruses provide insights into virus-virus interactions
Source: Commun Biol. 2026 Sep 24;9:1250. doi: 10.1038/s42003-026-11061-7 (PMC13612827; doi:10.1038/s42003-026-11061-7)
Supplement: Supplementary file 1 [file 42003_2026_11061_MOESM1_ESM.pdf]

nature portfolio

Peer Review File

Two coinfecting archaeal viruses provide insights into virus-virus interactions

Corresponding Author: Professor Susanne Erdmann

This file contains all reviewer reports in order by version, followed by all author rebuttals in order by version.

Version 0:

Reviewer comments:

Reviewer #1

(Remarks to the Author)
Review of NCOMMS-25-70889-T

Key results:
Authors discovered a fusiform virus-like particle (Tebi-SV1) in a Halorubrum strain but were unable to induce production of the particle. On analysis of DNA isolated from the culture, a second putative viral genome was found that was similar in sequence to known pleiomorphic halophilic viruses. The apparent genome of the larger particle is similar to genomes found by other researchers, either as plasmids or proviruses and is most convincingly similar to SSV_LN27. Interestingly, most of the putative coding sequences in Tebi-SV1 (and SSV-LN27) appear to be quite unique (BLAST e-value >10-5). The authors do not convincingly show virus replication in any other strains with the possible exception of Hrr. lacusprofundi. Putatively virus-encoded proteins are detected with mass spectrometry in cell-free extracts.

Validity:
I find the data presented to be valid and reasonable, but I find the data to be overinterpreted. (See comments below).

Significance:
I think that new viruses of haloarchaea, particularly spindle-shaped ones, to be very interesting. That the Tebi-SV1 genome has very few ORFs with clear similarity to other viruses is striking and very interesting. The emphasis in the manuscript, particularly in the title, abstract and discussion on interactions between different viruses infecting the same host is not supported by the data presented. I do not find that many insights into virus-virus interactions can be gleaned from the data presented. Moreover, data presented in Xiang et al (reference 35) on SNJ1 and SNJ2, two viruses that infect a halophilic archaeon and encode pili, is not sufficiently discussed.
Data and methodology:
Data presented are reasonable but do not sufficiently support the conclusions of the manuscript. The majority of the data presented are on characterization of each of the virus-like particles. Much of the data are based on qPCR experiments which are insufficiently described in the manuscript.

Analytical approach:
In general the analytical approach is appropriate, however evidence for viral replication (Figure 6) is not convincing.

Suggested improvements:

General: I strongly recommend that host range studies be presented much earlier in the manuscript.

Specific:
L106: What is typical growth behavior? Please provide a reference or description.
L108: Please clarify if this TEM analysis was directly on supernatant (as implied in the text), or concentrated (as stated in the figure legend).
L110: These are very nice spindle-shaped VLPs, but seem somewhat pleiomorphic a discussion or analysis of TEMs should be presented.
L115-117: Please state order of bands from top or bottom or label in Figure 1E.

L119: Please define scale bars in panels B, C and D. .
L119: Please clarify what is meant by “first virus preparation”?
L122: Please clarify from which sample these VLPs were isolated.
L125: Please specify source of “total DNA”, was this from band 2, supernatant or some other sample.
L132: Please clarify what is meant by “detected”.
L132: Please clarify the difference between the genome sequence of the host and what was sequenced here. Apparently the host genome was resequenced (L646). Please clarify what was done in the text.
L142: Please define “significant similarity” and “high similarity”.
L147-149: This section seems to repeat L132-134, Please clarify.
L150: Please clarify what is meant by “it remains to be elucidated if Tebi-PV1 also can integrate into the host chromosome” if a provirus with identical sequence is found in the host genome.
L153: “Functional annotation”? what does this mean?
L155: Please specify if amino acid identity is meant here.
L166: Virus families can only be named via the ICTV, please make an appropriate proposal and add “putative” or “proposed” here.
L169: Figure 3: Sequences used in this tree must be identified by accession numbers and those used better described. The description in the figure legend and methods is insufficient to regenerate these data. The legend must also describe the color coding and scale of bar.
L173: Gene sharing network analysis is not convincing. These data appear to show that Tebi-SV1 and SSV_LN27 are similar to each other, but very dissimilar to other sequences. I suggest removing this section (L173-182 and associated supplementary figures) or rewriting considerably.
L192: Please provide references for this putative integrase-mediated circularization, for instance from alphafusellovirus SSV1.
L195-199: While these differences between the genome sequence and that of the particles is interesting, it must be better described, particularly in terms of the confidence of the sequences, before SNPs can be confidently discussed.
L200-223, including Figure 4: These data are interesting, but the description could be greatly reduced and figure placed in supplementary data.
L202: Presumably this is band 2, but please specify.
L205: Please explain what is meant by “lysed cultures” and “reinfection”. There is no mention of lysed cultures previous to this in the manuscript.
L206: Please clarify how this genome copy number was determined, also based on sequencing coverage?
L213: Please specify how “maintenance” was determined, presumably with PCR.
L220: Please clarify what is meant by “different temperatures”.
L224-229: Details must be provided on how “removal” and “infectivity” was determined. I strongly recommend that the section on host range be moved to before this section.
L240-243 (Figure 5): Electron micrographs are unconvincing.
L247: Please specify if this was virion-containing supernatant or gradient-purified particles. Gradient purification could degrade infectivity.
L249: These data appear to be virus genomes associated with cells, not “intracellular”. Please clarify here and throughout the manuscript.
L254-257: This description can be removed, since the previous sentence is sufficient.
L257: Please provide how “significance” was determined.
L257: Please state clearly how “intracellular” and “extracellular” virion numbers were counted. In the methods, for extracellular particles, this appears to be a resuspended ultracentrifugation pellet that has been treated with DNAse. Relative quantification of this sample relative to washed cells is problematic and should be stated as such.
L280: Figure 6: For all tested hosts, with the exception of Hrr. Lacusprofundi, the number of virus genome copies is multiple orders of magnitude lower at the second time point. Thus, there is no indication that virus replication is taking place.
L383-Discussion.
I think that this section should be rewritten after critical analysis of results.
L391-: As stated above, please submit a taxonomic proposal to the ICTV and state that these are proposed in the text.
L408: Please provide a citation for the statement suggesting a lipid layer.
L409: Please provide details on how similar this protein is to HEPN toxin proteins. Without these data it is impossible to interpret the proposed functions.
L419: Please either show or describe these structural characteristics. Are they more than the 2 predicted transmembrane domains shown in Figure S8?
L461: See previous comments regarding infectivity. I am not convinced that these viruses are infecting other strains than Halorubrum.
L487-: If other strains are being infected with these viruses, it should be possible to determine whether infection is chronic or lytic. If this is not the case, then this section is speculative.
L524-: For all of the protein sequences discussed in this section (and the remainder of the discussion), please provide data on sequence similarity. This could possibly be in a (supplemental) table.
L536-538: This interesting result needs to be more clearly presented in the text.
L543: Please clarify what data are being referred to here.
L539-540: Please be precise about what is shared and what is not.
L539: Please clarify that VP8 is a predicted transcriptional regulator.
L620: This appears to be artificially lysed cells (spheroplast + sonication). Please be clear about how this was generated in the text. Moreover, if I understand correctly, particles from artificial lysis were concentrated with ultracentrifugation. This should also be clearly stated in the text.
L664: Please restate which preparations were used here, from the iodixanol gradient?

L695: This procedure does not guarantee that these viral genomes have entered cells, just that the virus genome is associated with cells after washing.
L709: Please clarify how MOI was determined, was this based on qPCR?
L715: Please provide data on how "optimal" was shown or delete.
L800: Typographical error in reference. "ulfolobus" should be "Sulfolobus"
L857: Suggest including bioRxiv reference here instead of doi.
Supplemental material:
L10: Details of how this figure was generated, particularly "shared protein content" should be shown. Genomes that are labeled should be described.
L34: Supplemental Figure 4: Figure legend seems to be missing for panels C and D. qPCR data appears to be missing from panels C and D.
L46: Supplemental Figure 5: Please be consistent in labels of Y axes. See comment to text line 257.
L105: Supplemental Figure 10: Please provide statistics for how often Tebi-PV particles are observed associated with putative cellular appendages.

Clarity and context
The manuscript is mostly clearly written, see suggested improvements above.

References
While only just published, the authors should carefully review and comment on Zhao et al., 2025 (https://www.nature.com/articles/s41564-025-02149-7) in terms of their results. A preprint of this manuscript was available in February 2025.

Your expertise
I consider myself to be very qualified in all of the methods described in this manuscript.

Reviewer #2

(Remarks to the Author)
The authors provide a compelling study of relevance to the archaeal community. Especially since characterizations of virus-virus interactions in archaea are still sparse, these findings would benefit future viral research. While the experimental approaches are solid and well documented the irreproducible outcome of the culture that was used to produce the data for Figure 1 stands in stark contrast to the later experimental work in which the ratio between the viruses is inversed. Additionally, some of the claims seem somewhat speculative and are not fully backed up by experimental data.
Comments to the Author:
A high Tebi-SV1 to Tebi-PV1 ratio of 1200:1 in the culture that was used for TEM imaging of Fig.1 compared to any follow up experiments with completely different average ratios of 1:1760, raises the question if these TEM images are representable for this viral system. Especially since the Tebi-SV1 genome of the first preparation had two SNPs in an apparent operator region. Since TEM imaging of other cultures has been performed i.e. Fig S9, I would strongly urge the authors to directly address this point early in the manuscript and possibly provide TEM images of a culture that reflects the (viral ratios of) rest of the study to alleviate any doubts about the findings shown in Fig. 1., or at least link to supplemental Images for comparison.
Regarding the hypothesis in Line 42-44 (and Line 524ff.): "Virus abundances seem to be regulated through a shared operator sequence allowing Tebi-PV1 to keep 44 Tebi-SV1 copy numbers low without excluding Tebi-SV1 from the host genome." While this claim could significantly elevate the impact of this study, unfortunately there is only limited experimental evidence suggesting this. The observation that a mutant Tebi-SV1 with a 2 bp variance in a shared homology region Tebi-PV1 shows a drastically increased relative abundance is indeed intriguing. But whether the observed phenotype is due to an altered auto- or co-regulation or unintended experimental variation still remains to be elucidated, since no evidence has been delivered that the region of interest is in fact an operator.
The low effectiveness of Proteinase K and chloroform treatment in deactivating the viral particles is surprising and should be addressed in the discussion, perhaps with references how this relates to other viruses.
Throughout the manuscript qPCR on washed cell pellets is being used to quantify viral DNA as an indication for successful infection. As washing would not remove adsorbed/surface attached viruses that have not (yet) completely fused with the host or injected their genome, it seems that this approach would manly quantify the adsorbed fraction. A clarification on this would be important, as adsorption without genome injection is a potential outcome with alternative hosts. Additionally, the interpretation of the data in Figure 6 would highly benefit if (at least semi-quantitative) statements could be made if or how much the total virus genome copy numbers changed throughout the experiment, i.e. how much did the virus(es) actually amplify?
Minor points:
Supplementary Table 3 has "Supplementary table 2" in its header

Line 355 the opening bracket "(" of (Supplementary Figure 9) is bold.
I think a minor editing error occurred in the legend of Fig. 1: Perhaps the mentioning of panels (B-C) slipped into the sentence before and was meant to be liked to "… . (B-C) Tebi-SV1 particles exhibiting pleomorphism with elongated tails and in a tubular form (size bars 122 are 100 nm) from band 3 of Iodixanol gradient (E)."
The software/tool that was used for the secondary structure search in order to improve the viral genome annotation appears to not be mentioned.

Reviewer #3

(Remarks to the Author)
I co-reviewed this manuscript with one of the reviewers who provided the listed reports. This is part of the Communications Biology initiative to facilitate training in peer review and to provide appropriate recognition for Early Career Researchers who co-review manuscripts.

Reviewer #4

(Remarks to the Author)
Queiss and colleagues report the isolation of two new viruses infecting an extremely halophilic archaeon, Halorubrum sp. TLS-6. One of the two viruses has a pleomorphic morphology and belong to the existing family Pleolipoviridae, whereas the second virus produces spindle-shaped virions and is a founding member of a novel virus family, which the authors tentatively named 'Almyrocitroviridae'. The two viruses are engaged in a complex relationship, and I would like to commend the authors for their efforts to unravel it in a non-model organism, such as Halorubrum sp. TLS-6. It is increasingly clear that virus-host interactions are more complex that usually considered and typically involve multiple coinfecting viruses. Thus, this work will be of great interest to the community of researchers working on archaea as well as those working on prokaryotic viruses. The manuscript is largely clearly written. However, some of the interpretations are overly speculative and should be tightened, as detailed below in my relatively minor comments.

L37,39: The capitalization in Tebenquiche spindle-shaped Virus 1 (Tebi-SV1) and Tebenquiche pleomorphic Virus 1 (Tebi-PV1) appears random. Either capitalize all words used for abbreviation or only the proper nouns. To this reviewer, removing capitalization from Virus would be the more appropriate solution.

L42: In the absence of evidence of actual mutualism between the two viruses, it might be more appropriate to state that the relationship is symbiotic.

L71-73: Interaction between satellite viruses and helper viruses is not cooperative. Satellite viruses are typically parasites of their helpers (like in the case of virophages), even though the effects are sometimes difficult to quantify.

L74: "coinfection of" to "coinfection with".

L73-75: I do not see the relevance of this sentence. Is recombination considered to be synergistic?

L80: Again, why do the authors claim synergy? How do SSV1 or SSV2 benefit from the presence of pSSVi/x?

L115: Please number the bands in 1E to avoid confusion.

L145: The tree shown in Fig. 3 is not a phylogeny, but rather a phylogenomic tree.

L167: "Almyro- for salty" – in which language?

L185: Archaeal homologs of eukaryotic TFIIB are called TFB (there is only one).

L191: I am not sure I understood the following correctly: "stretch that is found twice upstream and once downstream of Tebi-SV1 in the host genome". Please rephrase or show as a schematic in one of the figures. Typically, the attachment sites flank the provirus, i.e., two identical sequences on both sides of the provirus. The way it is written gives the impression that the sequence is repeated 3 times.

L228: sensitivity to chloroform does not necessarily mean the presence of lipids. Chloroform can also affect extensive hydrophobic protein-protein interactions, which underly the stability and structure of all spindle-shaped virions. The insensitivity of PV1 to chloroform is more surprising. Regardless, sensitivity patterns in Fig. 5A and 5B are the same. Thus, I do not understand why the authors conclude that only one of the viruses is sensitive to chloroform.

L270: Can the authors offer reasons why the viruses stopped replicating? For instance, do the strains contain CRISPR spacers against the two viruses?

L295: The authors should be cautious not to overinterpret cooccurrence of the two viruses. Given that pleolipoviruses are extremely abundant in haloarchaeal genomes, as has been reported previously, pretty much everything can be claimed to cooccur with them.

L292-312: Without additional evidence, I do not find this section to provide evidence of "interdependency" of the two viruses. The two viruses could be regulated in a similar way by similar transcription regulators. What is the relevance of the short nucleotide sequence shared between the two viruses (what is the identity? Show it as a panel in one of the figures?)? To substantiate the claims, the authors could clone the predicted regulatory sequences upstream of a reporter gene in a genetically tractable haloarchaeon and test if the transcriptional regulators from both viruses similarly affect the expression of the reporter gene. This would be quite a bit of work and I do NOT suggest performing the experiments for this manuscript. However, without additional evidence, it would be better to tone down the claims regarding the shared regulatory sequences and interdependence.

Given that the integrases are closely similar, do the two viruses share the attachment site?

L360: Check if the names are not inverted in Tebi-PV1:Tebi-SV1.

L373: It would be helpful if the authors could summarize the results of this section and make a conclusion. I was a bit lost what this all means.

L399: Superfamily, not family.

L408, "indicative of a lipid layer within the virus particle". None of the spindle-shaped viruses comprehensively studied thus far were found to contain lipid envelopes. Besides, the MCP of spindle-shaped viruses is predicted to be a membrane protein, but high resolution structures show that they are most definitely not in the membrane when in the virions. Thus, the authors should not be so definitive here.

L408-418: This is wildly speculative and unsubstantiated. I would suggest shortening the Discussion by removing overly speculative passages, such as this one.

L424: Note that MCP are not always the top proteins in MS analyses (due to different technical or biological reasons). For instance, the MCP of thaspiviruses was detected as one of the least abundant proteins by MS (likely dur to glycosylation and short size), whereas the most abundant structural protein of bicaudaviruses has nothing to do with capsid formation.

L426-435: This paragraph is also quite speculative and not rooted in robust evidence. For instance, have the authors excluded the possibility that the two integrases are paralogs (i.e., duplication happened in the ancestor of this virus group)? Have the authors considered the possibility that one of the enzymes functions as an integrase, whereas the other one is an invertase (as has been elegantly demonstrated in PhiCh1)?

L445: "we conclude that" to "we hypothesize that".

L447: The authors provide no evidence that the expression of the host pilins is in any way affected. If anything, the authors show that host pili are also produced (based on proteomics data). Thus, we do they claim that the pili formation is downregulated?

L504-508: Given that there is no conceivable mechanism how either of the two virion types would be assembled intracellularly, I am inclined to think that what the authors observe is not intracellular virions, but something else. For instance, mechanical lysis by sonication could lead to formation of membrane vesicles - not native EVs, but disrupted membranes closing on themselves and enclosing intracellular genomes. These would be resistant to nuclease treatment. To this reviewer, any alternative would be more believable than pleolipoviruses and spindle-shaped viruses assembling intracellularly. Electron tomography would be a way to provide definitive evidence of the assembly mechanism. In the absence of solid evidence, I would suggest refraining from unorthodox interpretations.

Version 1:

Reviewer comments:

Reviewer #2

(Remarks to the Author)
Although some parts of the manuscript still feel rather speculative the feedback has been sufficiently addressed and the speculative parts have either been toned down or been made clear to be speculation. The overall revised manuscript has greatly improved. I understand the limitations working with organisms that are genetically not (yet) accessible and thus some aspects can only theorized. I am pleased to see the addition of Fig. 4 to the manuscript. However, this visualisation of the shared region around the proposed operator reveals aspects that were not clear (misleading?) in the initially submitted version of the manuscript. I have thus one major concern (discussed below), that lies at the core of the manuscript, but can be addressed via textural changes. Given the high relevance of this manuscript for the field of (halo)archaeal virology, once this issue has been sufficiently addressed, I recommend this manuscript for publication.

Major point:
The new visual representation (Fig 4 B) reveals that the mutation (at -58/59 relative to the start codon) in the proposed operator region is in fact ca. 50 bp downstream of the homologous region (-106 to -207). I am willing to believe that the authors have identified an operator in Tebi-SV1 that upon mutation resulted in a change of regulation and subsequent massive enrichment of Tebi-SV1, but this operator sequence cannot be found in Tebi-PV1 as the 50 bp up- and downstream to this mutation are highly dissimilar between those two viruses. While the homologies region (-106 to -207) starting 50 bp upstream of the proposed operator could in theory have a regulatory functon(highly speculative), the authors thus do not present any evidence that the homologous region is in fact an operator region, it may just as well be coincidental.
Following the interpretative line of the manuscript that the mutation (at -58/59) would be the causal source for the greatly increased abundance of Tebi-SV1, it seems that Tebi-SV1 lost repression irrespective of Tebi-PV1 co-regulation.
I do not believe that this point detrimentally impacts the relevance of the manuscript. But the relatively frequently used claim that the operator sequence is "shared" while homology only starts 50 bp upstream of the proposed operator and subsequently interpreted co-regulation, must be adapted and is in the current state not supported by facts. I.e. Lines 42 or 648 and most importantly Lines 674 to 676: "Therefore, we hypothesize that the two proposed repressor proteins can bind each other's operator sequence allowing the viruses to co-regulate their genome copy numbers."

Minor points related to new Figure 4:

1. I would suggest adding predicted TATA boxes and Bre elements (if such motifs are detectable - not necessarily conserved in haloarchaea or sufficient information is missing).
2. It would be advisable to differentiate in the revised manuscript between mutations in the promoter and operator region (i.e. line 667). Since Fig 4 now nicely shows that the mutation is >55 bp upstream of the start codon and haloarchaeal transcripts are generally leaderless or have only very short leaders, the observed mutation it very unlikely in the (core) promoter region (i.e. mutated TATA/BRE), but as already suggested in the manuscript in the operator region.
3. It would be advisable to check (i.e. with MEME suite) if the proposed operator region (around 58/-59) has any of the typical operator patterns i.e. palindrome or (inverted) repeats. This could potentially even further strengthen the claims.

Reviewer #3

(Remarks to the Author)
I co-reviewed this manuscript with one of the reviewers who provided the listed reports. This is part of the Communications Biology initiative to facilitate training in peer review and to provide appropriate recognition for Early Career Researchers who co-review manuscripts.

Reviewer #4

(Remarks to the Author)
The authors adequately addressed all of my comments. I have no other suggestions.

Version 2:

Reviewer comments:

Reviewer #2

(Remarks to the Author)
The author's response is very convincing to me.
The annotation of operator stes is an excellent addition that now clearly shows the shared operators.
I now highly recommend the manuscript for publication.

One minor remark would be that the newly annotated BRE and TATA elements in Fig 4B have a very unusual distance to the respective start codons.
i.e. so close to the ATG that one would expect transcription to start downstream of the ATG.
Since this would add more confusion than benefit, I would omit it in the proofing stage.

Reviewer #3

(Remarks to the Author)
I co-reviewed this manuscript with one of the reviewers who provided the listed reports. This is part of the Communications Biology initiative to facilitate training in peer review and to provide appropriate recognition for Early Career Researchers who co-review manuscripts.

Open Access This Peer Review File is licensed under a Creative Commons Attribution 4.0 International License, which permits use, sharing, adaptation, distribution and reproduction in any medium or format, as long as you give appropriate credit to the original author(s) and the source, provide a link to the Creative Commons license, and indicate if changes were made.
In cases where reviewers are anonymous, credit should be given to 'Anonymous Referee' and the source.
The images or other third party material in this Peer Review File are included in the article's Creative Commons license, unless indicated otherwise in a credit line to the material. If material is not included in the article's Creative Commons license and your intended use is not permitted by statutory regulation or exceeds the permitted use, you will need to obtain permission directly from the copyright holder.
To view a copy of this license, visit https://creativecommons.org/licenses/by/4.0/

POINT-BY-POINT RESPONSES TO THE EDITOR AND REVIEWER COMMENTS

We want to thank the editor and the reviewers for their time to critically evaluate our work and to point out weaknesses and errors within the manuscript. With your effort, the manuscript has improved substantially. We have considered all suggestions and concerns of the reviewers as described below.

Reviewer #1 (Remarks to the Author):

Key results:

Authors discovered a fusiform virus-like particle (Tebi-SV1) in a Halorubrum strain but were unable to induce production of the particle. On analysis of DNA isolated from the culture, a second putative viral genome was found that was similar in sequence to known pleiomorphic halophilic viruses. The apparent genome of the larger particle is similar to genomes found by other researchers, either as plasmids or proviruses and is most convincingly similar to SSV_LN27. Interestingly, most of the putative coding sequences in Tebi-SV1 (and SSV-LN27) appear to be quite unique (BLAST e-value >10-5). The authors do not convincingly show virus replication in any other strains with the possible exception of Hrr. lacusprofundi. Putatively virus-encoded proteins are detected with mass spectrometry in cell-free extracts.

Validity:

I find the data presented to be valid and reasonable, but I find the data to be overinterpreted. (See comments below).

Response: Thank you for your positive feedback and for the very helpful critical assessment. We appreciate the time you investigated to improve the manuscript. We respond to the individual comments and concerns below.

Significance:

I think that new viruses of haloarchaea, particularly spindle-shaped ones, to be very interesting. That the Tebi-SV1 genome has very few ORFs with clear similarity to other viruses is striking and very interesting. The emphasis in the manuscript, particularly in the title, abstract and discussion on interactions between different viruses infecting the same host is not supported by the data presented. I do not find that many insights into virus-virus interactions can be gleaned from the data presented. Moreover, data presented in Xiang et al (reference 35) on SNJ1 and SNJ2, two viruses that infect a halophilic archaeon and encode pili, is not sufficiently discussed.

Response: Thank you for pointing out strengths of the manuscript.

In support of virus-virus interactions;

1. **We report on the abundance of the two viruses changing under different growth conditions with the abundance of one virus decreasing upon increase of the other virus. This is particularly pronounced in the first virus preparation which was dominated by Tebi-SV1 with low abundance of Tebi-PV1, in contrast to any of the following preparations with Tebi-PV1 dominating and a low abundance of Tebi-SV1.**

2. **Both viruses integrate into the host genome and share a putative repressor protein (confirmed by predicted tertiary structure comparison) that most likely regulates the lysis-lysogeny decision. The upstream region of these repressors shares a stretch of 101 bp that are 88 % identical, with long stretches of 100 % identity. This is now shown in Figure 4. We report on a mutation of Tebi-SV1 in the promotor region of the repressor, downstream of the proposed operator, which is only present on the virus genome isolated from the virus prep dominated by Tebi-SV1. This is supporting that the proposed repressor VP8 is indeed involved in the lysis-lysogeny decision.**
3. **Tebi-PV1 encodes for a pili protein, that was detected by MS. A virus pili protein regulating the relationship between SNJ1 and SNJ2 has been reported previously.**
4. **The host that contains the most closely related virus (to Tebi-SV1) also contains a second provirus – a pleolipovirus.**
5. **While both viruses poorly replicate in other host organisms – infection was always only detected with both viruses in all tested organisms.**

Xiang et al. is cited and discussed whenever appropriate including in the introduction and discussion. We added an additional statement now in the discussion.

Data and methodology:

Data presented are reasonable but do not sufficiently support the conclusions of the manuscript. The majority of the data presented are on characterization of each of the virus-like particles. Much of the data are based on qPCR experiments which are insufficiently described in the manuscript.

Thank you for pointing this out. We improved the description of the qPCR experiments in the methods (see also our response to your individual comments).

Analytical approach:

In general the analytical approach is appropriate, however evidence for viral replication (Figure 6) is not convincing.

Thank you. We address your concerns about viral replication by answering to comments below.

Suggested improvements:

General: I strongly recommend that host range studies be presented much earlier in the manuscript.

Thank you for this suggestion. The host range is presented right after the description of the individual viruses and the failed attempts to separate the two viruses from each other. We do not see how we could present this earlier in the manuscript, because the reader first needs the basic information on the two different viruses. Attempting to infect different host strains was also a logical consequence of the previous paragraph, because we were hoping to find a host that would only be susceptible to one of the two viruses.

Since none of the other reviewers feels this paragraph to be misplaced, we prefer to keep the current order of the data presented.

Specific:

L106: What is typical growth behavior? Please provide a reference or description.

Thank you for pointing out this gap. To clarify the typical growth behavior, we added Supplementary figure 1 showing representative examples of *Hrr*. TLS-6 growth behavior in different media.

L108: Please clarify if this TEM analysis was directly on supernatant (as implied in the text), or concentrated (as stated in the figure legend).

The analysis was done on the concentrated supernatant. We have corrected the text to "concentrated supernatant."

L110: These are very nice spindle-shaped VLPs, but seem somewhat pleiomorphic a discussion or analysis of TEMs should be presented.

We added a note about the pleomorphic nature of the spindle-shaped particles. 'Flexible' (pleomorphic) particles have also been described for haloarchaeal virus His1 (https://journals.asm.org/doi/10.1128/jvi.72.11.9392-9395.1998, https://www.pnas.org/doi/10.1073/pnas.1425008112). Elongates particles are already described in the following sentences in the manuscript.

L115-117: Please state order of bands from top or bottom or label in Figure 1E.

Thank you for pointing this out. We included this in the main text, labeled the bands with numbers in Figure 1 and rearranged the figure to improve reading.

L119: Please define scale bars in panels B, C and D. .

Scale bars are now defined in the figure legend.

L119: Please clarify what is meant by "first virus preparation"?

The 'first preparation' is in reference to the initial preparation abundant in spindle-shaped VLPs that was described in L105-110. The sentence was now changed to "40-25 % Iodixanol density gradient of the concentrated culture supernatant from growth retarded *Hrr.* TLS-6"

L122: Please clarify from which sample these VLPs were isolated.

We clarified that the sample used, "enriched in Tebi-PV1 particles" was concentrated supernatant of Hrr. TLS6 grown in nutrient rich medium at 28 °C.

L125: Please specify source of "total DNA", was this from band 2, supernatant or some other sample.

The "total DNA" was from band 2 of the gradient shown in Fig 1A. A sentence was added for clarification: "The total DNA from band two of gradient purified virus particles (Figure 1A), containing spindle-shaped VLPs (Figure 1B), was subject to high-throughput Illumina sequencing."

L132: Please clarify what is meant by "detected".

Please see below. Has been revised.

L132: Please clarify the difference between the genome sequence of the host and what was sequenced here. Apparently the host genome was resequenced (L646). Please clarify what was done in the text.

Thank you for pointing this out. We revised the text to describe this better to the reader. For this analysis we used the reads generated by a previous study on the host organisms in 2021 ((SRR12122876, SRA; doi:10.1007/s00248-020-01673-9) for a genome assembly of the host chromosome and searched this genome assembly for the two contigs assembled from the viral preparation. Resequencing of the host was done later in the study and is not mentioned here.

L142: Please define "significant similarity" and "high similarity".

See above. Has been revised.

L147-149: This section seems to repeat L132-134, Please clarify.

Thank you, indeed. We removed this section now. It was meant to point out that we do not find the contig as an integrated element in the host genome assembly despite encoding for an integrase. However, we did identify the integration site for Tebi-PV1 on the host genome now (an isoleucin-tRNA) which shares 15 bp with a region downstream of the integrase. We also identified a contig in the genome assembly containing this tRNA and the first 127 bp of the Tebi-PV1 genome, indicating that integration does occur. This is presented now in Figure 4.

L150: Please clarify what is meant by "it remains to be elucidated if Tebi-PV1 also can integrate into the host chromosome" if a provirus with identical sequence is found in the host genome.

As above: the paragraph has been changed now. While Tebi-PV1 is always assembled as a circular extrachromosomal element with a higher coverage than the host chromosome, we did identify the integration site in the *Hrr.* TLS-6 genome.

L153: "Functional annotation"? what does this mean?

Functional annotation means assigning predicted biological functions, roles, and attributes to genes or gene products based on evidence such as sequence similarity, conserved domains or experimental

data (https://doi.org/10.1371/journal.pcbi.1000431). We thought this expression is commonly used. We removed "functional" now from the sentence to avoid confusion.

L155: Please specify if amino acid identity is meant here.

Thank you for pointing this out. We clarified that amino acid identify is compared here.

L166: Virus families can only be named via the ICTV, please make an appropriate proposal and add "putative" or "proposed" here.

We are aware of this and a proposal will be made upon publication of the manuscript. The title of the paragraph indicates 'proposed'. We now also changed it to 'propose to name'.

L169: Figure 3: Sequences used in this tree must be identified by accession numbers and those used better described. The description in the figure legend and methods is insufficient to regenerate these data. The legend must also describe the color coding and scale of bar.

Supplementary table 1 with all accession numbers has been included. The color coding and the scale of bar is specified in the figure legend now.

L173: Gene sharing network analysis is not convincing. These data appear to show that Tebi-SV1 and SSV_LN27 are similar to each other, but very dissimilar to other sequences. I suggest removing this section (L173-182 and associated supplementary figures) or rewriting considerably.

Both gene sharing network analysis and genome-based phylogeny classification are standard tools to classify new viruses. Using both strengthens our statement that Tebi-SV1 is most similar to SSV-LN27 and more dissimilar to the recently discovered spindle-shaped viruses by Turgeman-Grott *et al*. We have agreed with the authors of this work on the proposed naming of the family.

While the analysis might be unnecessary to support our main statement it is not wrong, so we prefer to keep it. However, we shortened the text accordingly.

L192: Please provide references for this putative integrase-mediated circularization, for instance from alphafusellovirus SSV1.

Thank you for pointing this out. We reanalyzed the position of the repeats and the downstream repeat actually lies within the 3' end of a tRNA suggesting an integration mechanism similar to that of SNJ2 (https://doi.org/10.1111/mmi.13204). This is now described in the main text.

L195-199: While these differences between the genome sequence and that of the particles is interesting, it must be better described, particularly in terms of the confidence of the sequences, before SNPs can be confidently discussed.

The section has been revised accordingly.

L200-223, including Figure 4: These data are interesting, but the description could be greatly reduced and figure placed in supplementary data.

Thank you for this suggestion. The paragraph is very important to inform the reader about the change of virus-to-virus ratios in all subsequent cultures. Figure 4 illustrates that virus-to-virus ratios change under different growth condition with an increase of Tebi-PV1 production leading to a decrease of Tebi-SV1 production and *vice versa*. These data are important for our conclusion that the two viruses are co-regulated. The description is actually already very reduced, however, me moved Figure 4 into Supplements to make space for a Figure illustrating the integration sites and the shared operator sequence.

L202: Presumably this is band 2, but please specify.

Indeed, this refers to band 2. We have adjusted the sentence to, "The first virus preparation from growth retarded *Hrr*. TLS-6 cultures showed a high Tebi-SV1 to Tebi-PV1 ratio of 1,200:1 (based on sequencing coverage) in the gradient band subjected to sequencing (band 2 in Figure 1E).

L205: Please explain what is meant by "lysed cultures" and "reinfection". There is no mention of lysed cultures previous to this in the manuscript.

Thank you for pointing this out. This is referring to the original culture that did not reach exponential growth phase. Indeed, we do not know whether lysis or only strong growth retardation occurred in the culture. The sentence was been changed accordingly.

L206: Please clarify how this genome copy number was determined, also based on sequencing coverage?

The sentence has been revised to state that genome copy number was determined by qPCR.

L213: Please specify how "maintenance" was determined, presumably with PCR.

Yes, PCR was used to determine the presence and absence of viruses. The sentence has been revised.

L220: Please clarify what is meant by "different temperatures".

Thank you for pointing this out. This is a remnant from a previous version of the figure. This line has been removed.

L224-229: Details must be provided on how "removal" and "infectivity" was determined. I strongly recommend that the section on host range be moved to before this section.

We now refer to the individual Figures (Figure 4A-H) in the text to clarify how infectivity and integrity of the viral particles were determined. The approach is described in the figure legend: TEM of virus particles and qPCR of *Hrr. lacusprofundi* infected with treated and untreated virus particles. We clarify in the figure legend the nature of the viral preparations (Tebi-PV1 or Tebi-SV1 enriched). We also clarify the use of *Hrr. lacusprofundi* for infectivity assays in the main text. Though we do understand that moving the 'host range section' upfront would allow the reader to understand the use of *Hrr. lacusprofundi*. However, with the changes to the text we hope it is more understandable now. We prefer to keep the current order, as it is essential to describe the reader the changes to virus-virus ratios the earliest the possible, otherwise the host range experiments are also difficult to understand.

L240-243 (Figure 5): Electron micrographs are unconvincing.

We are very sorry, but without further details we cannot appropriately answer to this comment. We show both Tebi-SV1 and Tebi-PV1 virus particles in untreated samples vs. treated with chloroform and protease. We show that there were no obvious morphological changes to the virions that could be visualized in either treatment (e.g., elongation of Tebi-SV1 particles). We do appreciate that entirely disrupted particles would not be detectable by TEM, however, we present results from infectivity assays to confirm integrity of the majority of the virus particles. We changed the text accordingly with the hope that this is what is meant with 'unconvincing'.

L247: Please specify if this was virion-containing supernatant or gradient-purified particles. Gradient purification could degrade infectivity.

Thank you for pointing this out. The preparation was not CsCl purified. We specify now in the methods that the virus preparations used for host range assessment derived from cell lysate and was purified from cells and cell debris by centrifugation and filtration.

L249: These data appear to be virus genomes associated with cells, not "intracellular". Please clarify here and throughout the manuscript.

Thank you for pointing this out. Indeed, we cannot distinguish between cell associated and intracellular numbers using this method. "Intracellularly" has been changed to "cell associated virus" or "virus associated with cells" throughout the manuscript.

L254-257: This description can be removed, since the previous sentence is sufficient.

This sentence was removed to avoid repetition.

L257: Please provide how "significance" was determined.

We removed 'significant' as a statistical test was not performed. Ratios ranged from 210:1 to 5:1 as described in the next sentence.

L257: Please state clearly how "intracellular" and "extracellular" virion numbers were counted. In the methods, for extracellular particles, this appears to be a resuspended ultracentrifugation pellet that has been treated with DNAse. Relative quantification of this sample relative to washed cells is problematic and should be stated as such.

This is stated in the Methods:

For "intracellular": "Cells from 1.5 ml of culture were harvested by centrifugation (10,000 x g, 10 min), washed twice with fresh media (to remove any free viruses), and screened for infection with Tebi-SV1 and Tebi-PV1 with specific qPCR probes (Supplementary table 10)."

For "extracellular":

"The supernatant was filtered twice with 0.22 µm syringe filters, and virus particles were harvested by ultracentrifugation (41,000 rpm for 2 h, 4 °C, fixed angle 70.1 Ti rotor, Beckman & Coulter). The pellets were resuspended in 100 µL of DBCM2 salt solution and treated with DNase I before DNA extraction and quantification by qPCR."

We do not put intracellular with extracellular virus numbers in relation. We either compare Tebi-SV1 to Tebi-PV1 ratios or host genome to virus genome ratios in the exact same sample. Therefore, we do not understand the concern.

For "extracellular copy numbers" the culture supernatant was filtered twice through 0.22um pore size filters prior to ultracentrifugation and resuspending the pellet. This procedure was efficient to remove any intact cells. We have tested this rigorously for all virus preparations used for infection (last sentence in "*Isolation and purification of viruses*") "Virus preparations were tested for host contamination both by PCR and by incubation in medium for several days before further use." Therefore, we are convinced that there was no major cellular contamination in our "extracellular" preparations, though indeed we did not also test them for host genome copy numbers by qPCR.

L280: Figure 6: For all tested hosts, with the exception of Hrr. Lacusprofundi, the number of virus genome copies is multiple orders of magnitude lower at the second time point. Thus, there is no indication that virus replication is taking place.

We do understand the skepticism of the reviewer. Viral numbers are in general low for all experiments, and indeed, as we mention in the text, for all tested host organisms, virus numbers go down and the viruses are most often excluded after long-term cultivation. However, as mentioned in, we calculated the total number of detectable viral genomes (sum of cell associated and extracellular genome copy numbers in the 50 mL culture). For Tebi-SV1 these numbers were higher than the total number of virus genomes added to the experiment for infection in *H. japonica* and were similar in *Hfx. volcanii*. Total numbers of Tebi-PV1 were similar in *Hfx. mediteranei* and *Haa. japonica*. Bearing in mind that not all virus particles bind to cells, that washing of cells after initial adsorption and loss of material during sample processing (isolation and purification of cells and virus, DNA extraction) leads to a significant decrease in viral genome copy numbers we consider this indicative of virus replication. Additionally, we calculated total numbers of free virus particles in the supernatant after diluting the cultures to 0.05 (TP2) and allowing a second growth cycle. Comparing extracellular virus numbers from first cultures and second cultures after considering the dilution factor extracellular virus numbers did increase for Tebi-SV1 in *Hrr. lacusprofundi* and for Tebi-PV1 in *Hrr. lacusprofundi*, *Hbt. salinarium* and *Hfx. volcanii*, indicative of virus particle production.

We have now added a Supplementary table 5 to better visualize these data, and also described the calculation in the Methods.

This is clear evidence that replication must have taken place for Tebi-SV1 in *Hrr. lacusprofundi* and *Haa. japonica*, and for Tebi-PV1 in *Hrr. lacusprofundi*, *Hbt. salinarium* and *Hfx. volcanii*.

In support of the approach (using qPCR): We used a very similar approach (qPCR) for other viruses infecting *Hrr. lacusprofundi*. For HRTV-DL1 – lytic virus- (doi: 10.3389/fmicb.2023.1274068) virus attachment was proven by adsorption assays, but the virus was not able to replicate and produce virus particles in one of the tested strains (ACAM34_DSMZ). Assays using qPCR show a very quick removal of the viral genome from host cells (56 hours post infection) and no virus particles in the culture supernatant, while in case of Tebi-SV1 and Tebi-PV1 we can still detect both cell associated and free virus genomes more than 200 hours post infection. For HFPV-1 – pleolipovirus - (doi: 10.3390/v15071469), we also detected very low virus-to-host ratios in the cells and in the supernatant over extended time periods, however, in this case the virus was not excluded over long-term.

L383-Discussion.

I think that this section should be rewritten after critical analysis of results.

We have carefully revised the discussion, considering all reviewers suggestions.

L391-: As stated above, please submit a taxonomic proposal to the ICTV and state that these are proposed in the text.

As stated above, we will submit a proposal upon publication of the manuscript. The name of the family was confirmed with the authors of Turgeman-Grott *et al*. We included "proposed" into the sentence.

L408: Please provide a citation for the statement suggesting a lipid layer.

A citation was added and the statement has been changed.

L409: Please provide details on how similar this protein is to HEPN toxin proteins. Without these data it is impossible to interpret the proposed functions.

A respective statement has been added in the results section (*Protein composition of virus particles*). The structure was predicted with Alphafold 3 and searched against the database with DALI – two representative hits are included with the respective alignment scores. However, we now removed this section from the manuscript, as it was considered too speculative and indeed does not add much to the main focus of the manuscript.

L419: Please either show or describe these structural characteristics. Are they more than the 2 predicted transmembrane domains shown in Figure S8?

We describe the analysis of VP35 in the results section (*Protein composition of virus particles*). We now included the predicted structure into Supplementary Figure 10.

L461: See previous comments regarding infectivity. I am not convinced that these viruses are infecting other strains than Halorubrum.

We have answered to these comments earlier. However, we have now changed the wording in the paragraph to express that we have no doubt that the viruses can attach to the host organisms. However, we only infer on virus particle production for the host organisms for which extracellular copy numbers increased (after including the dilution factor). The heading needs no change, as it only mentions the receptor and no 'injection, replication, virus particle production'. While we have no doubt that infection is taking place, we hope that this is sufficient down-toning to satisfy your concerns.

L487-: If other strains are being infected with these viruses, it should be possible to determine whether infection is chronic or lytic. If this is not the case, then this section is speculative.

The virus genome copy number-to-host ratio in all the other strains was very low (always below 1). Unfortunately, we never reached anything higher than that as viral yields from *Hrr.* TLS-6 are generally low. Additionally, in long-term we lose the viruses, in particular Tebi-SV1, from all these cultures. None of the tested strains turned out to be a reasonably good host that we can use to characterize the two viruses. The low virus genome copy number-to-host ratio also makes it impossible to judge on chronic or lytic – with a minority of the cells being infected and producing a low amount of virus particles, this would not be visible in the growth of the culture even if Tebi-SV1 would be lytic.

Regarding TLS-6 as host: Due to the complexity of this virus-virus system and the presence of both viruses, it is difficult to distinguish the life cycle of each virus individually. We can more accurately describe a chronic infection by the dominating Tebi-PV1 since viral titers are reasonable high without obvious cell lysis. The life cycle of spindle-shaped viruses, such as His1 or SSV1, was also debated for decades and published as both chronic and lytic in different peer-reviewed articles. Tebi-SV1, in particular is no different. The initial culture from which Tebi-SV1 was isolated, exhibited extreme growth inhibition. In subsequent cultures, where Tebi-SV1 no longer dominated the samples (but was still present) the cultures reached stationary phase. Unfortunately, there is no "virus-free" control strain available of *Hrr.* TLS-6 for a comparative analysis of uninfected vs. infected *Hrr.* TLS-6. While we did attempt to make TLS-6 genetically tractable, we have not yet succeeded.

L524-: For all of the protein sequences discussed in this section (and the remainder of the discussion), please provide data on sequence similarity. This could possibly be in a (supplemental) table.

This is now specified in the results section. Tertiary structure prediction and alignment shows that both proteins are highly similar to the structure of a PhiH-like repressor protein, to ORF4 of SNJ2 as well as sharing a similar structure with each other.

L536-538: This interesting result needs to be more clearly presented in the text.

This is now described more detailed in the results. We also included a figure (Figure 4) to better illustrate the similarities of the operator sequence between Tebi-SV1 VP8 and Tebi-PV1 VP4. The mutation occurred in the promotor sequence of Tebi-SV1. We now also discuss this accordingly.

L543: Please clarify what data are being referred to here.

We refer to the data discussed in L536-538 (see above). We modified the section to improve reading and added a graphic illustration (Figure 4).

L539-540: Please be precise about what is shared and what is not.

The sentence has been re-written.

L539: Please clarify that VP8 is a predicted transcriptional regulator.

The paragraph has been modified.

L620: This appears to be artificially lysed cells (spheroplast + sonication). Please be clear about how this was generated in the text. Moreover, if I understand correctly, particles from artificial lysis were concentrated with ultracentrifugation. This should also be clearly stated in the text.

We included the word 'artificial' in this sentence and specified the nature of the preparations (e.g. spheroplast lysis) also throughout the main text.

L664: Please restate which preparations were used here, from the iodixanol gradient?

The nature of each of the virus preparations is stated in the legend of Supplementary Figure 9 and specified in Supplementary Table 7. We now refer to this in the text. Restating this all in the text would rather confuse the reader.

L695: This procedure does not guarantee that these viral genomes have entered cells, just that the virus genome is associated with cells after washing.

The sentence was changed to, "DNA was extracted from cell pellets, and virus genomes associated with cells after washing were detected *via* qPCR."

L709: Please clarify how MOI was determined, was this based on qPCR?

Thank you for pointing this out, we have changed MOI to "genome copy number". This was a remnant from an earlier version of the manuscript. We cannot infer MOI from genome copy numbers.

L715: Please provide data on how "optimal" was shown or delete.

The word, "optimal" was removed from the sentence.

L800: Typographical error in reference. "ulfolobus" should be "Sulfolobus"

Thank you. The typographical error was corrected.

L857: Suggest including bioRxiv reference here instead of doi.

The manuscript is published now and we have included the correct citation.

Supplemental material:

L10: Details of how this figure was generated, particularly “shared protein content” should be shown. Genomes that are labeled should be described.

Details on how this figure was generated are described in the methods. Genome description is included now.

L34: Supplemental Figure 4: Figure legend seems to be missing for panels C and D. qPCR data appears to be missing from panels C and D.

We corrected the figure legend accordingly and the main text. qPCR on the culture supernatant was not performed for C and D, therefore, no data can be presented.

L46: Supplemental Figure 5: Please be consistent in labels of Y axes. See comment to text line 257.

Figure legends have been corrected. The word “intracellular” was changed to cell associated.

L105: Supplemental Figure 10: Please provide statistics for how often Tebi-PV particles are observed associated with putative cellular appendages.

Unfortunately, transmission electron microscopy analysis was only qualitative; thus, we do not have statistics available. We observed this consistently within the same sample as well as across multiple samples.

References

While only just published, the authors should carefully review and comment on Zhao et al., 2025 (https://www.nature.com/articles/s41564-025-02149-7) in terms of their results. A preprint of this manuscript was available in February 2025.

Thank you for this suggestion. We have enjoyed reading this work, but we do not see an immediate context between these newly discovered virus genomes and the characterization of two isolated viruses from a different host organisms and location.

Reviewer #2 (Remarks to the Author):

The authors provide a compelling study of relevance to the archaeal community. Especially since characterizations of virus-virus interactions in archaea are still sparse, these findings would benefit future viral research. While the experimental approaches are solid and well documented the irreproducible outcome of the culture that was used to produce the data for Figure 1 stands in stark contrast to the later experimental work in which the ratio between the viruses is inversed. Additionally, some of the claims seem somewhat speculative and are not fully backed up by experimental data.

Thank you very much or taking the time to assess our work and for all the helpful comments.

To address your major concern: Indeed, we were not able to reproduce the outcome from the first culture, and since we are currently not able to genetically manipulate the host organisms (work in progress for a couple of years) we can also not backup some of your claims. However, to our opinion this does not weaken the data and data interpretation presented. While we could remove data about the first culture and simply present the virus-host system in its current state for which we can present biological replicates, the research community would lose a lot of important information on how these virus-host systems can spontaneously reshape. We avoid making unreasonable claims and clearly state that some of the presented data could not be reproduced.

Comments to the Author:

A high Tebi-SV1 to Tebi-PV1 ratio of 1200:1 in the culture that was used for TEM imaging of Fig.1 compared to any follow up experiments with completely different average ratios of 1:1760, raises the question if these TEM images are representable for this viral system. Especially since the Tebi-SV1 genome of the first preparation had two SNPs in an apparent operator region. Since TEM imaging of other cultures has been performed i.e. Fig S9, I would strongly urge the authors to directly address this point early in the manuscript and possibly provide TEM images of a culture that reflects the (viral ratios of) rest of the study to alleviate any doubts about the findings shown in Fig. 1., or at least link to supplemental Images for comparison.

Thank you for sharing your concerns. To further highlight that the first preparation was unique, we added the sentence into the first paragraph: "After the initial isolation, such high abundances of the spindle-shaped VLP was not observed again." In Figure 1 we specify already that the electron micrograph is of the initial virus preparation where the *Hrr.* TLS-6 culture did not reach exponential growth and the supernatant was dominated by Tebi-SV1 particles. We would like to keep this figure

as it is meant to highlight the newly isolated spindle-shaped viruses. Isolates of spindle-shaped viruses are rare while many pleolipoviruses are available. We do show several other electron micrographs. In Figure 5 we show supernatants of Tebi-PV1 enriched and Tebi-SV1 enriched viral preparations, that also contain many Tebi-PV1 particles. We now highlight this more specifically in the legend of Figure 5. Additional TEM pictures are presented in Figure 7 from cellular lysate preparations. We also now added an additional figure illustrating the integration mechanisms and the mutations found in the virus genome of this first viral preparation (Figure 4).

Since all four reviewers did understand that the first preparation is not representative of all the remaining viral preparations, we infer that this will also be obvious to any other reader.

Regarding the hypothesis in Line 42-44 (and Line 524ff.): "Virus abundances seem to be regulated through a shared operator sequence allowing Tebi-PV1 to keep 44 Tebi-SV1 copy numbers low without excluding Tebi-SV1 from the host genome." While this claim could significantly elevate the impact of this study, unfortunately there is only limited experimental evidence suggesting this. The observation that a mutant Tebi-SV1 with a 2 bp variance in a shared homology region Tebi-PV1 shows a drastically increased relative abundance is indeed intriguing. But whether the observed phenotype is due to an altered auto- or co-regulation or unintended experimental variation still remains to be elucidated, since no evidence has been delivered that the region of interest is in fact an operator.

Indeed, without a targeted mutation of this region we cannot claim with confidence that the mutation is the cause for the dramatic changes in the ratio between the two viruses. Since we are not able to manipulate the virus genome within the original host strain, we will certainly attempt to study the putative operator/promotor region and the two putative repressors in a genetically tractable system in a follow-up study.

To better illustrate the genomic region of the putative operator, we now included an additional figure (Figure 4).

However, we do infer that the drastically increased relative abundance is indeed due to the spontaneous mutation, as none of our attempts to replicate this observed phenomenon for over three years using the exact same conditions and a large variety of other growth conditions yielded relative abundance that were any close to what we observed in this unique culture that was grown from a single colony. Additionally, our data showing that viral abundances co-vary under different growth conditions, namely more Tebi-SV1 less Tebi-PV1 and *vice versa*, strongly support our hypothesis

about a co-regulation. To accommodate the reviewers concerns we toned down the text related to this hypothesis in the abstract and the discussion.

The low effectiveness of Proteinase K and chloroform treatment in deactivating the viral particles is surprising and should be addressed in the discussion, perhaps with references how this relates to other viruses.

Thank you for pointing this out. Despite their lipid membrane, insensitivity to chloroform treatment has been reported for other pleolipoviruses (https://doi.org/10.3390/v7041902, https://doi.org/10.1111/1462-2920.14604). Halophilic spindle-shaped virus, His1 was sensitive to chloroform treatment (1:4), where no viable virus was detected after only 2.5 and 5 min (https://doi.org/10.1128/jvi.72.11.9392-9395.1998). Chloroform treatment of Tebi-SV1 (1:4) showed a 2-fold reduction of infectivity.

Proteinase resistance is also not uncommon for archaeal viruses. For example, Thermoproteus spherical piliferous virus 1 (TSPV1) particles do not show changes to protein composition upon Proteinase K treatment (https://doi.org/10.1128/jvi.00036-20). Proteinase K was shown to effect fusion of the pleolipovirus HRPV-6 with its host due to degradation of the spike protein (doi.org/10.1038/s41467-019-08728-7), however, the spike proteins also changed conformation upon temperature changes which could have an impact on proteinase K sensitivity.

We added information on this into the main text and the discussion.

Throughout the manuscript qPCR on washed cell pellets is being used to quantify viral DNA as an indication for successful infection. As washing would not remove adsorbed/surface attached viruses that have not (yet) completely fused with the host or injected their genome, it seems that this approach would manly quantify the adsorbed fraction. A clarification on this would be important, as adsorption without genome injection is a potential outcome with alternative hosts.

Thank you. This has also been pointed out by another reviewer. We have changed "intracellular" to "cell associated" throughout the entire text.

Additionally, the interpretation of the data in Figure 6 would highly benefit if (at least semi-quantitative) statements could be made if or how much the total virus genome copy numbers changed throughout the experiment, i.e. how much did the virus(es) actually amplify?

We have included Supplementary table 5 with the total virus genome copy number added to the experiment and the total amount of virus genome copy number detected in the culture at an indicated time point post infection. Additionally, we calculated the changes in extracellular virus numbers after diluting the cultures, including the dilution factor, supporting virus production in several host organisms.

Minor points:

· Supplementary Table 3 has “Supplementary table 2” in its header

· Line 355 the opening bracket “(“ of (Supplementary Figure 9) is bold.

Thank you. Has been corrected.

· I think a minor editing error occurred in the legend of Fig. 1: Perhaps the mentioning of panels (B-C) slipped into the sentence before and was meant to be liked to “… . (B-C) Tebi-SV1 particles exhibiting pleomorphism with elongated tails and in a tubular form (size bars 122 are 100 nm) from band 3 of Iodixanol gradient (E).”

This was corrected.

· The software/tool that was used for the secondary structure search in order to improve the viral genome annotation appears to not be mentioned.

We used Alphafold 3 and Dali if not specified otherwise in the main text. Thank you, indeed this should be described in methods and we now included a sentence in the methods section.

Reviewer #3 (Remarks to the Author):

I co-reviewed this manuscript with one of the reviewers who provided the listed reports. This is part of the Communications Biology initiative to facilitate training in peer review and to provide appropriate recognition for Early Career Researchers who co-review manuscripts.

Thank you very much for your time and input. We certainly appreciate input from researchers in various stages of their career.

Reviewer #4 (Remarks to the Author):

Queiss and colleagues report the isolation of two new viruses infecting an extremely halophilic archaeon, Halorubrum sp. TLS-6. One of the two viruses has a pleomorphic morphology and belong to the existing family Pleolipoviridae, whereas the second virus produces spindle-shaped virions and is a founding member of a novel virus family, which the authors tentatively named 'Almyrocitroviridae'. The two viruses are engaged in a complex relationship, and I would like to commend the authors for their efforts to unravel it in a non-model organism, such as Halorubrum sp. TLS-6. It is increasingly clear that virus-host interactions are more complex that usually considered and typically involve multiple coinfecting viruses. Thus, this work will be of great interest to the community of researchers working on archaea as well as those working on prokaryotic viruses. The manuscript is largely clearly written. However, some of the interpretations are overly speculative and should be tightened, as detailed below in my relatively minor comments.

We would like to thank you very much for your time and for the highly constructive feedback. We particularly appreciate the reviewer's understanding of the challenges associated with this complex non-model system.

L37,39: The capitalization in Tebenquiche spindle-shaped Virus 1 (Tebi-SV1) and Tebenquiche pleomorphic Virus 1 (Tebi-PV1) appears random. Either capitalize all words used for abbreviation or only the proper nouns. To this reviewer, removing capitalization from Virus would be the more appropriate solution.

This was corrected.

L42: In the absence of evidence of actual mutualism between the two viruses, it might be more appropriate to state that the relationship is symbiotic.

We changed the sentence to "symbiotic".

L71-73: Interaction between satellite viruses and helper viruses is not cooperative. Satellite viruses are typically parasites of their helpers (like in the case of virophages), even though the effects are sometimes difficult to quantify.

While this may be true for satellite viruses, such as some virophages which we do not explicitly mention, we do not find evidence in the literature that satellite viruses such as, pSSVx cause a reduction of SSV1 or SSV2 titers upon coinfection. However, we have changed "cooperative" to "commensal".

L74: "coinfection of" to "coinfection with".

We changed the sentence.

L73-75: I do not see the relevance of this sentence. Is recombination considered to be synergistic?

Recombination does often have 'beneficial' effects for viruses as it can create gene variants that allow for better propagation, broadening of host range or increased resistance to host defense systems (e.g. diversity of Methyltransferases). Best examples are eukaryotic viruses such as HIV (https://doi.org/10.1186/s12879-024-09805-z) and Sars-CoV2 (https://doi.org/10.1038/s41467-023-43391-z), or https://doi.org/10.1016/j.virusres.2023.199272 and https://doi.org/10.1371/journal.pbio.3003515 where recombination between different phages leads to broadening host range. The paper cited here describes the lab evolution of an environmental virus mixture that constantly changed as long as several variants remained in the cocktail towards a single isolate that caused an exclusively lytic infection of its host, though other variant contained integrases.

We changed the wording slightly to improve the understanding of the statement and added another citation.

L80: Again, why do the authors claim synergy? How do SSV1 or SSV2 benefit from the presence of pSSVi/x?

"Synergistic" was changed to "commensal".

L115: Please number the bands in 1E to avoid confusion.

We have adjusted the figure accordingly with labeling the bands in the gradient.

L145: The tree shown in Fig. 3 is not a phylogeny, but rather a phylogenomic tree.

Indeed, also Tebi-PV1 is not included in this tree. We changed the sentence and refer to the respective figures.

L167: "Almyro- for salty" – in which language?

Greek. We added this information.

L185: Archaeal homologs of eukaryotic TFIIB are called TFB (there is only one).

Thank you. We changed this.

L191: I am not sure I understood the following correctly: "stretch that is found twice upstream and once downstream of Tebi-SV1 in the host genome". Please rephrase or show as a schematic in one of the figures.

Typically, the attachment sites flank the provirus, i.e., two identical sequences on both sides of the provirus. The way it is written gives the impression that the sequence is repeated 3 times.

We rephrased the entire section now. The attL/attR and attP could be identified. We also added a figure to illustrate the insertion site (Figure 4).

L228: sensitivity to chloroform does not necessarily mean the presence of lipids. Chloroform can also affect extensive hydrophobic protein-protein interactions, which underly the stability and structure of all spindle-shaped virions. The insensitivity of PV1 to chloroform is more surprising. Regardless, sensitivity patterns in Fig. 5A and 5B are the same. Thus, I do not understand why the authors conclude that only one of the viruses is sensitive to chloroform.

Thank you for pointing this out. Sensitivity of Tebi-SV1 is much higher than of Tevi-PV1 and neither of them are actually inactivated. The statement was initially referring to 'inactivation' of virus particles, rather than general stability.

We rephrased the sentence now and include the possible effect of chloroform on hydrophobic protein-protein interactions in the discussion

L270: Can the authors offer reasons why the viruses stopped replicating? For instance, do the strains contain CRISPR spacers against the two viruses?

Thank you for the question. We analyzed the CRISPR of *Hfx. volcanii, Hfx. mediterranei and Hqt. walsbyi*. We only found a partial match for Tebi-SV1 in *Hfx. volcanii*, however, its likely not effective against the virus (mismatches, and too short). We now included this information.

L295: The authors should be cautious not to overinterpret cooccurrence of the two viruses. Given that pleolipoviruses are extremely abundant in haloarchaeal genomes, as has been reported previously, pretty much everything can be claimed to cooccur with them.

We totally agree that the cooccurrence is not evidence for a general symbiotic relationship between pleolipoviruses and spindle-shaped viruses. However, the aim is to highlight pleolipoviruses as potential important regulators of virus-host relationships in haloarchaea, which is supported by to other works (https://doi.org/10.1073/pnas.2205037119, https://doi.org/10.1016/j.celrep.2025.115873). We now included a respective statement into this section and the discussion.

L292-312: Without additional evidence, I do not find this section to provide evidence of "interdependency" of the two viruses. The two viruses could be regulated in a similar way by similar transcription regulators. What is the relevance of the short nucleotide sequence shared between the two viruses (what is the identity?

Show it as a panel in one of the figures?)? To substantiate the claims, the authors could clone the predicted regulatory sequences upstream of a reporter gene in a genetically tractable haloarchaeon and test if the transcriptional regulators from both viruses similarly affect the expression of the reporter gene. This would be quite a bit of work and I do NOT suggest performing the experiments for this manuscript. However, without additional evidence, it would be better to tone down the claims regarding the shared regulatory sequences and interdependence.

We now included a figure to illustrate the similarities between the two regions and the position of the mutations. Since we currently cannot provide further evidence, we also toned down the claims in the discussion and the abstract.

Given that the integrases are closely similar, do the two viruses share the attachment site?

The attachment site for Tebi-PV1 is different from Tebi-SV1. Tebi-PV1 integrates into an isoleucine t-RNA that is located at the start of the longest contig representing the main chromosome of TLS-6. We now included this information into the manuscript.

L360: Check if the names are not inverted in Tebi-PV1:Tebi-SV1.

This was corrected.

L373: It would be helpful if the authors could summarize the results of this section and make a conclusion. I was a bit lost what this all means.

Thank you for making us aware of this. We added two summarizing sentences. Additionally, we also discuss our conclusion from these results in the discussion section, "*Chronic or lytic? – the life cycle of Tebi-SV1 remains obscure"*

L399: Superfamily, not family.

This was changed.

L408, "indicative of a lipid layer within the virus particle". None of the spindle-shaped viruses comprehensively studied thus far were found to contain lipid envelopes. Besides, the MCP of spindle-shaped viruses is predicted to be a membrane protein, but high resolution structures show that they are most definitely not in the membrane when in the virions. Thus, the authors should not be so definitive here.

We have adjusted "a lipid layer" to "lipids" in the sentence.

L408-418: This is wildly speculative and unsubstantiated. I would suggest shortening the Discussion by removing overly speculative passages, such as this one.

Thank you. We do agree and shortened this section.

L424: Note that MCP are not always the top proteins in MS analyses (due to different technical or biological reasons). For instance, the MCP of thaspiviruses was detected as one of the least abundant proteins by MS (likely dur to glycosylation and short size), whereas the most abundant structural protein of bicaudaviruses has nothing to do with capsid formation.

We are aware of this and do discuss exactly this in the respective paragraph (post-translational modifications). We cite the paper that has shown that MCPs are modified which could be the reason why they are not detected as most abundant proteins. We do not see the point of extending this discussion towards technical issues.

L426-435: This paragraph is also quite speculative and not rooted in robust evidence. For instance, have the authors excluded the possibility that the two integrases are paralogs (i.e., duplication happened in the ancestor of this virus group)? Have the authors considered the possibility that one of the enzymes functions as an integrase, whereas the other one is an invertase (as has been elegantly demonstrated in PhiCh1)?

We removed the paragraph. We agree that it is speculative and does not add much to the main hypothesis of the work. We added a short sentence describing the second integrase after the description of the integration mechanism. However, repeats, as reported for PhiCh1, could not be detected in the proximity of the integrase.

L445: “we conclude that” to “we hypothesize that”.

L447: The authors provide no evidence that the expression of the host pilins is in any way affected. If anything, the authors show that host pili are also produced (based on proteomics data). Thus, we do they claim that the pili formation is downregulated?

This refers to the work described in the previous sentences, not to Tebi-PV1. It was shown that knockout of proviruses in *Haloferax volcanii* lead to increased motility of the strain, with transcriptomics data supporting the upregulation of Pili genes upon provirus deletion. We now refer to ‘some pleolipoviruses’ to prevent the reader from getting the impression that we are referring to Tebi-PV1.

L504-508: Given that there is no conceivable mechanism how either of the two virion types would be assembled intracellularly, I am inclined to think that what the authors observe is not intracellular virions, but something else. For instance, mechanical lysis by sonication could lead to formation of membrane vesicles - not native EVs, but disrupted membranes closing on themselves and enclosing intracellular genomes. These would be resistant to nuclease treatment. To this reviewer, any alternative would be more believable than pleolipoviruses and spindle-shaped viruses assembling intracellularly. Electron tomography would be a way to provide definitive evidence of the assembly mechanism. In the absence of solid evidence, I would suggest refraining from unorthodox interpretations.

We do not hypothesize that pleolipoviruses assemble intracellularly. We totally agree that this is not the case. We actually included an entire paragraph discussing how we explain the presence of Tebi-PV1 in the spheroplasts fraction. The same may potentially apply to Tebi-SV1 and we now included a sentence stating this.

However, we were not successful in transforming the Tebi-SV1 genome into one of the host strains and we did not observe Tebi-SV1 to associate with host pili as shown for Tebi-PV1. We observed intact Tebi-SV1 articles in the spheroplasts fraction. While we are totally aware of the work on other spindle-shaped virus particles, including the exit mechanisms of SSV, we do not agree that an intracellular assembly is entirely impossible for a spindle-shaped virus.

In contrast to Crenarchaeota/Thermoproteota, no ESCRT proteins have been thus far described in haloarchaea. No genes are shared between Tebi-SV1 and any other known spindle-shaped virus for which an exit strategy has been described. Chloroform treatment inactivates His1, while it does not inactivate Tebi-SV1. Considering that spindle-shaped viruses may have evolved from rod-shaped viruses (https://doi.org/10.1016/j.cell.2022.02.019), some of them may well share their exit strategy. Tebi-SV1 contains a number of small TM domain containing proteins and, for example, gp21 gives partially aligns (HHpred) with the pyramid forming protein of SIRV2 and STIV. Unfortunately, these small TM proteins do not result in reasonably good predicted structures, so function can only be assessed experimentally. However, we aim to overexpress respective candidates in *Haloferax volcanii* in a follow-up study.

Attempts were made to perform Cryo-ET on the infected host cells, however, due to time constraints and the difficulty of the system (contrast issues with high-salt), this was not successful. Please see the dissertation for more details regarding Cryo-ET (https://media.suub.uni-bremen.de/entities/publication/0d9eeac3-e42e-4e53-aea8-8fa9474a6be6. Also, classical embedding and sectioning did not resolve any virus particles – neither attached to the host cell, nor within the

host cells. We assume that is either due to the low abundance of the virus, or the destruction of virus particles during sample preparation.

POINT-BY-POINT RESPONSES REVIEWER COMMENTS

Reviewer #2 (Remarks to the Author):

Although some parts of the manuscript still feel rather speculative the feedback has been sufficiently addressed and the speculative parts have either been toned down or been made clear to be speculation. The overall revised manuscript has greatly improved. I understand the limitations working with organisms that are genetically not (yet) accessible and thus some aspects can only theorized. I am pleased to see the addition of Fig. 4 to the manuscript. However, this visualisation of the shared region around the proposed operator reveals aspects that were not clear (misleading?) in the initially submitted version of the manuscript. I have thus one major concern (discussed below), that lies at the core of the manuscript, but can be addressed via textural changes. Given the high relevance of this manuscript for the field of (halo)archaeal virology, once this issue has been sufficiently addressed, I recommend this manuscript for publication.

Thank you very much for your time reassessing our manuscript.

Major point:

The new visual representation (Fig 4 B) reveals that the mutation (at -58/59 relative to the start codon) in the proposed operator region is in fact ca. 50 bp downstream of the homologous region (-106 to -207). I am willing to believe that the authors have identified an operator in Tebi-SV1 that upon mutation resulted in a change of regulation and subsequent massive enrichment of Tebi-SV1, but this operator sequence cannot be found in Tebi-PV1 as the 50 bp up- and downstream to this mutation are highly dissimilar between those two viruses. While the homologies region (-106 to -207) starting 50 bp upstream of the proposed operator could in theory have a regulatory functon (highly speculative), the authors thus do not present any evidence that the homologous region is in fact an operator region, it may just as well be coincidental.
Following the interpretative line of the manuscript that the mutation (at -58/59) would be the causal source for the greatly increased abundance of Tebi-SV1, it seems that Tebi-SV1 lost repression irrespective of Tebi-PV1 co-regulation.
I do not believe that this point detrimentally impacts the relevance of the manuscript. But the relatively frequently used claim that the operator sequence is “shared” while homology only starts 50 bp upstream of the proposed operator and subsequently interpreted co-regulation, must be adapted and is in the current state not supported by facts. I.e. Lines 42 or 648 and most importantly Lines 674 to 676: “Therefore, we hypothesize that the two proposed repressor proteins can bind each other’s operator sequence allowing the viruses to co-regulate their genome copy numbers.”

Thank you very much for pointing out your concerns about the details on the regulatory region. We have reinvestigated this region and identified all promotor sequences and the operator sequences. This is now presented in Figure 4B (see below), and we adjusted the results section and the discussion accordingly.

We identified the operator sequences for both viruses. They are represented by inverted repeats. The operator sequence in Tebi-SV1 is located between the promotor of the repressor and the promotor of the integrase operon. For Tebi-PV1 we could only identify one promotor that likely serves for both the repressor and the integrase operon. The operator is located downstream of the promotor for the integrase operon.

The first 5 bp of the Tebi-SV1 operator are 100% identical to the Tebi-PV1 operator and the reported mutation is located within these 5 bp. This now clearly supports our hypothesis that this mutation caused the overrepresentation of Tebi-SV1 and that the Tebi-PV1 repressor can interact with the Tebi-SV1 operator. The Tebi-SV1 operator sequence might be slightly longer (the repeat is longer), therefore, it remains to be experimentally tested whether the Tebi-SV1 repressor can also bind the Tebi-PV1 operator.

We do not agree that the conserved region 5' of the integrase operon is coincidental. We found that it is also conserved across other viruses (exemplified for LN27-SSV and SNJ2 in the manuscript now). Secondary structure prediction suggests very strong self-binding for all presented virus examples. This is now presented in Supplementary Figure 6. We conclude that this conserved region represents a 5'UTR that regulates transcription or translation of the integrase operon. We are aware that this has not yet been reported for archaeal viruses and will verify this in a follow up study.

Minor points related to new Figure 4:

1. I would suggest adding predicted TATA boxes and Bre elements (if such motifs are detectable - not necessarily conserved in haloarchaea or sufficient information is missing).

Thank you for pointing this out, as mentioned above we have now annotated TATA boxes and BRE elements.

2. It would be advisable to differentiate in the revised manuscript between mutations in the promoter and operator region (i.e. line 667). Since Fig 4 now nicely shows that the mutation is >55 bp upstream of the start codon and haloarchaeal transcripts are generally leaderless or have only very short leaders, the observed mutation it very unlikely in the (core) promoter region (i.e. mutated TATA/BRE), but as already suggested in the manuscript in the operator region.

As above, we now clearly differentiate between promotor regions and operator sequences, and indeed the mutation lies within the operator.

3. It would be advisable to check (i.e. with MEME suite) if the proposed operator region (around 58/-59) has any of the typical operator patterns i.e. palindrome or (inverted) repeats. This could potentially even further strengthen the claims.

Thank you very much for this advice, we used the MEME suite to identify the operator sequences and to show that the 5'UTR of the integrase operon is conserved across Tebi-SV1, Tebi-PV1. SSV-LN27 and SNJ2.

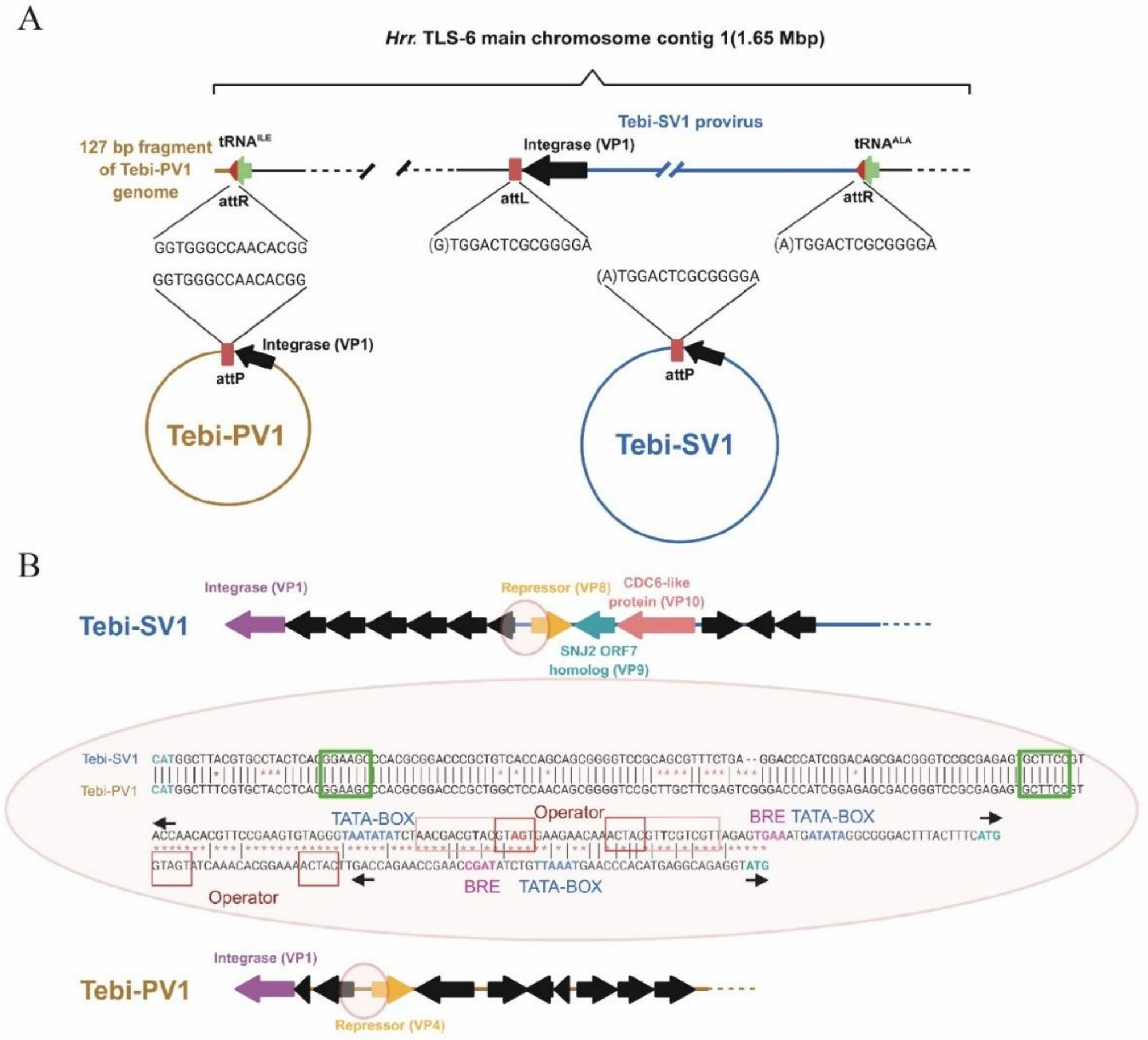

Figure 4: Schematic presentation of integration sites and promotor regions of Tebi-SV1 and Tebi-PV1. (A) Tebi-SV1 was found integrated into a contig of the main chromosome of *Hrr.*TLS-6. Attachment sites (attR, attL, and attP) are illustrated with the respective sequence. Tebi-PV1 was assembled as an extrachromosomal element. However, a 127 bp fragment upstream of the integrase was identical to the upstream end of the same contig and represent the integration site of Tebi-PV1. **(B)** Alignment of the regulatory region between the integrase operon and the repressor of Tebi-SV1 and Tebi-PV1. Start codons are highlighted in turquoise. The inverted repeats of each operator are conserved between Tebi-SV1 and Tebi-PV1 (highlighted with a dark red box). The operator of Tebi-SV1 may exceed the 5 bp repeat shared between Tebi-SV1 and Tebi-PV1 (light red box). Tebi-SV1 exhibits individual promotors (TATA-box and B recognition element, BRE) for the integrase operon and the repressor protein. For Tebi-PV1, we could only identify one TATA-box, that likely serves as promotor in both directions. Proposed transcription start sites and direction of transcription are indicated with arrows. The Tebi-SV1 genome retrieved from the first isolation, with Tebi-SV1 dominating, exhibits two SNPs within the operator sequence that are highlighted in red. Both viruses exhibit a conserved 5'UTR upstream of the integrase operon that is indicated by sequence similarity in the alignment. The 5'UTR has a high self-binding tendency, and the binding sites are highlighted with green boxes. Figure created with BioRender.com.

POINT-BY-POINT RESPONSES REVIEWER COMMENTS

Reviewer #2:

The author's response is very convincing to me.

The annotation of operator stes is an excellent addition that now clearly shows the shared operators. I now highly recommend the manuscript for publication.

Thank you very much for your time and your positive feedback.

One minor remark would be that the newly annotated BRE and TATA elements in Fig 4B have a very unusual distance to the respective start codons.

i.e. so close to the ATG that one would expect transcription to start downstream of the ATG. Since this would add more confusion than benefit, I would omit it in the proofing stage.

Indeed, the distances between TATA and transcriptional start site are usually longer, however, varying distances, in particular shorter distances, have been reported. For example: https://doi.org/10.1111/j.1365-2958.2005.04705.x, https://doi.org/10.3390/v13122388**). Nevertheless, it is very well possible that transcription starts elsewhere downstream than indicated in the figure.**

Since we have not experimentally verified BRE and TATA elements and transcription start sites, we now included 'proposed'/'potential' for start codons, BRE and TATA elements and indicate only transcriptional direction and not transcription start sites.

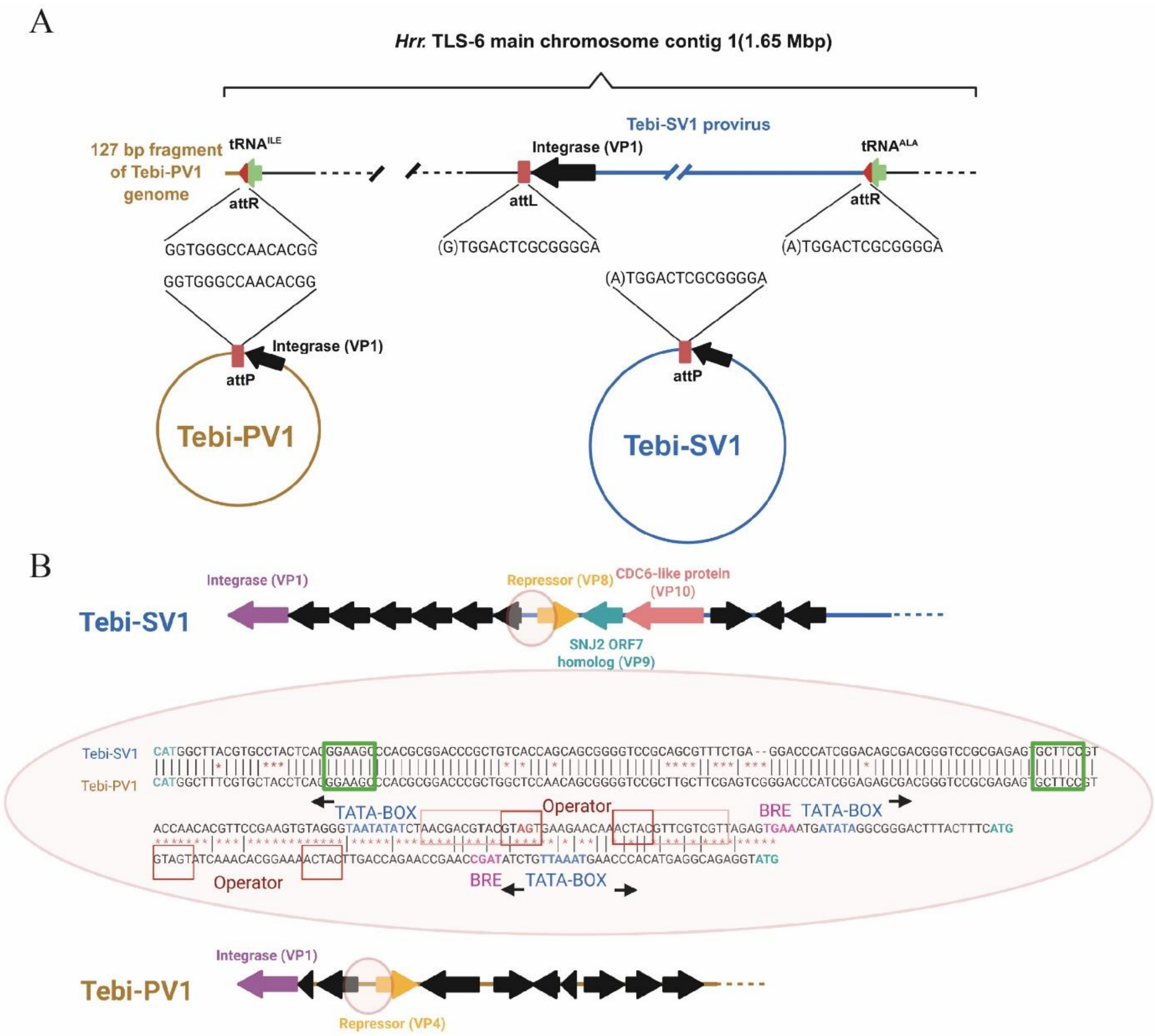

Figure 4: Schematic presentation of integration sites and promotor regions of Tebi-SV1 and Tebi-PV1. (A) Tebi-SV1 was found integrated into a contig of the main chromosome of *Hrr.*TLS-6. Attachment sites (attR, attL, and attP) are illustrated with the respective sequence. Tebi-PV1 was assembled as an extrachromosomal element. However, a 127 bp fragment upstream of the integrase was identical to the upstream end of the same contig and represent the integration site of Tebi-PV1. **(B)** Alignment of the regulatory region between the integrase operon and the repressor of Tebi-SV1 and Tebi-PV1. Proposed start codons are highlighted in turquoise. The inverted repeats of each operator are conserved between Tebi-SV1 and Tebi-PV1 (highlighted with a dark red box). The operator of Tebi-SV1 may exceed the 5 bp repeat shared between Tebi-SV1 and Tebi-PV1 (light red box). Tebi-SV1 exhibits proposed promotors (TATA-box and B recognition element, BRE) for each the integrase operon and the repressor protein. For Tebi-PV1, we could only identify one potential TATA-box, that likely serves as promotor in both directions. Proposed directions of transcription are indicated with arrows. The Tebi-SV1 genome retrieved from the first isolation, with Tebi-SV1 dominating, exhibits two SNPs within the operator sequence that are highlighted in red. Both viruses exhibit a conserved 5'UTR upstream of the integrase operon that is indicated by sequence similarity in the alignment. The 5'UTR has a high self-binding tendency, and the binding sites are highlighted with green boxes. Figure created with BioRender.com.
