# Supplementary material for: Two coinfecting archaeal viruses provide insights into virus-virus interactions
Source: Commun Biol. 2026 Sep 24;9:1250. doi: 10.1038/s42003-026-11061-7 (PMC13612827; doi:10.1038/s42003-026-11061-7)
Supplement: Supplementary file 2 [file 42003_2026_11061_MOESM2_ESM.pdf]

# Tale of Two viruses: Two coinfecting archaeal viruses provide insights into virus-virus interactions

## Supplementary Text

## Results

### *Both virus genomes were detected in a previous host genome assembly as integrated elements*

Both viral contigs (Tebi-SV1 and Tebi-PV1), assembled from gradient purified concentrated culture supernatant (Figure 1A, band 2), were compared to a genome assembly (see methods) of reads generated from a *Hrr.* TLS-6 culture in 2021 (SRR12122876, SRA)[1]. The larger contig (26.6 kbp, Tebi-SV1) was found to have 99 % nucleotide identity to a 26.6 kbp region of the largest contig from the host genome assembly (1.65 Mbp) (Figure 4A). Using the reads from 2021[1] the region had a mean coverage of ~ 550 in comparison to ~420 for the entire host genome contig. The smaller contig (15.4 kbp, Tebi-PV1) was found to have 100% nucleotide identity to a single circular contig in the *Hrr.* TLS-6 genome assembly. This 15.4 kpb contig had a higher coverage of ~2730 (using reads from *Hrr.* TLS-6 culture in 2021) compared to the average mean coverage ~ 416 of three largest host genome contigs likely representing the chromosome of *Hrr.* TLS-6 (3 contigs, total of 3.85 Mbp), indicative for an extrachromosomal element. Additionally, we also identified the first 127 bp of the Tebi-PV1 genome at the start of the same contig containing Tebi-SV1 in the genome assembly, indicating that integration does occur. The 5' end of these 127 bp represent the 3' end of an isoleucine-tRNA. Upstream of the Tebi-PV1 integrase we identified 15 bp that are identical to the 3' end of this isoleucine-tRNA on the host chromosome and suggest that this represents the viral attachment site (Figure 4A).

### *Isolated Tebi-SV1 particles from the first virus preparation exhibit a genomic difference to the integrated Tebi-SV1*

Genome comparison between the genome assembled from DNA isolated from the purified Tebi-SV1 particles and the host genome assembled from reads generated in 2021 (2021, SRR12122876, SRA) revealed two SNPs in the viral genome, a replacement of base G to A at position 4 and a deletion of base A at position 3248. To determine whether these changes are significant or due to assembly issues we mapped the reads generated from the viral preparation to the assembled host genome and the reads generated from the host culture (2021, SRR12122876, SRA) to the assembled virus genome. Host reads mapping onto the virus genome reveal that base A at position 3248 is present in the host genome with a frequency of 99.3 % (p-value: $1.6 \times 10^{-2133}$), the significance of SNP at position 4 is not judgable (low read coverage, due to missing host sequence). Virus read mapping to the host genome reveal that the

SNP at position 3248 (-A) affects only 34.6% of the reads (9,403 of 27,278), however, a second SNP was detected at position 3249 (deletion of a G) in 44.2 % (12,037 of 27,253). These two SNPs are located within an imperfect inverted repeat (one mismatch) right upstream of the promotor of VP8 and downstream of the TATA box for an upstream transcript including VP7. We propose this inverted repeat to represent the operator (repressor binding side). The replacement of G to A at position 4 was confirmed with 98.2 % of the reads. However, the SNP is right upstream of the 14 bp repeat (attL) and attR does exhibit an A at this position, so this does not represent an actual SNP (Figure 4A).

***Evidence for replication and virus particle production of Tebi-SV1 and Tebi-PV1 in different haloarchaea***

We tested the host range of Tebi-SV1 and Tebi-PV1 with seven different haloarchaeal strains in biological triplicates. To assess whether infection was successful we determined cell-associated virus genome copy numbers and free virus particles in culture supernatants by qPCR. Cell-associated virus genome copy numbers were determined by separating cells from free virus particles by centrifugation. To avoid carryover of free virus particles, the cells were washed twice with fresh media (see methods) before isolating DNA from the cell pellet and determining virus genome copy numbers by qPCR. Since this approach does not remove virus particles that are strongly bound to the cell surface, we cannot infer whether resulting virus genome copy number represent intracellular virus genomes (proving successful infection) or virus genomes from virus particles bound to the cell surface. To still confirm that infection was successful, we calculated the total number of virus genomes present in the 5 mL culture at 96 hours or 113 hours post infection. Virus numbers determined per ng of DNA by qPCR were multiplied by the total amount of DNA extracted from cells of 1.5 mL culture and virus particles isolated from the 1.5 mL culture supernatant. The average from three biological replicates was then multiplied by 4.67 to account for 7 mL total culture volume. The sum of virus number associated with cells and virus number in the supernatant (total virus number in infected culture) was then compared with the total number of virus genomes added to the cells initially (Supplementary Table 5). When the total virus number in the infected culture exceeded the virus genomes added initially, we concluded that virus infection was successful and the virus genome was replicated. We suggest the same for virus-host pairs where virus genome copy numbers were similar for the following reasons. Though we carefully processed samples, washing of cell pellets does lead to loss of cellular material (irritation of the cell pellet when removing the supernatant); filtration of culture supernatant for virus purification can retain virus particles on the filter (0.2 um filter); and DNA extraction with a commercial kit likely also leads to loss of DNA (inefficient binding to columns, inefficient elution from columns). Therefore, we suggest the total virus numbers determined by qPCR are likely significantly lower than the real number in the culture. Additionally, we calculated the initial virus number that was added to cultures by qPCR (plaque formation could not be achieved with any of the two viruses on tested strains), which cannot differentiate between genome copy numbers and active virus particles. Since virus particles can be compromised

during purification processes, we strongly suggest that initial virus numbers added as determined by qPCR largely exceed the number of intact infectious virus particles.

Virus replication is also inferred for host organisms where an increased number of virus particles was detected after cultures were diluted and allowed to continue growth. The total virus genome copy number in culture supernatants determined before dilution of the culture was compared with the total virus genome copy number in culture supernatants 84 or 96 hours after dilution, including the dilution factor. This showed the increase of virus particles in culture supernatants for four virus-host pairs, proving viral replication and virus particle production (Supplementary Table 5).

***Co-occurrence of Tebi-SV-like relatives and Pleolipoviruses in other haloarchaea***

When searching for Tebi-SV1-like relatives in public databases, we found that several Tebi-SV1-like elements co-occurred with pleolipovirus-like proviruses in the same host genomes (Supplementary Table 6). In five out of seven organisms containing genomes of Tebi-SV1 relatives, we could identify a complete pleolipovirus genome in the same host genome. However, these pleolipovirus genomes vary in length and do not all share a particular taxonomic affiliation at the genus level (Supplementary able 6). Therefore, co-occurrence could be coincidental considering that many haloarchaeal genomes contain pleolipoviruses.

***Protein composition of virus particles***

Protein analysis via SDS-PAGE of Tebi-SV1 enriched virus preparations appears very distinct from Tebi-PV1 enriched preparations (Supplementary Figure 10). Mass spectrometry (MS) was performed on both samples to determine the protein composition of the two virus particles (Supplementary table 7). Six proteins were detected for Tebi-SV1: VP17, VP19, VP24, VP27, VP30, and VP35. VP17 and VP24, both hypothetical proteins, contain predicted transmembrane domains. VP27 had the highest relative abundance of the six proteins detected (Supplementary table 7), but does not show similarity to other annotated proteins, and no confident structural model could be retrieved. VP30 was the second most abundant of the six viral proteins detected in the proteome. The N-terminal region of VP30 (Alphafold 3 model)[2] aligns with carbohydrate binding domains of various proteins, suggesting that VP30 could interact with carbohydrates. VP35, 151 amino acids in length, is the only protein detected that shares a similar hydrophobicity profile with capsid proteins of other spindle-shaped viruses (Supplementary Figure 11). The MCPs of all so far identified spindle-shaped viruses share structural similarity and are smaller than 200 amino acids in length[3,4]. To further investigate the putative role of VP35 as the potential major capsid protein of Tebi-SV1 we used the predicted tertiary structure for comparisons. It was shown that the MCP of spindle-shaped virus SMV1 is processed at the N-terminus [4]. Therefore, we gradually removed the N-terminal amino acids of VP35 and modelled the structure using Alphafold 3, with the removal of 43 aa resulting in the structure with the best score (pTM=0.64) (Supplementary Figure 11). Structural alignment using Dali, revealed minor similarities with the MCP

of APBV1, SMV1 and SSV19 (Z-scores 2.3, 3.7 and 3.1, respectively). Therefore, additional experiments are needed to verify whether it could be the MCP of Tebi-SV1.

For Tebi-PV1, four proteins were detected by proteomics (Supplementary Table 7). The spike protein VP12 was present in multiple bands of the SDS-PAGE. It was particularly enriched in the 95 kDa and 130-150 kDa bands (representing 28% and 33.8% of the total protein abundance, respectively). Additionally, an archaeal Type IV pilin-like protein (VP6), a putative NTPase (VP16), and a hypothetical protein (VP15) were detected (Supplementary Table 7). All detected proteins have homologs in other pleolipoviruses (Figure 1B), and were detected in pleolipovirus particles previously [5,6], except VP6, the archaeal T4P-like protein.

We also detected a number of host proteins in the samples, many of which were hypothetical proteins (Supplementary table 7). Annotated proteins detected included a significant number of membrane-associated proteins, such as components of ABC transporters, and other proteins previously detected in EVs of haloarchaea[7,8]. We suggest these host proteins derive from EVs and cellular appendages that co-purify with the virus particles, as also observed by TEM (Figure 7).

## Discussion

Nearly all spindle-shaped viruses described so far exclusively carry out chronic infections, where cell lysis does not occur [9–14]. One exception is the Acidianus two-tailed virus (ATV), which is described as causing cell lysis [15]. Highest titres of Tebi-SV1 were obtained from a culture grown from a single colony that showed a very strong growth inhibition. Subsequent cultivations of different colonies of the same strain or the original stock showed growth behaviour similar to other *Halorubrum* strains (Supplementary Figure 1), while producing only a small amount of spindle-shaped virus particles. With the lack of a virus-free strain that we could solely infect with Tebi-SV1, we were unable to judge the growth differences between an uninfected culture and a culture producing low amounts of Tebi-SV1. However, considering that only low titers of Tebi-SV1 are produced, the influence on the growth behaviour of the entire host population might be minor. Chronic virus infections rarely cause such significant growth deficiencies observed for the first culture producing high amounts of Tebi-SV1[6,16]. Therefore, we suggest that Tebi-SV1 may cause a lytic infection as described for ATV[15]. Supporting this, we were able to recover infectious Tebi-SV1 particles after mechanical lysis of the host cells. Approximately half of Tebi-SV1 virions recovered after removing the S-layer, suggesting that many particles accumulate on the cell surface or may be trapped between the S-layer and the cell membrane, an observation consistent with previous reports[10,17]. The remaining virions were isolated by spheroplast lysis, indicating that Tebi-SV1 virions may accumulate intracellularly. The isolation of infectious Tebi-PV1 particles from lysed spheroplasts was puzzling. Considering that pleolipoviruses are thought to be released by budding through the host cell membrane, we did not expect to isolate infectious virus particles by spheroplast lysis. We assume that Tebi-PV1 particles may have been attached to pili (Supplementary Figure 13), which are not affected by S-layer removal but by spheroplast lysis, or that

Tebi-PV1 particles might have been bound to a fraction of the S-layer that is membrane anchored and not removed by $MgCl_2$ depletion[18]. Alternatively, Tebi-PV1 genomes could be enclosed in spontaneously formed membrane vesicles upon cell lysis, which subsequently fuse with a host cell. Transformation of naked pleolipovirus genomic DNA was shown to be sufficient for successful infection[19]. While any of these mechanisms may also apply to Tebi-SV1, the Tebi-PV1:Tebi SV1 ratio was lower in spheroplast preparations than in virus preparations from culture supernatants and intact Tebi-SV1 particles were observed by TEM.

Several proteins detected in virions of Tebi-SV1 contain predicted transmembrane domains, and virus particles isolated from both the culture supernatant and the cell lysate were sensitive to chloroform treatment. Infectivity was still detected in contrast to His1, which is inactivated by chloroform[20]. We hypothesize that Tebi-SV1 particles may contain lipids or lipid-modified structural proteins, as reported for other spindle-shaped viruses[4,12–14,21,22]. However, chloroform sensitivity can also be due to an impact on hydrophobic protein-protein interactions, and lipids can be acquired both intracellular[23–25] or by budding through the host cell membrane[26], not allowing for a conclusion on the exit strategy of Tebi-SV1. In summary, there is strong evidence that Tebi-SV1 release has a notable effect on host growth. However, we cannot infer whether this growth inhibition is caused by cell lysis. Strong growth retardation has also been described for some chronic viruses[16], or could be caused by virus-induced cell death or dormancy[11,27].

**Supplementary Figures**

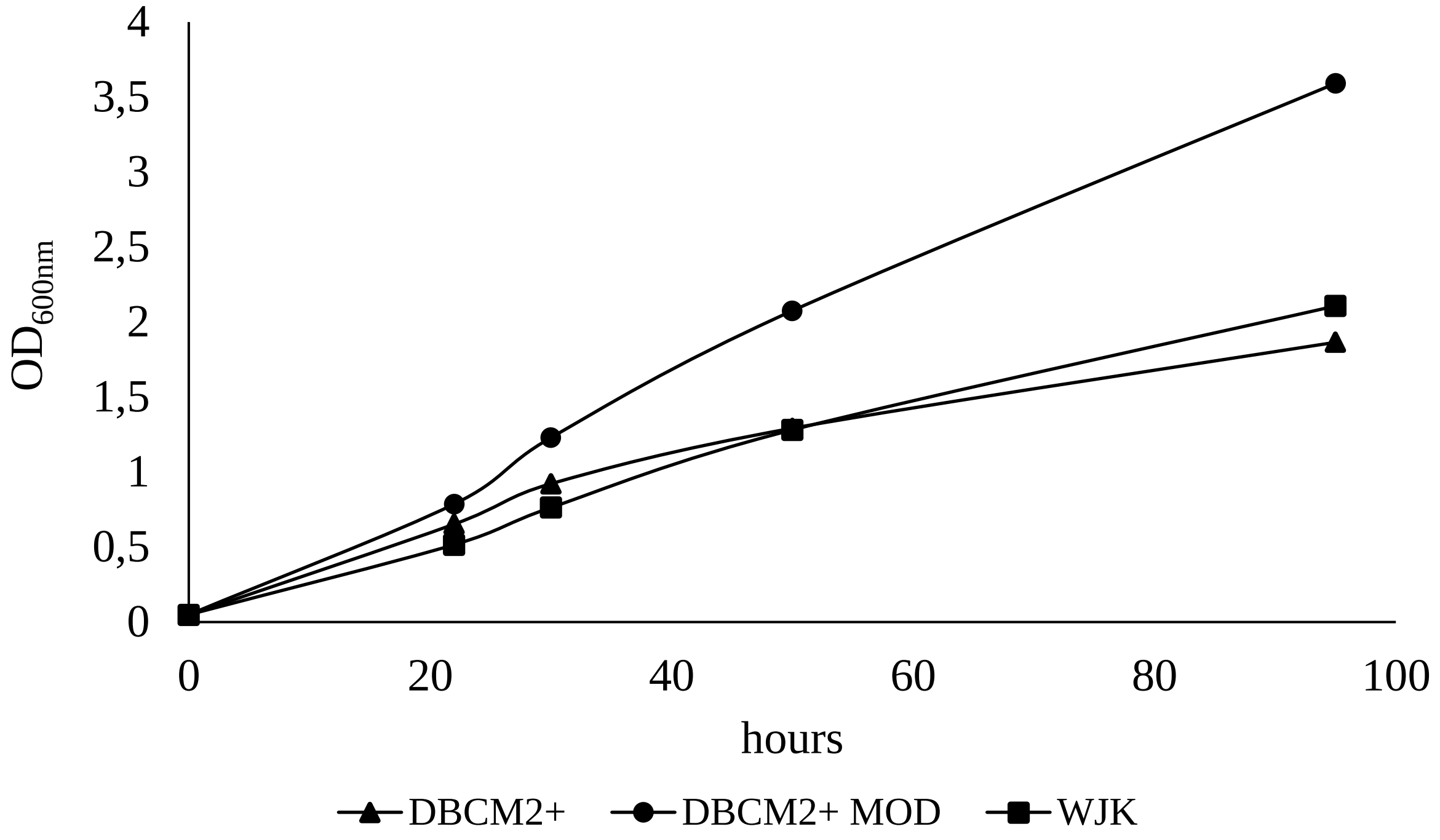


**Supplementary Figure 1: Representative example of growth behavior of *Hrr.* TLS6 in liquid media.** Growth curves of *Hrr*. TLS-6 in two different media (Methods). Growth was monitored by measuring optical density at 600 nm. Graphs represent on biological replicate for each condition.

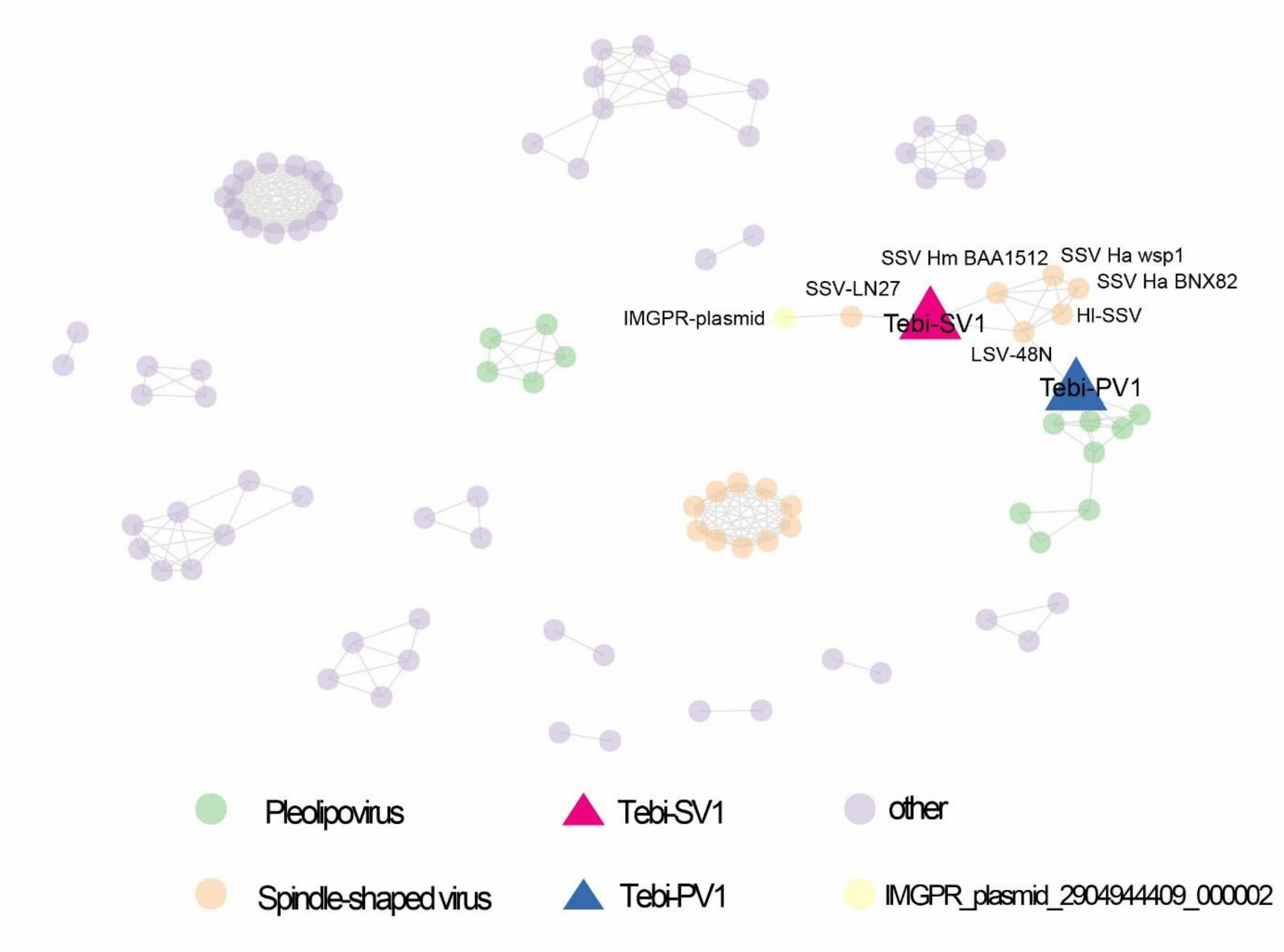


**Supplementary Figure 2: Gene sharing network between Tebi-SV1 and Tebi-PV1 and archaeal viruses and plasmids.** Circles signify genomes while lines represent shared protein content. Each circle represents a distinct entity. Triangles indicate the main viruses of interest (Tebi-SV1 and Tebi-PV1). Tebi-SV1 appears to belong to a distinct group with no similarity to other viruses than those who belong to proposed family, *Almyrocitroviridae*. Accession numbers of labelled genomes are presented in Supplementary Table 1.

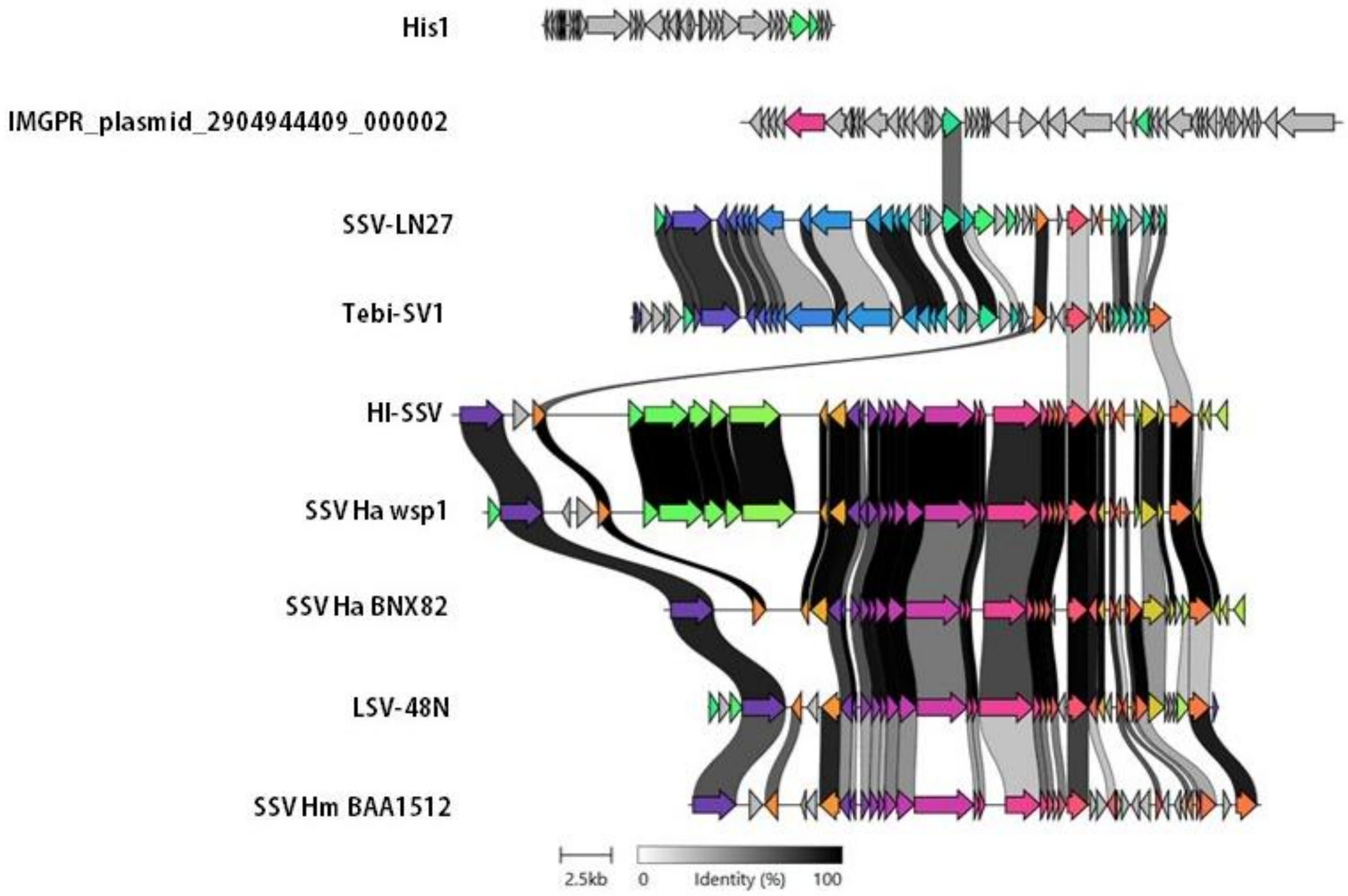


**Supplementary Figure 3: Genome comparison of putative spindle-shaped virus genomes** of Tebi-SV1-like elements identified from the IMGPR databases as well as previously described spindle-shaped viruses (His1, LSV-48N, and relatives). Homologous genes are shown in the same colors with percent identity represented vertically between genes.

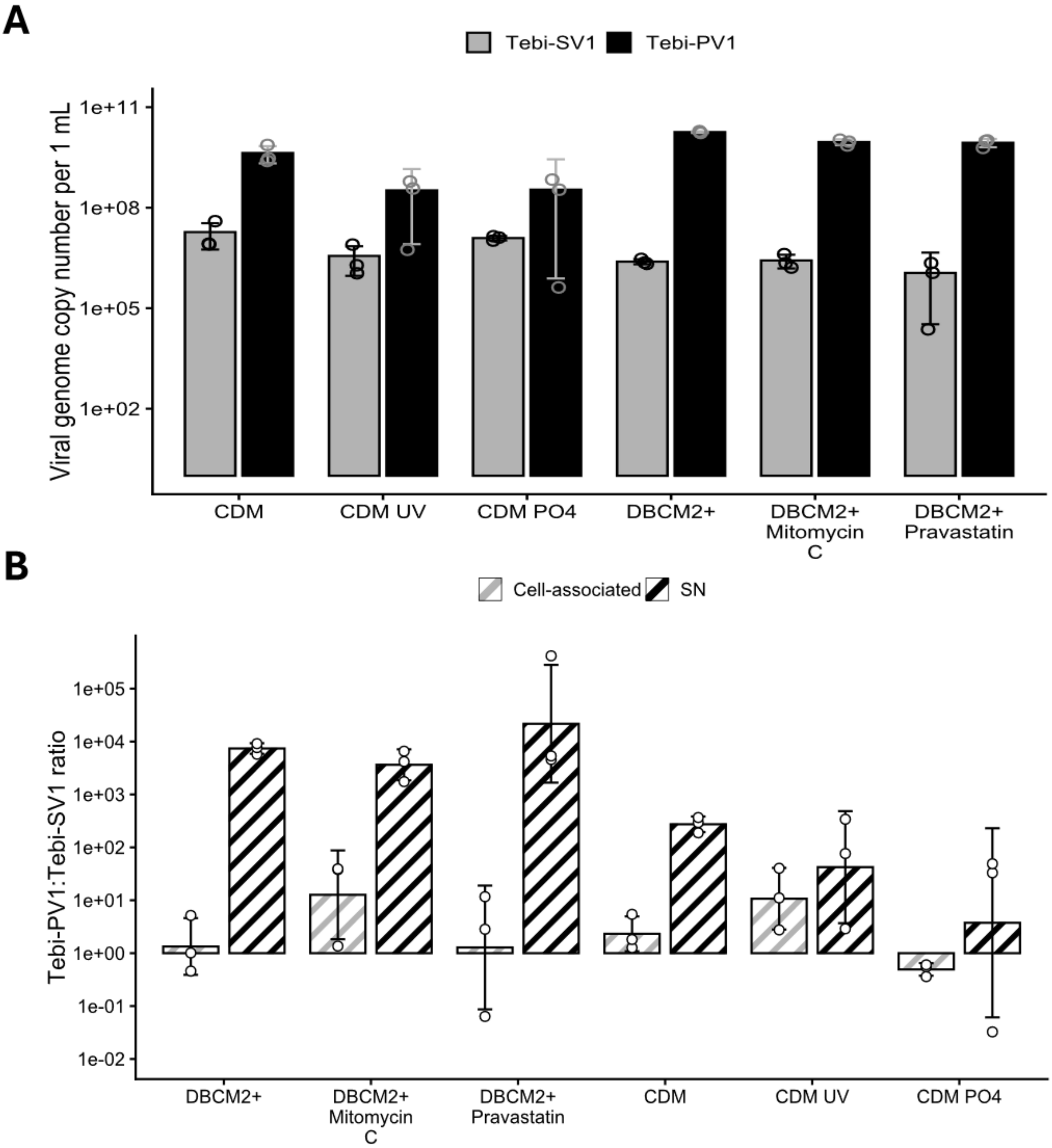


**Supplementary Figure 4: Determination of virus ratios under different growth conditions by qPCR. (A)** Viral genome copy number of Tebi-SV1 and Tebi-PV1 per ml supernatant was quantified from supernatant of *Hrr*. TLS-6 in differently treated cultures. **(B)** Intracellular viral genome copy number ratios (Tebi-PV1:Tebi-SV1) for (A). Mitomycin C and Pravastatin treatment was done in DBCM2+ medium. UV treatment and supplementation with $PO_4$ was done in CDM medium. (A and B) Bars represent the mean of n=3 biologically independent experiments with individual datapoints shown and the error bars represent the standard deviation of n=3 biologically independent experiments. Statistical analysis is summarized in Supplementary Table 3.

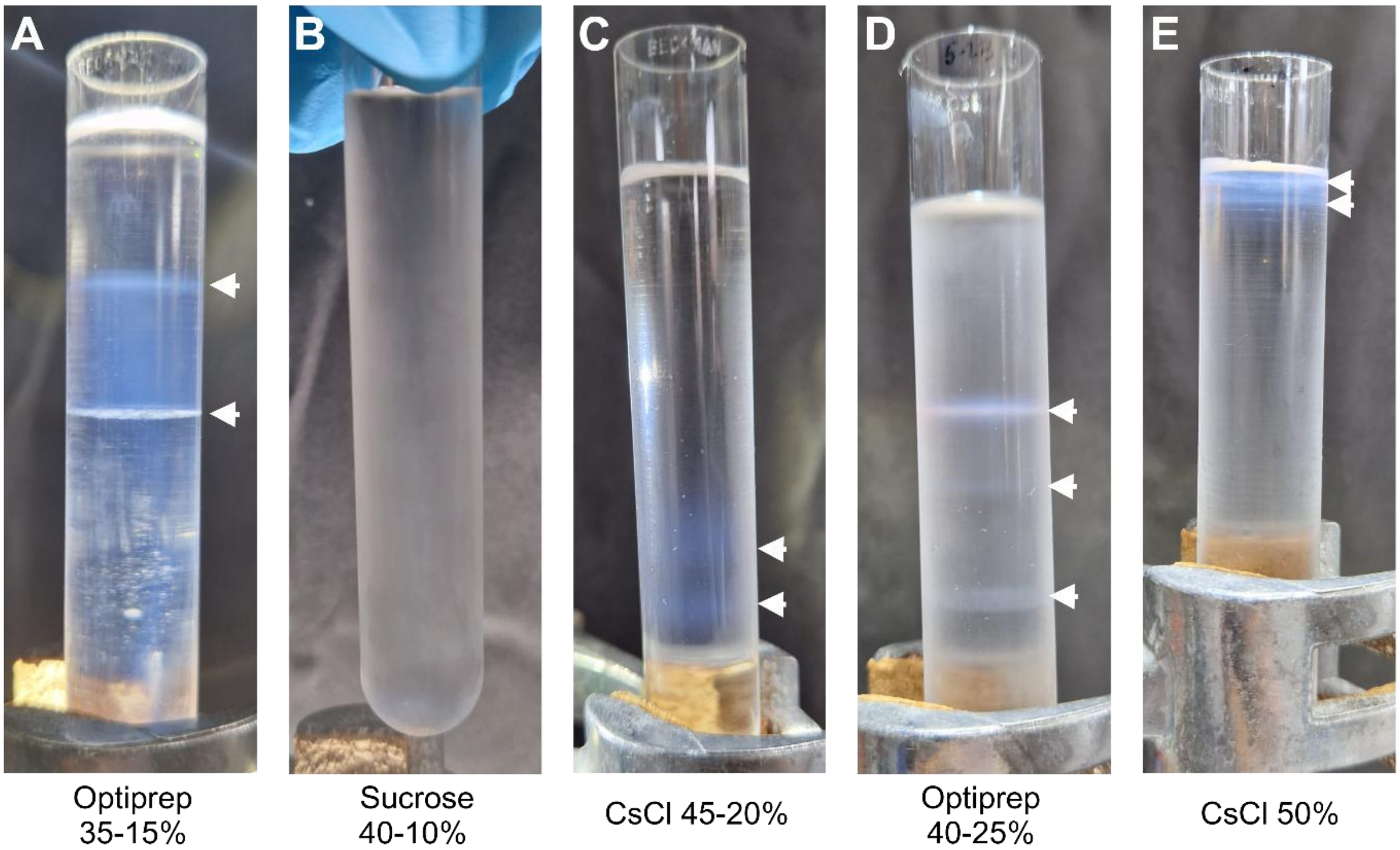


**Supplementary Figure 5: Separation of viruses *via* density gradients**. Separation of viruses on different density gradients (**A-E**). The gradient material and concentration are indicated below each gradient picture. All observed bands are indicated with a white arrow. All bands were screened by TEM and PCR for both Tebi-SV1 and Tebi-PV1 (Supplementary Data file 1: Supplementary Table 4).

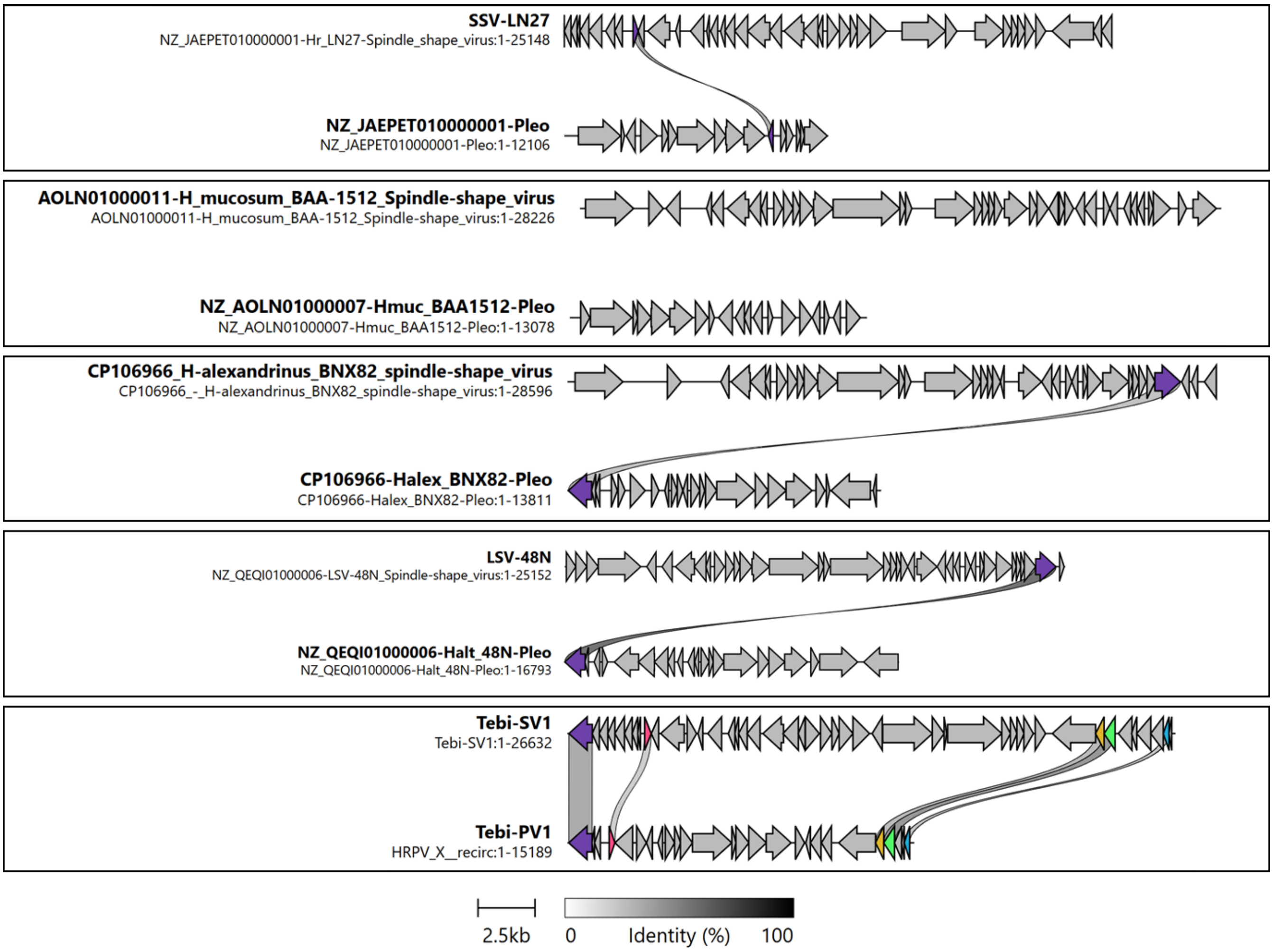


**Supplementary Figure 6: Genome comparison** of predicted pleolipoviruses within the same host genome of multiple predicted spindle-shaped viruses.

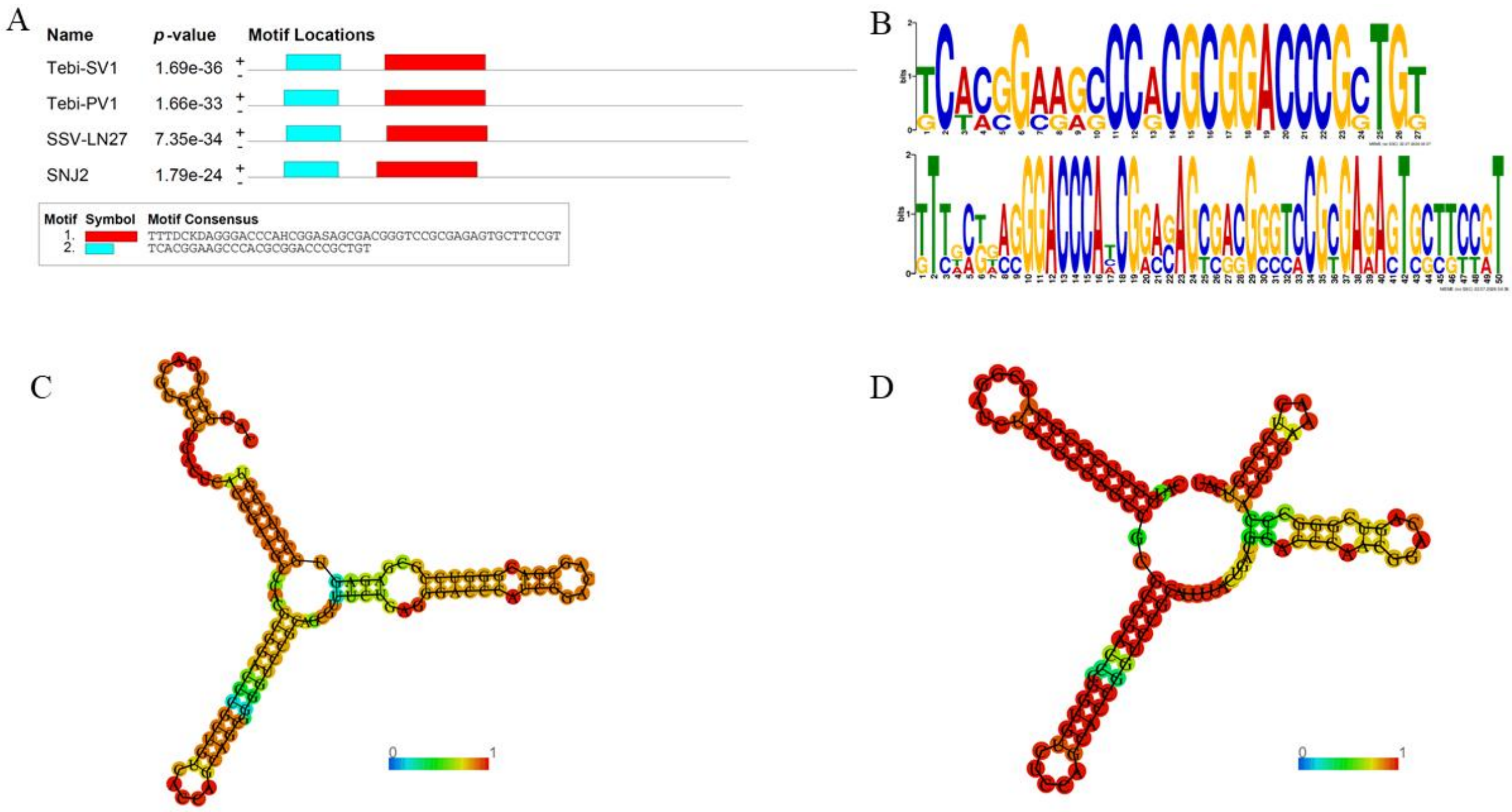


**Supplementary Figure 7: Conservation of the 5’UTR upstream of the integrase operon.** Motif location **(A)** and motif consensus sequence **(B)** for the 5’UTR of Tebi-SV1, Tebi-PV1, SSV-LN27 and SNJ2 as computed by MEME (default settings with minimum with of motif set to 20 bp) [28]. Secondary structure prediction of 5’UTR of Tebi-SV1 **(C)** and SNJ2 **(D)** using the RNAfold WebServer with default settings [29]. The colouring scheme illustrated base-pair probabilities.

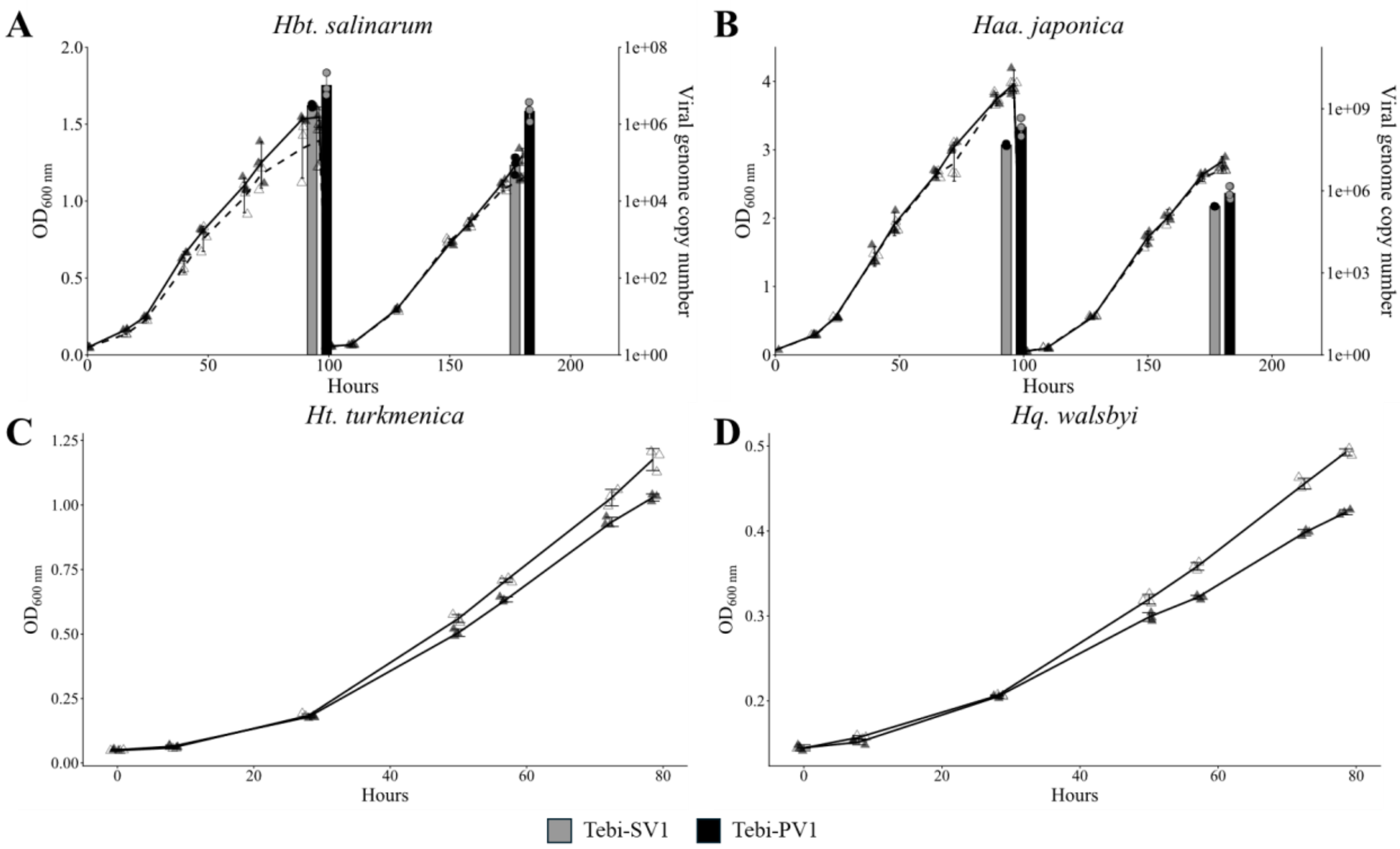


**Supplementary Figure 8:** Growth curves of infected (broken line) and uninfected (solid line) of four additionally tested host organisms **(A-D)**. After reaching stationary phase, cultures of *Hbt. salinarium* and *Haa. japonica* (A-B) were diluted to a starting $OD_{600nm}$ of 0.05 and allowed to regrow without additional virus added. The means of n=3 biologically independent experiments are shown as bars (viral genomes per 1.5 mL culture) and lines (for $OD_{600nm}$) with individual data points plotted and the error bars represent the standard deviation of n=3 biologically independent experiments in all curves. (A-B) Green bars represent the number of Tebi-SV1 genomes per 1.5 mL culture and blue bars represent Tebi-PV1 genomes per 1.5 mL culture detected in the supernatant at 96 and 180 hpi. Error bars represent the standard deviation of n=3 biologically independent experiments.

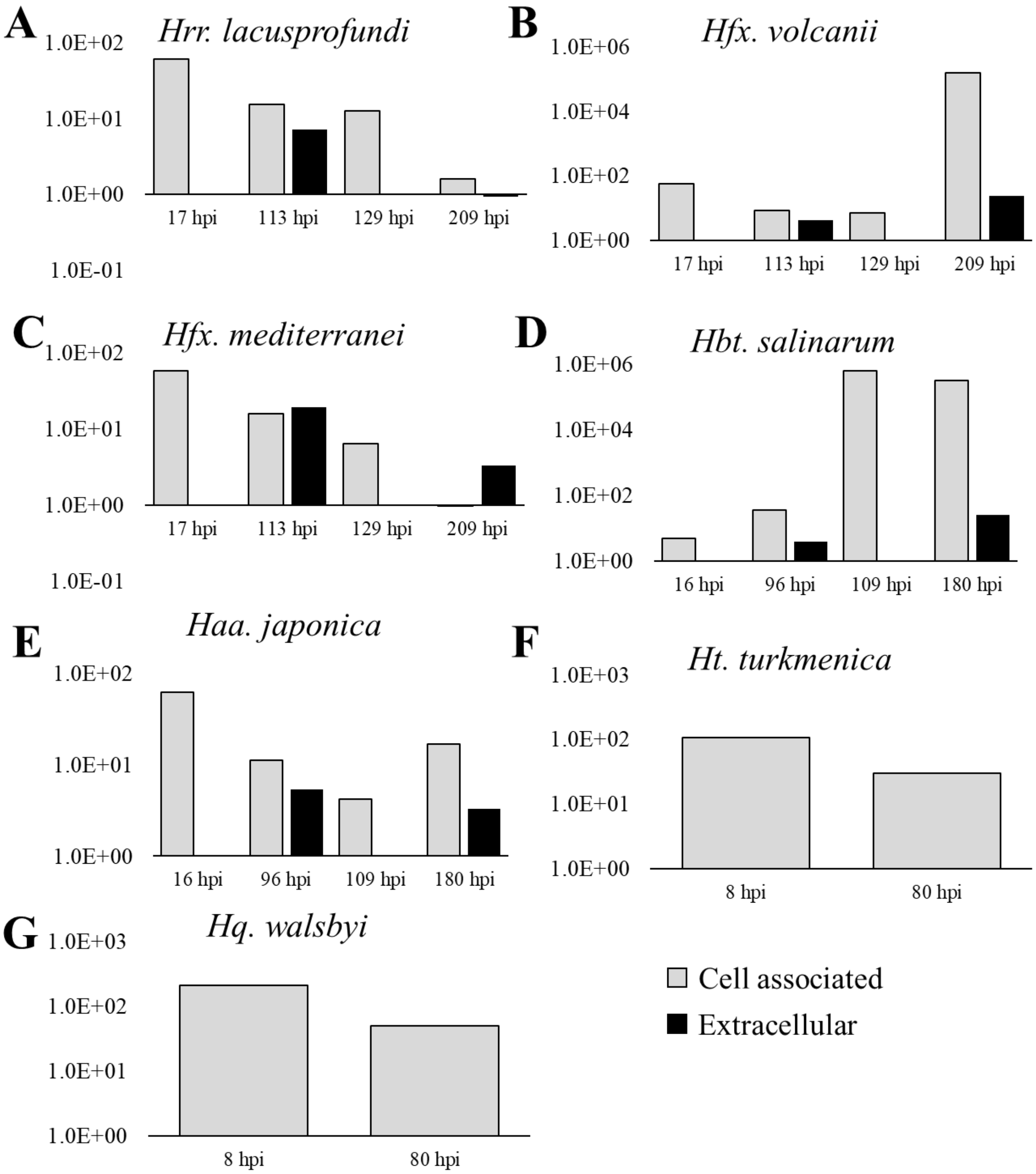


**Supplementary Figure 9: Tebi-PV1:Tebi-SV1 genome copy number ratios.** Cell-associated (grey) and extracellular (black) ratio of genome copy numbers of Tebi-PV1:Tebi-SV1 in seven different haloarchaeal organisms. **(A)** *Hrr lacusprofundi*. **(B)** *Hfx. volcannii*. **(C)** *Hfx. mediterranei*. **(D)** *Hbt. salinarium*. **(E)** *Haa. japonica*. **(F)** *Ht. turkmenica*. **(G)** *Hq. walsbyi*. The number of Tebi-SV1 genomes and Tebi-PV1 genomes were determined by qPCR in cells and the supernatant of 1.5 mL culture (in biological triplicates) and the ratio was determined by dividing the average of Tebi-PV1 numbers with the average of Tebi-SV1 numbers. A value below zero denotes that the number of Tebi-SV1 genomes detected exceeds those of Tebi-PV1. Intracellular Tebi-SV1 numbers became undetectable in *Hfx. volcanii* at 209 hpi (B), therefore, the bar shown only represents Tebi-PV1 numbers.

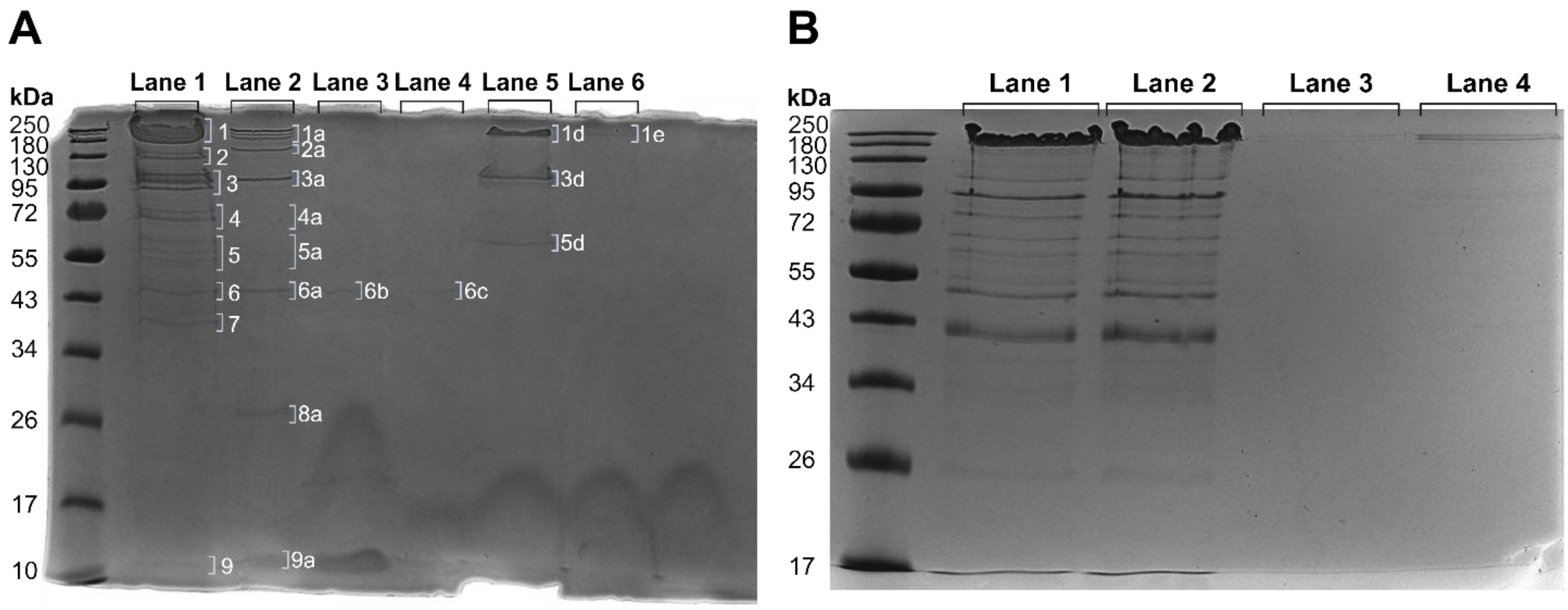


**Supplementary Figure 10: Precipitated viral proteins / Virus preparation from *Hrr*. TLS-6 cultures are visualized on 8% acrylamide SDS-PAGE gels. (A)** Lane 1 virus preparation from a culture supplemented with 1 mM $PO_4$ (no gradient purification). Lane 2 is band 2 of a CsCl gradient (Supplementary table 3). Lane 3 and 4 are bands 2 and 3 of an 40-25% Optiprep gradient (Supplementary table 3) from the unpurified sample of Lane 1. Lanes 4-6 are bands 1-3, respectively, of the first virus preparation from *Hrr*. TLS-6, that was sequenced (40-25% Optiprep gradient) (Supplementary table 3). Bands used for MS are labelled with a bracket and the corresponding sample ID found in Supplementary table 5. In **(B)**, Lanes 1 and 2 are biological duplicates of Tebi-PV1 enriched supernatant (no gradient purification) and lanes 3 and 4 are biological duplicates of TLS-6 cellular lysate grown in conditions to enrich for Tebi-SV1.

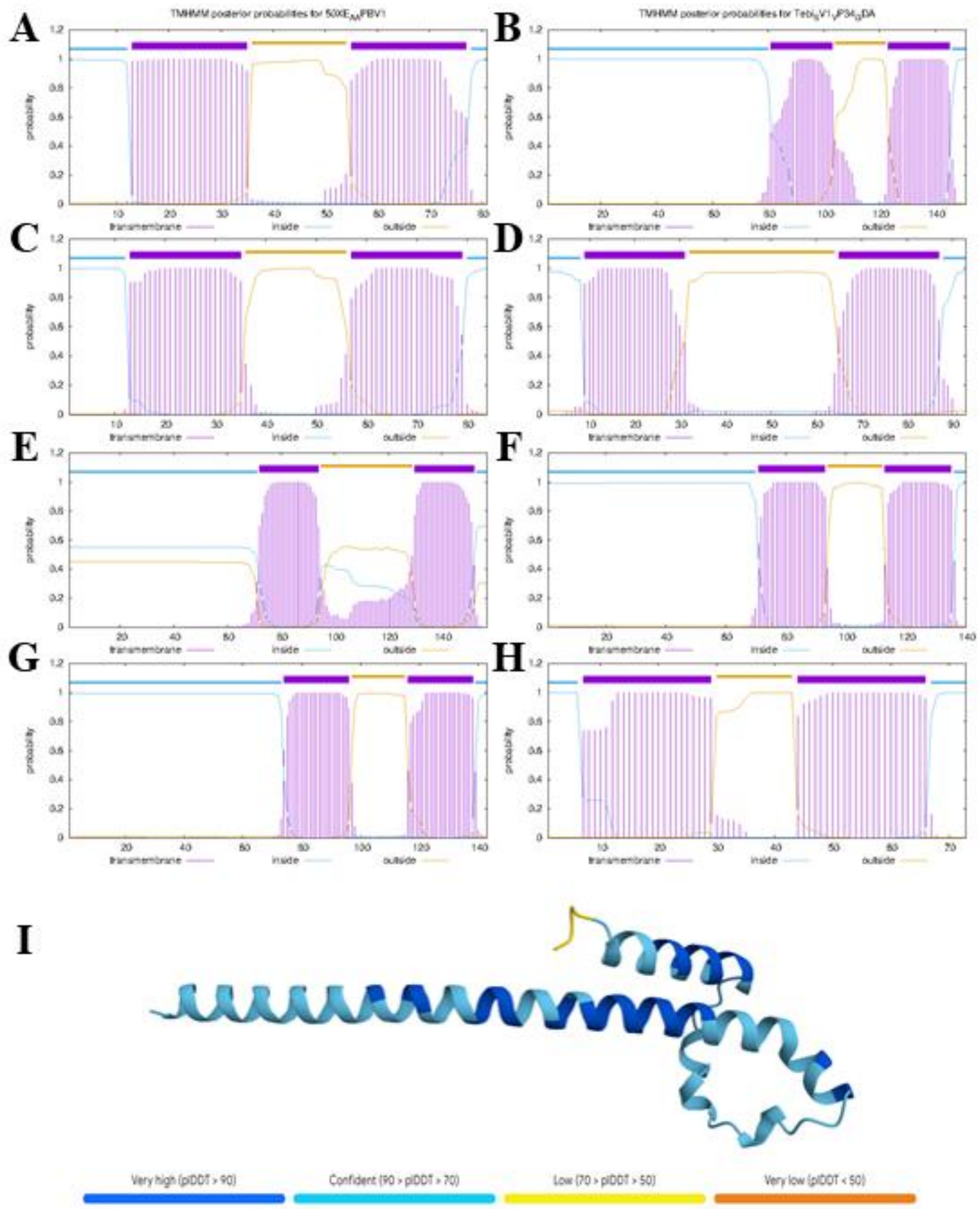


**Supplementary Figure 11: TMHMM predictions of transmembrane** probabilities using TMHMM server v2.0 [30,31] for the MCP sequences of spindle-shaped viruses. **(A)** Tebi-SV1 VP12, **(B)** Tebi-SV1 VP35, **(C)** His1 VP21, **(D)** SSV3 ORF2, **(E)** SMV1 7RO2_1, **(F)** SSV5 VP1, **(G)** ASV1 VP1, and **(H)** SSV19 7XDI_1. **(I)** Predicted tertiary structure (Alphafold 3 [2]) of VP35, with 43 aa removed from the N-terminus.

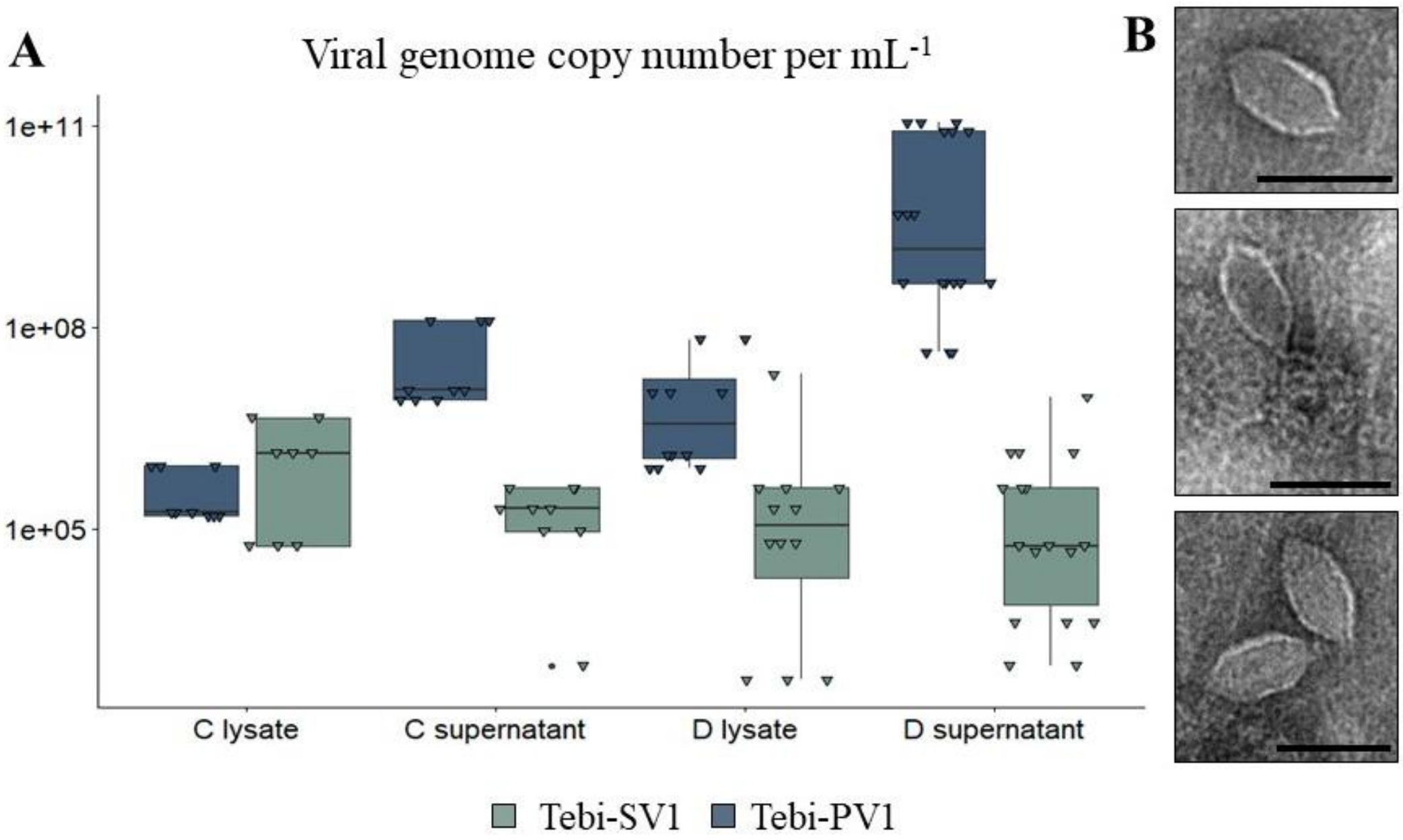


**Supplementary Figure 12: Virus particles can be isolated from cell lysates. (A)** Box plot of viral genome copy number per mL from lysed cells as well as from the supernatant of *Hrr*. TLS-6. D denotes "nutrient rich" medium, DBCM2+ and C denotes "nutrient deficient" medium, CDM supplemented with 1 mM $PO_4$. Single dots represent individual biological experiments, vertical line in the box represent the median of n=9 (C lysate and C supernatant), n=12 (D lysate), and n=18 (D supernatant), whiskers (horizontal lines) show smallest and largest observations. **(B)** Electron micrographs of Tebi-SV1 viruses in lysate preparations. Scale bars are 100 nm.

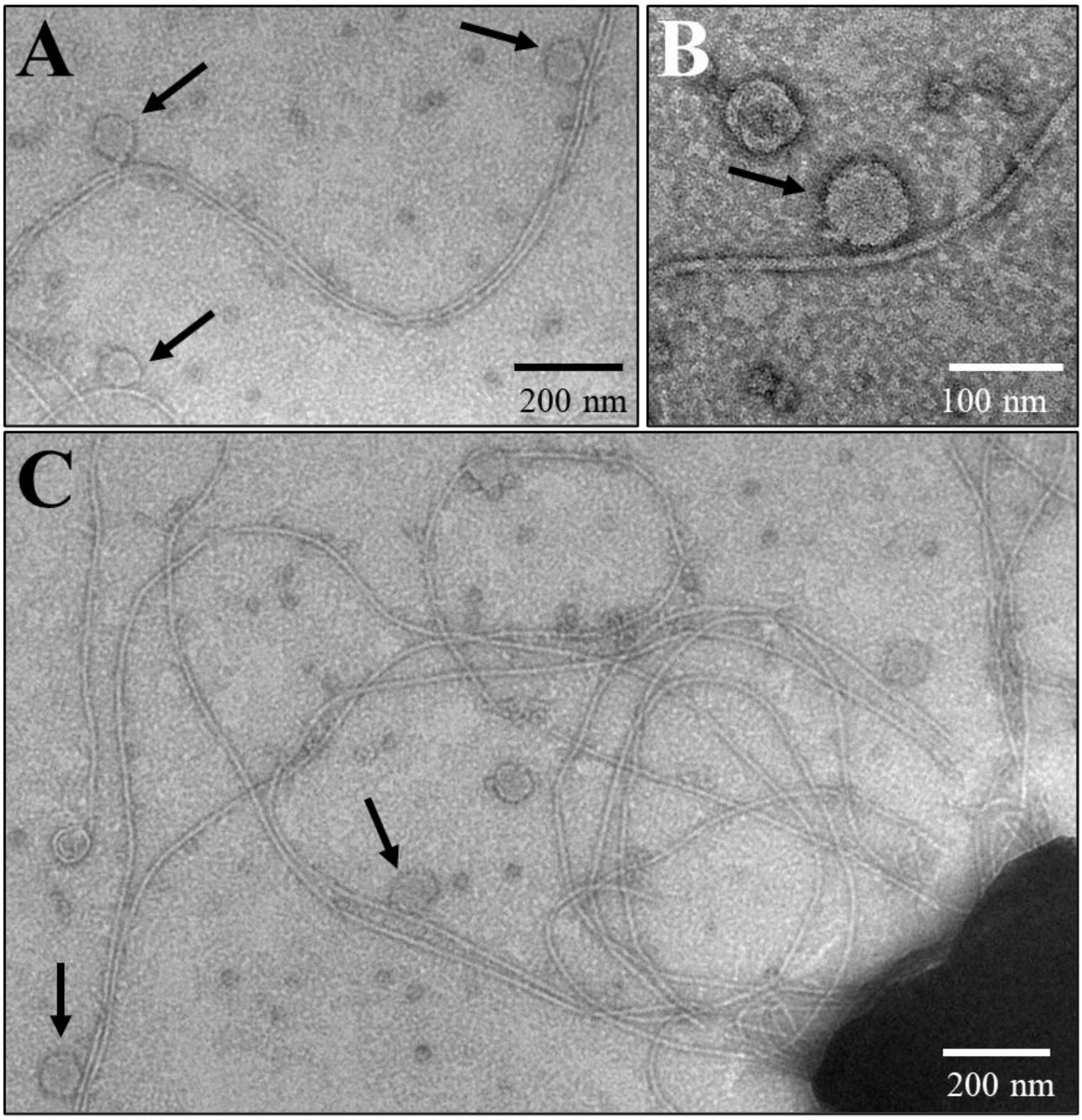


**Supplementary Figure 13**: Negatively stained micrographs of *Hrr.* TLS-6 cell cultures. **(A-C)** show Tebi-PV1 virions, denoted by black arrows, attached to cellular appendages.

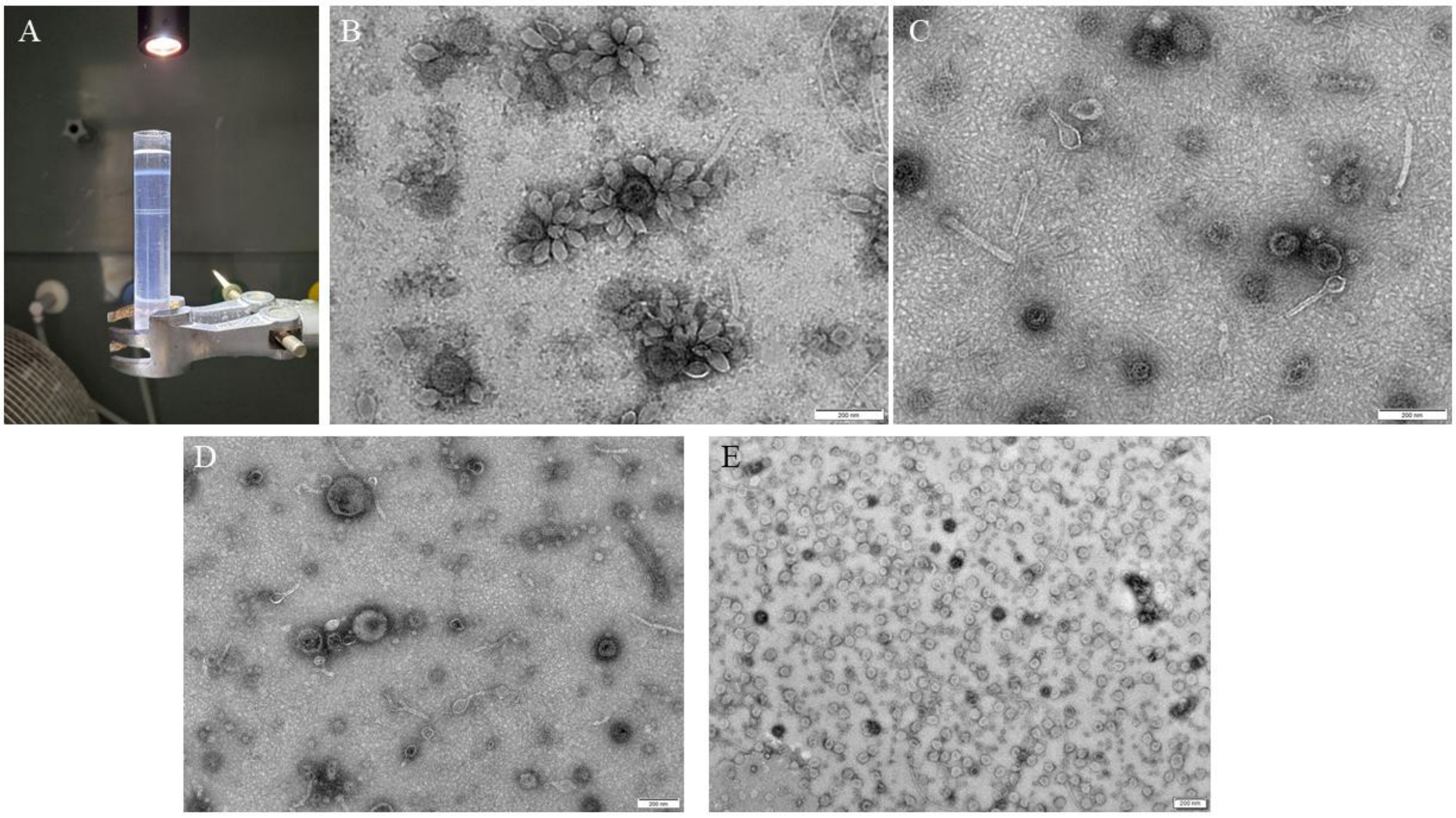


**Supplementary Figure 14:** Uncropped and unedited images Figure 1. **(A)** Figure 1A, **(B)** Figure 1B, **(C)** Figure 1C, **(D)** Figure 1D, **(E)** Figure 1E

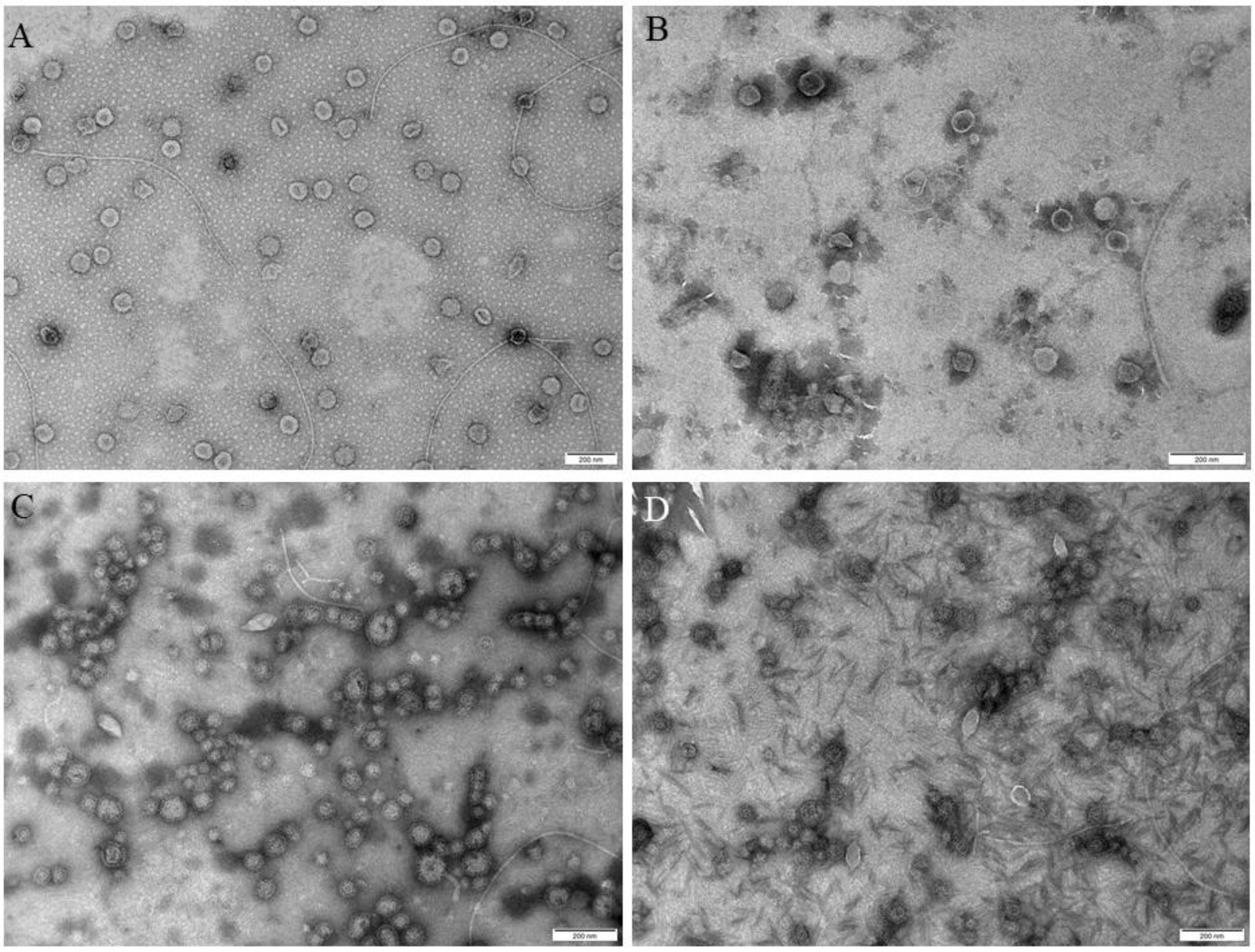


**Supplementary Figure 15:** Uncropped and unedited images Figure 5. **(A)** Figure 5E, **(B)** Figure 5F, **(C)** Figure 5G, **(D)** Figure 5H

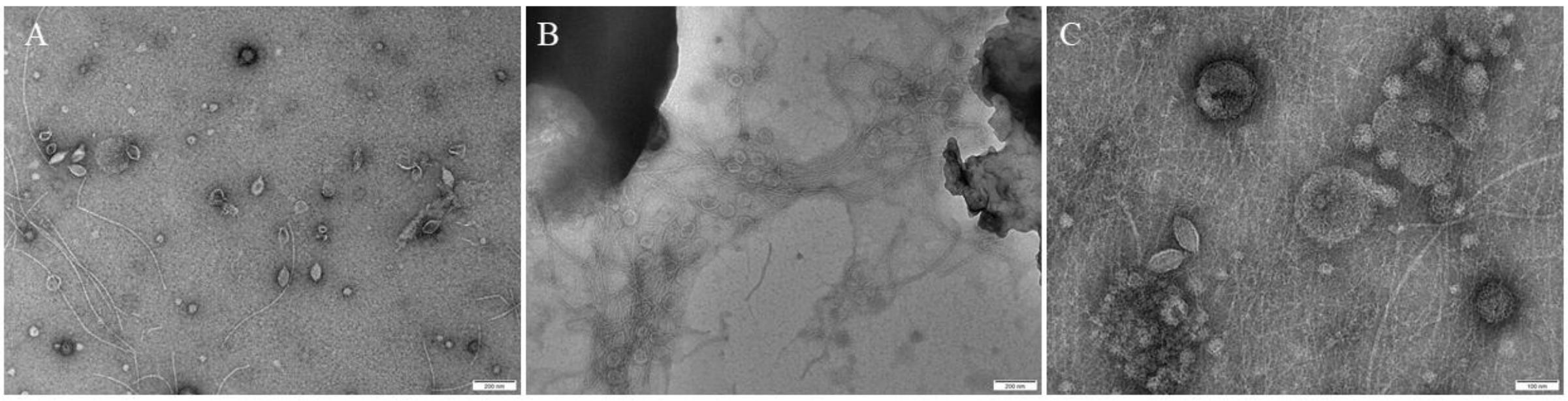


**Supplementary Figure 16:** Uncropped and unedited images Figure 7. **(A)** Figure 7C, **(B)** Figure 7D, **(C)** Figure 7E

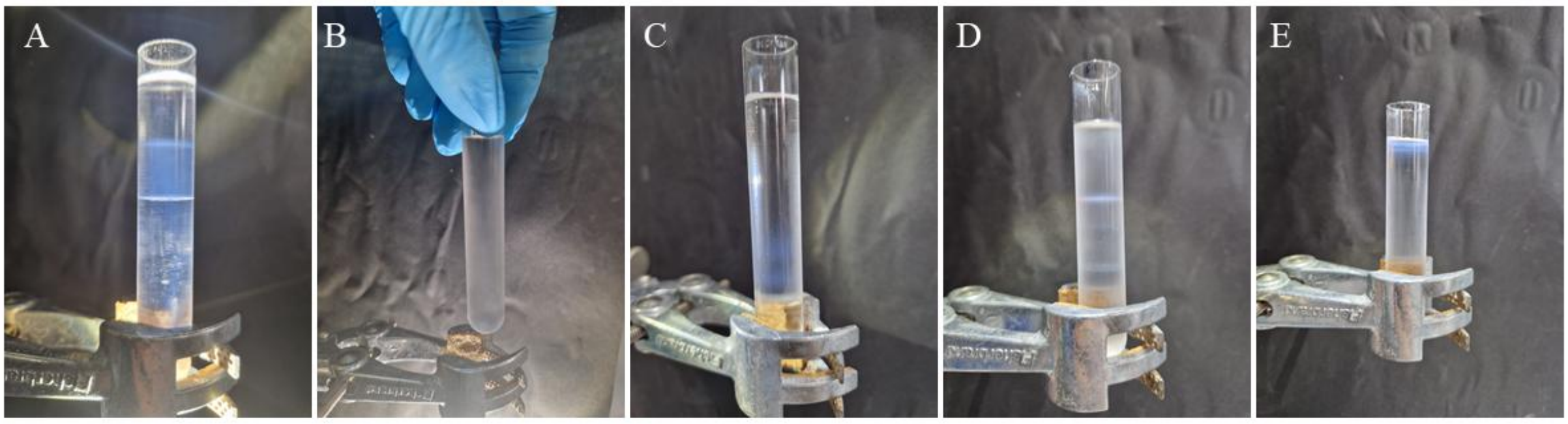


**Supplementary Figure 17:** Uncropped and unedited images Supplementary Figure 5. **(A)** Supplementary Figure 5A, **(B)** Supplementary Figure 5B, **(C)** Supplementary Figure 5C, **(D)** Supplementary Figure 5D, **(E)** Supplementary Figure 5E

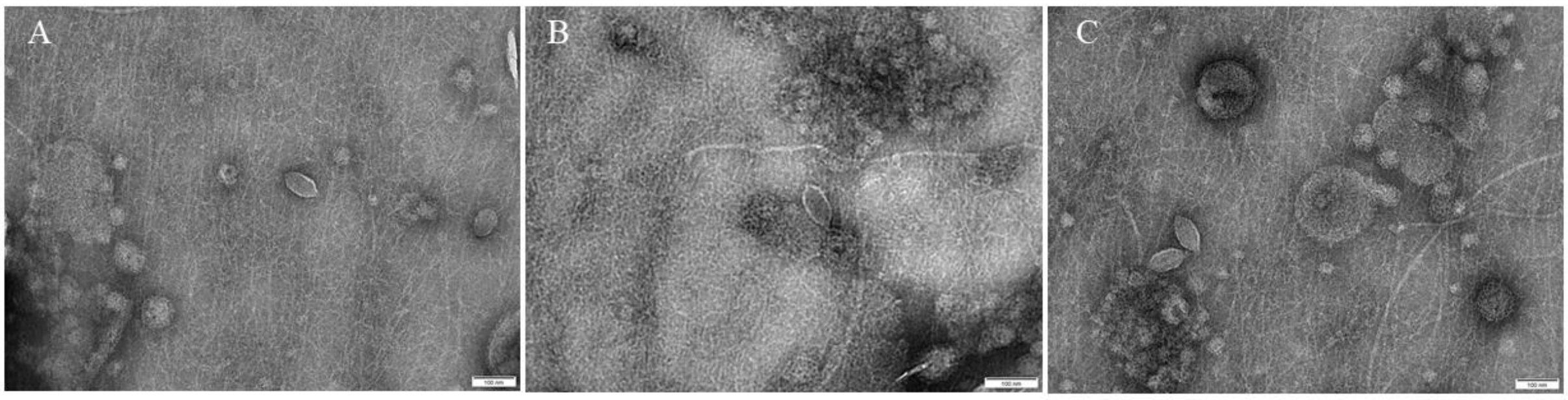


**Supplementary Figure 18:** Uncropped and unedited images Supplementary Figure 12. **(A-C)** Supplementary Figure 12B

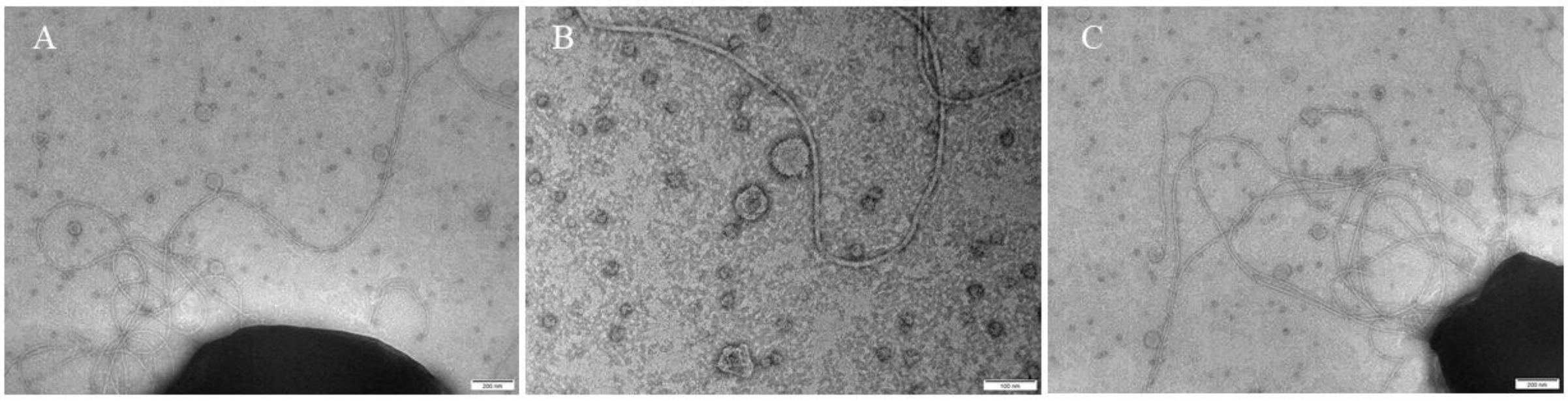


**Supplementary Figure 19:** Uncropped and unedited images Supplementary Figure 13. **(A)** Supplementary Figure 13A, **(B)** Supplementary Figure 13B, **(C)** Supplementary Figure 13C

## Supplementary Tables

**Supplementary Table 1:** Accession numbers for genomes included in genome-based phylogeny classification analyses (Figure 3) and Gene sharing network analysis (Supplementary Figure 2). The table is included in Supplementary Data file 1 (.xlsx).

**Supplementary Table 2:** Clustering results of Tebi-SV1 and relatives along with other known and unrelated spindle-shaped viruses. The cluster table was reconciled with Victor analysis to indicate the acceptable thresholds of gene-sharing at approximate genus level. The average nucleotide identity (ANI) threshold (95%) used to delineate viral species. The table is included in Supplementary Data file 1 (.xlsx).

**Supplementary Table 3:** Statistical comparison of data presented in Supplementary Figure 4. Statistical significance is indicated with green for p-value ≤ 0.05 (significant) and red for p-value > 0.05 (not significant).

**p-value intracellular Tebi-SV1**

| | DBCM2+ Mitomycin C | DBCM2+ Pravastatin | CDM | CDM + UV | CDM + PO4 |
|---|---|---|---|---|---|
| DBCM2+ | 3.28E-01 | 3.79E-05 | 7.72E-09 | | |
| CDM | | | | 4.15E-02 | 9.07E-03 |

**p-value intracellular Tebi-PV1**

| | DBCM2+ Mitomycin C | DBCM2+ Pravastatin | CDM | CDM + UV | CDM + PO4 |
|---|---|---|---|---|---|
| DBCM2+ | 5.82E-03 | 2.93E-03 | 2.69E-03 | | |
| CDM | | | | 1.42E-06 | 3.29E-01 |

**p-value extracellular Tebi-SV1**

| | DBCM2+ Mitomycin C | DBCM2+ Pravastatin | CDM | CDM + UV | CDM + PO4 |
|---|---|---|---|---|---|
| DBCM2+ | 4.93E-03 | 8.75E-01 | 4.56E-03 | | |
| CDM | | | | 2.78E-03 | 9.40E-14 |

**p-value extracellular Tebi-PV1**

| | DBCM2+ Mitomycin C | DBCM2+ Pravastatin | CDM | CDM + UV | CDM + PO4 |
|---|---|---|---|---|---|
| DBCM2+ | 4.57E-01 | 4.00E-03 | 3.10E-04 | | |
| CDM | | | | 9.02E-17 | 1.35E-09 |

**Supplementary Table 4:** Conditions and results from various density gradients as shown in Supplementary figure 2. Each band was observed by electron microscopy. Additionally, each band was DNase treated and the presence or absence of Tebi-SV1 and Tebi-PV1 genomes was tested by PCR. The table is included in Supplementary Data file 1 (.xlsx)

**Supplementary Table 5:** Host range assessment. Total numbers of virus genome copy numbers added to different host organisms compared to total number of virus genome copy numbers detected in 50 ml culture after specified times post infection. Comparison of total number of virus genomes detected in culture supernatants at different time points. The table is included in Supplementary Data file 1 (.xlsx)

**Supplementary Table 6:** Presence and absence of Pleolipovirus-like proviruses integrated into host genomes where Tebi-SV1 like relatives were identified. Where the absence is indicated by red writing,

and genomes with Pleolipoviruses present were described in green text. The table is included in Supplementary Data file 1 (.xlsx)

**Supplementary Table 7:** Mass spectrometry data for Tebi-SV1 and Tebi-PV1 particles. Proteome analysis of bands in an SDS-PAGE of virus particles as shown in Supplementary figure 8A. The table is included in Supplementary Data file 1 (.xlsx)

**Supplementary Table 8:** All host strains were screened for virus defense systems detected using PADLOC and CRISPRDetect. The table is included in Supplementary Data file 1 (.xlsx)

**Supplementary Table 9:** Host strains used to find virus-free host as well as determine the host range breadth of Tebi-SV1 and Tebi-PV1.

| **Strain ID** | **Reference** | **Medium** | **T (°C)** |
|---|---|---|---|
| *Halorubrum sp.* TLS-6 | (Saona et al., 2021) | DBCM2 modified (Mercier et al., 2023) and CDM modified (Nuttall et al., 2008) | 28 |
| *Halorubrum lacusprofundi* ACAM34_UNSW | (Mercier et al., 2023) | DBCM2 modified (Mercier et al., 2023) | 28 |
| *Halobacterium salinarum* 91-R6 | (Pfeiffer et al., 2019) | HS media (Pfeiffer et al., 2019) | 28 |
| *Haloferax mediterranei* | (Yang et al., 2015) | HV YPC (Nuttall et al., 2008) | 28 |
| *Haloferax volcanii* DS2 | (Hartman et al., 2010) | HV YPC (Nuttall et al., 2008) | 28 |
| *Haloarcula japonica* TR-1 | (Horikoshi et al., 1993) | HV YPC (Nuttall et al., 2008) | 28 |
| *Haloterrigena turkmenica type strain (4kT)* | (Saunders et al., 2010) | HV YPC (Nuttall et al., 2008) | 28 |
| *Haloquadratum walsbyi* | (Burns et al., 2007) | Leibniz Institut DSMZ-Deutsche Sammlung von Mikroorganismen und Zellkulturen GmbH ; Curators of the DSMZ; DSM 16854 | 28 |

**Supplementary Table 10:** All qPCR and PCR primer sequences used in this study with annealing temperature (T °C). Dimer formation was checked using OligoAnalyser (Inegrated DNA Technologies). All primer pairs were mapped to the relevant genome with a 5 bp mismatch allowance to ensure no unspecific binding would occur. All qPCR data had an efficiency between 90-110 % with an r2-value of >0.95.

| Description | Sequence 5' to 3' | Direction | Product Length (bp) | T (°C) | Concentration | Purpose | Type |
|---|---|---|---|---|---|---|---|
| Tebi-SV1 (qP55-56) | TGTTGTACTACCTCACATC<br>GAACTACTCACACTAACCA | Forward<br>Reverse | 89 | 59 | 100 nM | virus quantification | qPCR |
| Tebi-PV1 (qP59-60) | CTACTAACTTCTGGCTAACT<br>CGAGTCTAGGTAGTTGTAGT | Forward<br>Reverse | 80 | 61 | 100 nM | virus quantification | qPCR |
| *Hrr. lacusprofundi* main chromosome | GAGTTAGTGAAGTATCTTCG<br>GCTCTACATCCTCATAATAC | Forward<br>Reverse | 108 | 65 | 100 nM | host chromosome quantification | qPCR |
| *Hfx. volcanii* main chromosome polymerase | CCCGAATCAGGACGAAGAAC<br>ATTTGAGGTGCTCGGAGAAC | Forward<br>Reverse | 74 | 68 | 200 nM | host chromosome quantification | qPCR |
| *Hfx. mediterranei* DNA polymerase | GAAATAAGCGCTGCCTGGAC<br>GCGTCAAGCACAACATCTC | Forward<br>Reverse | 94 | 65 | 100 nM | host chromosome quantification | qPCR |
| Tebi-SV1 | CTCGGTCTCTCGGATGAAGC<br>GTACTCGTGGATCGTCCGAC | Forward<br>Reverse | 853 | 68 | 10 µM | virus detection | PCR |
| Tebi-PV1 | GCGGCAAACTACCAAGCAAA<br>GGAAATCGCATTGAGACCGC | Forward<br>Reverse | 1420 | 68 | 10 µM | virus detection | PCR |
| 16S Primer universal (Uni16S) | CCTACGGGNBGCASCAG<br>GACTACNVGGGTATCTAATCC | Forward<br>Reverse | 400 | 62 | 10 µM | host contamination detection | PCR |

## Supplementary References


1. Saona, L. A. *et al.* Phosphate-Arsenic Interactions in Halophilic Microorganisms of the Microbial Mat from Laguna Tebenquiche: from the Microenvironment to the Genomes. *Microbial Ecology* https://doi.org/10.1007/s00248-020-01673-9 (2021) doi:10.1007/s00248-020-01673-9.
2. Abramson, J. *et al.* Accurate structure prediction of biomolecular interactions with AlphaFold 3. *Nature* **630**, 493–500 (2024).
3. Ni, Y., Xu, T., Yan, S., Chen, L. & Wang, Y. Hiding in plain sight: The discovery of complete genomes of 11 hypothetical spindle-shaped viruses that putatively infect mesophilic ammonia-oxidizing archaea. *Environmental Microbiology Reports* **16**, e13230 (2024).
4. Wang, F. *et al.* Spindle-shaped archaeal viruses evolved from rod-shaped ancestors to package a larger genome. *Cell* 1–11 (2022) doi:10.1016/j.cell.2022.02.019.
5. Atanasova, N. S., Demina, T. A., Krishnam Rajan Shanthi, S. N. V., Oksanen, H. M. & Bamford, D. H. Extremely halophilic pleomorphic archaeal virus HRPV9 extends the diversity of pleolipoviruses with integrases. *Research in Microbiology* **169**, 500–504 (2018).
6. Pietila, M. K. *et al.* Virion Architecture Unifies Globally Distributed Pleolipoviruses Infecting Halophilic Archaea. *Journal of Virology* **86**, 5067–5079 (2012).
7. Erdmann, S., Tschitschko, B., Zhong, L., Raftery, M. J. & Cavicchioli, R. A plasmid from an Antarctic haloarchaeon uses specialized membrane vesicles to disseminate and infect plasmid-free cells. *Nature Microbiology* **2**, 1446–1455 (2017).
8. Mills, J. *et al.* Extracellular vesicle formation in Euryarchaeota is driven by a small GTPase. *Proceedings of the National Academy of Sciences* **121**, e2311321121 (2024).
9. Gorlas, A., Krupovic, M., Forterre, P. & Geslin, C. Living side by side with a virus: Characterization of two novel plasmids from Thermococcus prieurii, a host for the spindle-shaped virus TPV1. *Applied and Environmental Microbiology* **79**, 3822–3828 (2013).
10. Jong-Geol Kima *et al.* Spindle-shaped viruses infect marine ammoniaoxidizing thaumarchaea. *PNAS* **116**, 15645–15650 (2019).
11. Dewerff, S. J., Zhang, C., Schneider, J. & Whitaker, R. J. Intraspecific antagonism through viral toxin encoded by chronic Sulfolobus spindle-shaped virus. *Philosophical Transactions of the Royal Society B: Biological Sciences* **377**, (2022).
12. Pietilä, M. K., Atanasova, N. S., Oksanen, H. M. & Bamford, D. H. Modified coat protein forms the flexible spindle-shaped virion of haloarchaeal virus His1. *Environmental Microbiology* **15**, 1674–1686 (2013).
13. Uldahl, K. B. *et al.* Life Cycle Characterization of Sulfolobus Monocaudavirus 1, an Extremophilic Spindle-Shaped Virus with Extracellular Tail Development. *Journal of Virology* **90**, 5693–5699 (2016).
14. Xiang, X. *et al.* Sulfolobus tengchongensis Spindle-Shaped Virus STSV1: Virus-Host Interactions and Genomic Features. *J Virol* **79**, 8677–8686 (2005).

15. Prangishvili, D. *et al.* Structural and Genomic Properties of the Hyperthermophilic Archaeal Virus ATV with an Extracellular Stage of the Reproductive Cycle. *Journal of Molecular Biology* **359**, 1203–1216 (2006).
16. Svirskaitė, J., Oksanen, H. M., Daugelavičius, R. & Bamford, D. H. Monitoring physiological changes in haloarchaeal cell during virus release. *Viruses* **8**, (2016).
17. Turgeman-Grott, I. *et al.* A new archaeal virus that suppresses the transcription of host immunity genes. Preprint at https://doi.org/10.1101/2024.02.12.579488 (2024).
18. Kandiba, L., Guan, Z. & Eichler, J. Lipid modification gives rise to two distinct *Haloferax volcanii* S-layer glycoprotein populations. *Biochimica et Biophysica Acta (BBA) - Biomembranes* **1828**, 938–943 (2013).
19. Alarcón-Schumacher, T., Naor, A., Gophna, U. & Erdmann, S. Isolation of a virus causing a chronic infection in the archaeal model organism Haloferax volcanii reveals antiviral activities of a provirus. *Proceedings of the National Academy of Sciences* **119**, e2205037119 (2022).
20. Bath, C. & Dyall-Smith, M. L. His1, an Archaeal Virus of theFuselloviridae Family That Infects Haloarcula hispanica. *Journal of Virology* **72**, 9392–9395 (1998).
21. Geslin, C. *et al.* PAV1, the first virus-like particle isolated from a hyperthermophilic euryarchaeote, 'Pyrococcus abyssi'. *Journal of Bacteriology* **185**, 3888–3894 (2003).
22. Quemin, E. R. J. *et al.* Sulfolobus Spindle-Shaped Virus 1 Contains Glycosylated Capsid Proteins, a Cellular Chromatin Protein, and Host-Derived Lipids. *J Virol* **89**, 11681–11691 (2015).
23. Brumfield, S. K. *et al.* Particle Assembly and Ultrastructural Features Associated with Replication of the Lytic Archaeal Virus Sulfolobus Turreted Icosahedral Virus. *Journal of Virology* **83**, 5964–5970 (2009).
24. Fu, C. & Johnson, J. E. Structure and cell biology of archaeal virus STIV. *Current Opinion in Virology* **2**, 122–127 (2012).
25. Jäälinoja, H. T. *et al.* Structure and host-cell interaction of SH1, a membrane-containing, halophilic euryarchaeal virus. *Proceedings of the National Academy of Sciences of the United States of America* **105**, 8008–8013 (2008).
26. Quemin, E. R. J. *et al.* Eukaryotic-like virus budding in archaea. *mBio* **7**, (2016).
27. Bautista, M. A., Zhang, C. & Whitaker, R. J. Virus-Induced Dormancy in the Archaeon Sulfolobus islandicus. *mBio* **6**, 10.1128/mbio.02565-14 (2015).
28. Bailey, T. L. & Elkan, C. Fitting a mixture model by expectation maximization to discover motifs in biopolymers. *Proc Int Conf Intell Syst Mol Biol* **2**, 28–36 (1994).
29. Gruber, A. R., Lorenz, R., Bernhart, S. H., Neuböck, R. & Hofacker, I. L. The Vienna RNA Websuite. *Nucleic Acids Res* **36**, W70–W74 (2008).
30. Sonnhammer, E. L., von Heijne, G. & Krogh, A. A hidden Markov model for predicting transmembrane helices in protein sequences. *Proc Int Conf Intell Syst Mol Biol* **6**, 175–182 (1998).

31. Krogh, A., Larsson, B., von Heijne, G. & Sonnhammer, E. L. L. Predicting transmembrane protein topology with a hidden markov model: application to complete genomes1. *Journal of Molecular Biology* **305**, 567–580 (2001).
