# Supplementary material for: Two coinfecting archaeal viruses provide insights into virus-virus interactions
Source: Commun Biol. 2026 Sep 24;9:1250. doi: 10.1038/s42003-026-11061-7 (PMC13612827; doi:10.1038/s42003-026-11061-7)
Supplement: Supplementary file 3 [file 42003_2026_11061_MOESM3_ESM.pdf]

Tale of Two viruses: Two coinfecting archaeal viruses provide insights into virus-virus interactions

Description of Additional Supplementary Files

Supplementary Data files

Supplementary Data file 1: Supplementary Tables 1, 2, 4, 5, 6, 7 and 8 with each table presented in a separate tab. Provided as .xlsx.

Supplementary Data file 2: Numerical source data for graphs and charts. Each dataset is presented in a separate tab and labelled with the corresponding figure number. Provided as .xlsx.
